# Supplementary material for: The RNA-binding protein landscapes differ between mammalian organs and cultured cells
Source: Nat Commun. 2023 Apr 12;14:2074. doi: 10.1038/s41467-023-37494-w (PMC10097726; doi:10.1038/s41467-023-37494-w)
Supplement: Supplementary file 1 — Supplementary Information [file 41467_2023_37494_MOESM1_ESM.pdf]

Supplementary Information

The RNA-binding protein landscapes differ between mammalian organs and cultured cells

Supplementary Figure 1-8

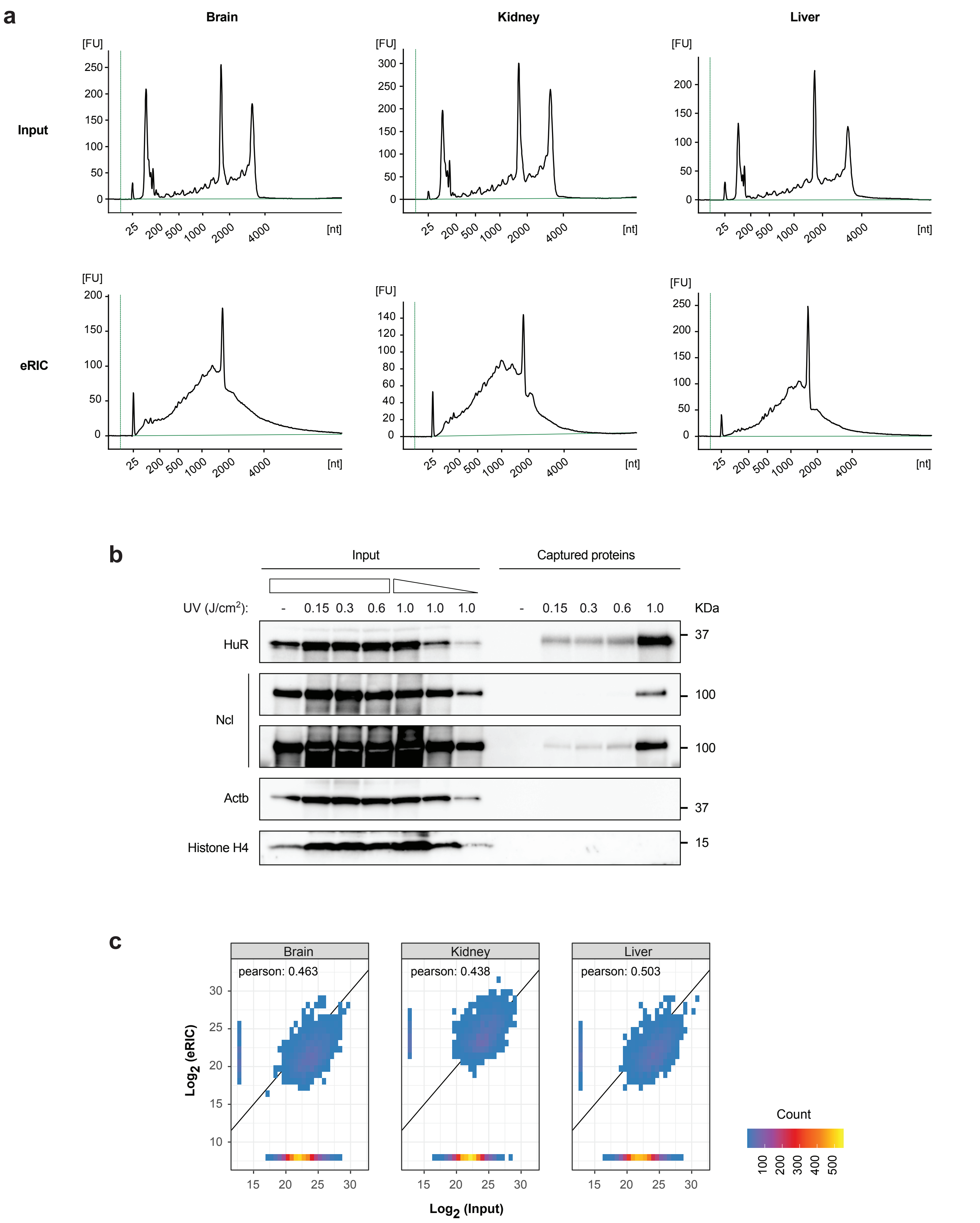

Supplementary Figure 1. Technical aspects of ex vivo eRIC. a Representative capillary electrophoresis-based analysis of the RNA material isolated from inputs (top) or by ex vivo eRIC from brain, kidney and liver (bottom). The sharp peak of 25 nt corresponds to the marker. [nt] length of RNA in number of nucleotides, [FU] fluorescence units. **b** Liver sections were exposed to increasing doses of UV light as indicated, and protein-poly(A)RNA complexes were isolated by RNA-interactome capture. The proteins obtained (indicated as eluate) were separated by SDS-PAGE and analysed by western blotting, using antibodies against the positive control RBPs Hu-antigen R (HuR) and Nucleolin (Ncl), and the negative controls Beta-actin (Actb) and Histone H4. For comparison, the matching input samples were also analysed, including a serial dilution of the input sample corresponding to the higher dose of UV. Representative results from two biologically independent experiments are presented. **c** Normalized signal sums of eRIC eluates (y-axis) versus input samples (x-axis). Note the poor correlation.

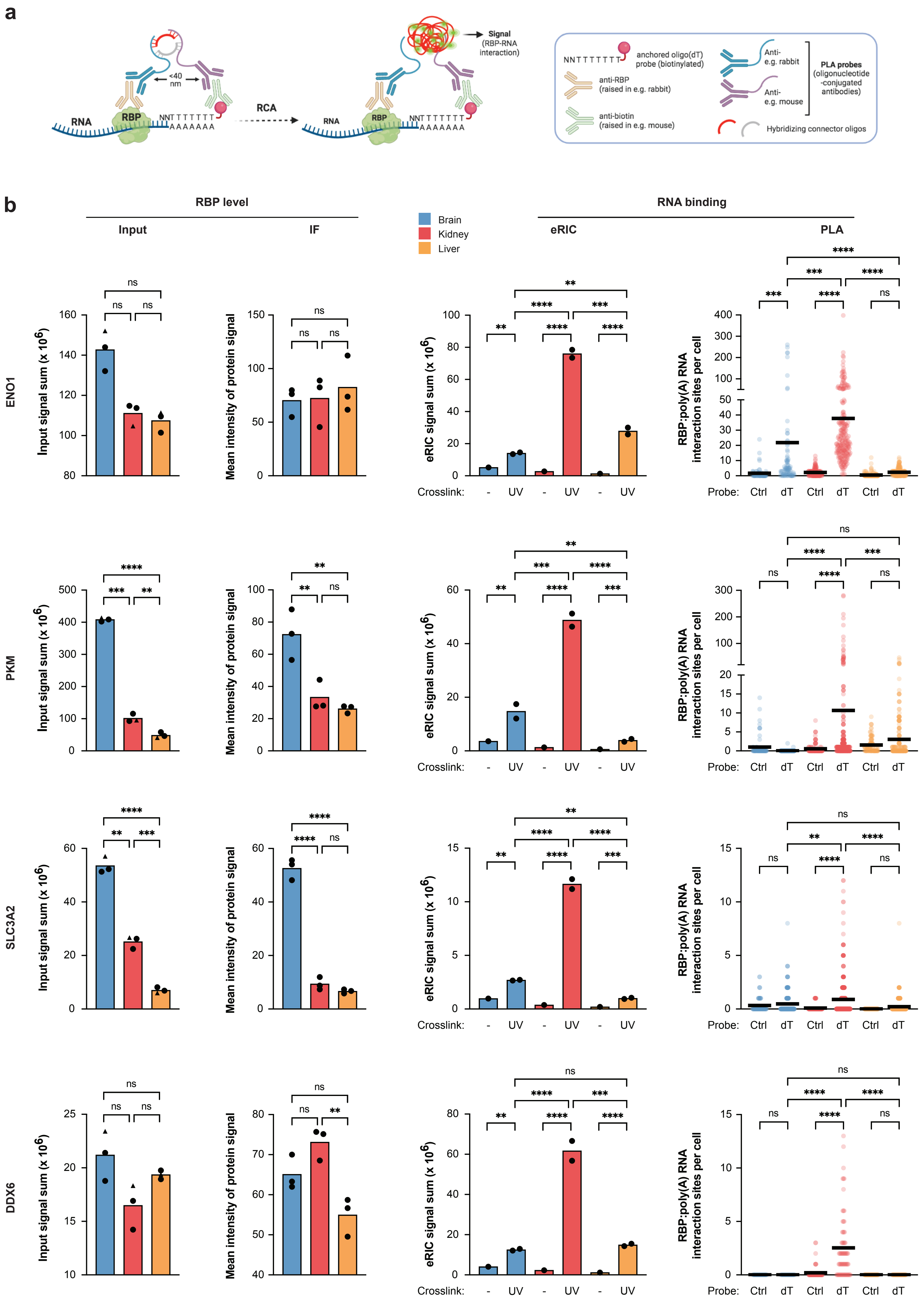
a
RNA
RBP
<40 nm
NNTTTTTTT
AAAAAAA
RCA
Signal (RBP-RNA interaction)
anchored oligo(dT) probe (biotinylated)
anti-RBP (raised in e.g. rabbit)
anti-biotin (raised in e.g. mouse)
Anti-e.g. rabbit
Anti-e.g. mouse
PLA probes (oligonucleotide -conjugated antibodies)
Hybridizing connector oligos
b
RBP level
RNA binding
Input
IF
eRIC
PLA
Brain
Kidney
Liver
ENO1
PKM
SLC3A2
DDX6
Input signal sum (x 10^6)
Mean intensity of protein signal
eRIC signal sum (x 10^6)
RBP:poly(A) RNA interaction sites per cell
Crosslink: - UV - UV - UV
Probe: Ctrl dT Ctrl dT Ctrl dT
ns
**
***
****

Supplementary Figure 2. Validation of organ-specific differences in RNA binding by PLA. **a** Schematic representation of the proximity ligation assay (PLA) for RNA-protein interactions employed to validate the organ-specific activities identified by ex vivo eRIC of four selected RBPs. Organ sections were fixed and subsequently incubated with i) a biotinylated "anchored" oligo(dT) probe, ii) antibodies raised in different species against the target RBP and biotin, and iii) secondary antibodies conjugated to PLA oligonucleotide probes. If the two probes are sufficiently close (<40 nm), they are circularized and used as templates for localized rolling-circle amplification (RCA). The amplification product is finally detected by hybridization of fluorescently labelled complementary oligonucleotides. Created with BioRender.com. **b** Normalized MS signal sum of indicated RBPs in input samples (left panel) and eRIC eluates (third panel from the left) for each organ analyzed. *FDR < 0.05, **FDR<0.01, ***FDR<0.001, ****FDR<0.0001 (*t*-test with FDR correction for multiple testing). In left panel, circles: UV-irradiated samples, triangle: non-irradiated control. Second panel: Total protein intensity assessed by immunofluorescence (IF); right panel: number of interaction sites per cell between the indicated RBP and the oligo(dT) probe (visualized as distinct dots in the PLA). Blue, brain; red, kidney; orange, liver. *p.adj < 0.05, **p.adj<0.01, ***p.adj<0.001, ****p.adj<0.0001 (one-way ANOVA with Tukey post-hoc test). Horizontal lines represent arithmetic means. For each organ, four +UV eRIC eluates were generated, each derived from a single mouse; eRIC eluates from two mice were combined, rendering $n = 2$. Organ sections from four mice were pooled to generate one -UV eRIC eluate per organ ($n = 1$). Input, PLA and IF: $n = 3$ biologically independent experiments. Source data are provided as a Source Data file.

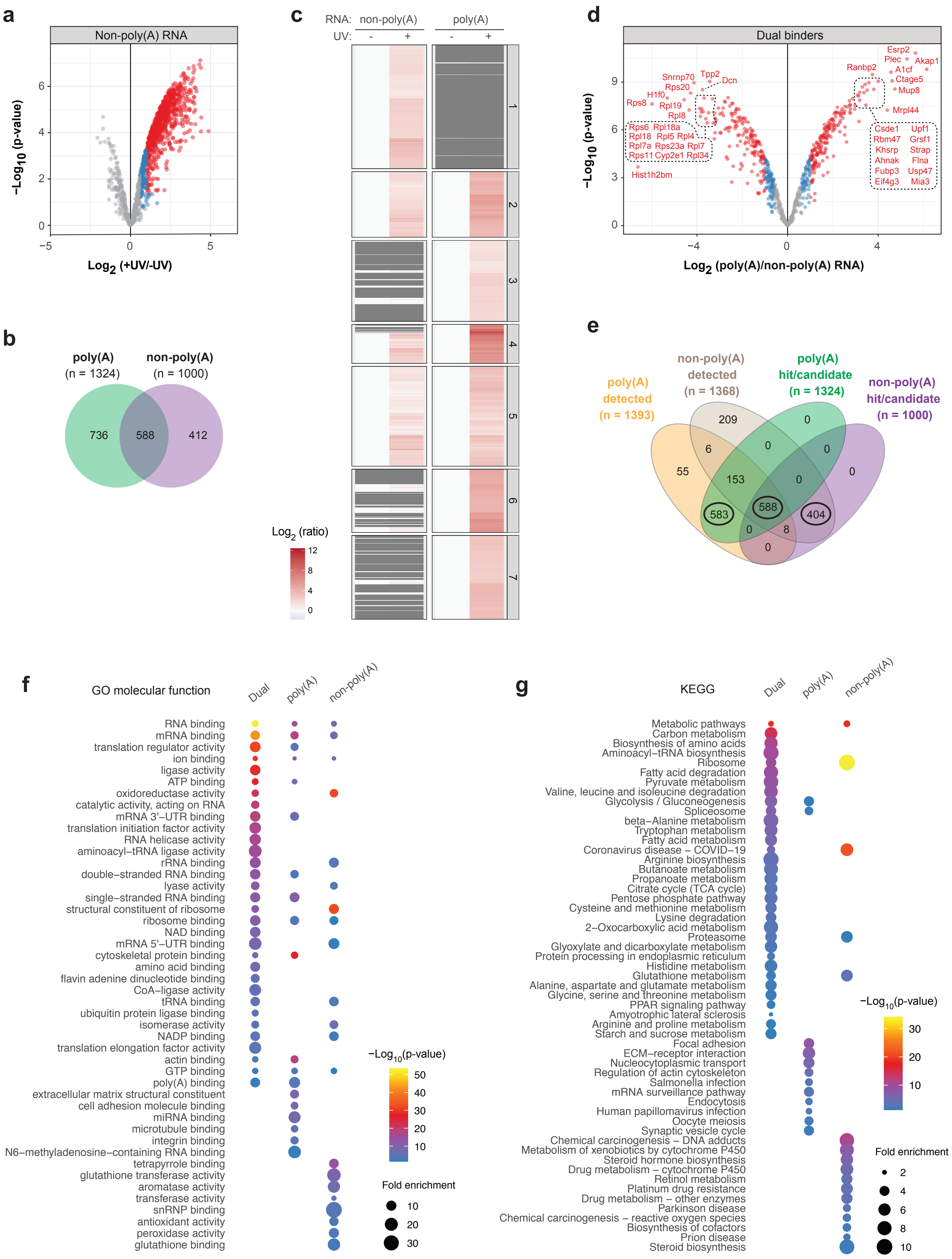
a
Non-poly(A) RNA
−Log10 (p-value)
Log2 (+UV/-UV)
b
poly(A)
(n = 1324)
non-poly(A)
(n = 1000)
736
588
412
c
RNA: non-poly(A) poly(A)
UV: - + - +
Log2 (ratio)
d
Dual binders
−Log10 (p-value)
Log2 (poly(A)/non-poly(A) RNA)
Esrp2 Plec Akap1 A1cf Ctage5 Mup8 Ranbp2 Mrpl44
Csde1 Upf1 Rbm47 Grsf1 Khsrp Strap Ahnak Flna Fubp3 Usp47 Eif4g3 Mia3
Snrnp70 Tpp2 Dcn Rps20 H1f0 Rps8 Rpl19 Rpl8
Rps6 Rpl18a Rpl18 Rpl5 Rpl4 Rpl7a Rps23a Rpl7 Rps11 Cyp2e1 Rpl34
Hist1h2bm
e
poly(A) detected (n = 1393)
non-poly(A) detected (n = 1368)
poly(A) hit/candidate (n = 1324)
non-poly(A) hit/candidate (n = 1000)
f
GO molecular function
Dual
poly(A)
non-poly(A)
RNA binding
mRNA binding
translation regulator activity
ion binding
ligase activity
ATP binding
oxidoreductase activity
catalytic activity, acting on RNA
mRNA 3′−UTR binding
translation initiation factor activity
RNA helicase activity
aminoacyl−tRNA ligase activity
rRNA binding
double−stranded RNA binding
lyase activity
single−stranded RNA binding
structural constituent of ribosome
ribosome binding
NAD binding
mRNA 5′−UTR binding
cytoskeletal protein binding
amino acid binding
flavin adenine dinucleotide binding
CoA−ligase activity
tRNA binding
ubiquitin protein ligase binding
isomerase activity
NADP binding
translation elongation factor activity
actin binding
GTP binding
poly(A) binding
extracellular matrix structural constituent
cell adhesion molecule binding
miRNA binding
microtubule binding
integrin binding
N6−methyladenosine−containing RNA binding
tetrapyrrole binding
glutathione transferase activity
aromatase activity
transferase activity
snRNP binding
antioxidant activity
peroxidase activity
glutathione binding
−Log10(p-value)
Fold enrichment
g
KEGG
Metabolic pathways
Carbon metabolism
Biosynthesis of amino acids
Aminoacyl−tRNA biosynthesis
Ribosome
Fatty acid degradation
Pyruvate metabolism
Valine, leucine and isoleucine degradation
Glycolysis / Gluconeogenesis
Spliceosome
beta−Alanine metabolism
Tryptophan metabolism
Fatty acid metabolism
Coronavirus disease − COVID−19
Arginine biosynthesis
Butanoate metabolism
Propanoate metabolism
Citrate cycle (TCA cycle)
Pentose phosphate pathway
Cysteine and methionine metabolism
Lysine degradation
2−Oxocarboxylic acid metabolism
Proteasome
Glyoxylate and dicarboxylate metabolism
Protein processing in endoplasmic reticulum
Histidine metabolism
Glutathione metabolism
Alanine, aspartate and glutamate metabolism
Glycine, serine and threonine metabolism
PPAR signaling pathway
Amyotrophic lateral sclerosis
Arginine and proline metabolism
Starch and sucrose metabolism
Focal adhesion
ECM−receptor interaction
Nucleocytoplasmic transport
Regulation of actin cytoskeleton
Salmonella infection
mRNA surveillance pathway
Endocytosis
Human papillomavirus infection
Oocyte meiosis
Synaptic vesicle cycle
Chemical carcinogenesis − DNA adducts
Metabolism of xenobiotics by cytochrome P450
Steroid hormone biosynthesis
Drug metabolism − cytochrome P450
Retinol metabolism
Platinum drug resistance
Drug metabolism − other enzymes
Parkinson disease
Chemical carcinogenesis − reactive oxygen species
Biosynthesis of cofactors
Prion disease
Steroid biosynthesis
−Log10(p-value)
Fold enrichment

Supplementary Figure 3. In-depth characterization of the non-poly(A) RNA-bound proteome of liver. a Volcano plot showing significant enrichment of RBPs in irradiated over non-irradiated samples. Red dots, hits: FDR<0.05, FC>2. Blue dots, candidates: FDR<0.2, FC>1.5 (moderated two-sided *t*-test with FDR multiple testing correction). **b** Venn diagram depicting the number of proteins interacting exclusively with poly(A) RNA or non-poly(A) RNA, or with both biotypes of RNA. **c** Hierarchical clustering and heatmap of the poly(A) and non-poly(A) RBPs identified in liver, shown as the Log.2 ratio of protein abundance in irradiated versus non-irradiated samples. **d** Volcano plot showing relative enrichment of RBPs in poly(A) RNA- and non-poly(A) RNA-bound proteomes. Shown as the Log.2 ratio of protein abundance in eRIC versus non-poly(A)RIC irradiated samples. Red dots, hits: FDR<0.05, FC>2. Blue dots, candidates: FDR<0.2, FC>1.5 (moderated two-sided *t*-test with FDR multiple testing correction). The names of representative RBPs exhibiting the largest FC are indicated. **e** Venn diagram depicting the number of proteins detected or scoring as hits/candidates in ex vivo eRIC and/or non-poly(A)RIC in liver. Dual binders are hits/candidates in both datasets (n = 588). Exclusive poly(A) RNA binders are eRIC hits/candidates not detected in non-poly(A)RIC eluates (n = 583). Conversely, exclusive non-poly(A) RNA binders are non-poly(A)RIC hits/candidates not detected in eRIC eluates (n = 404). GO (**f**) and KEGG (**g**) enrichment analyses performed on dual, poly(A) and non-poly(A) RNA binders (Fisher's one-tailed test with g:SCS multiple testing correction). Selected GO terms corresponding to molecular function are shown (see Supplementary Data 9 for the full list of GO terms). Four +UV eRIC eluates were generated, each derived from a single mouse; eRIC eluates from two mice were combined, rendering n = 2. Organ sections from four mice were pooled to generate one -UV eRIC eluate (n = 1). non-poly(A)RIC, n = 4 biologically independent experiments.

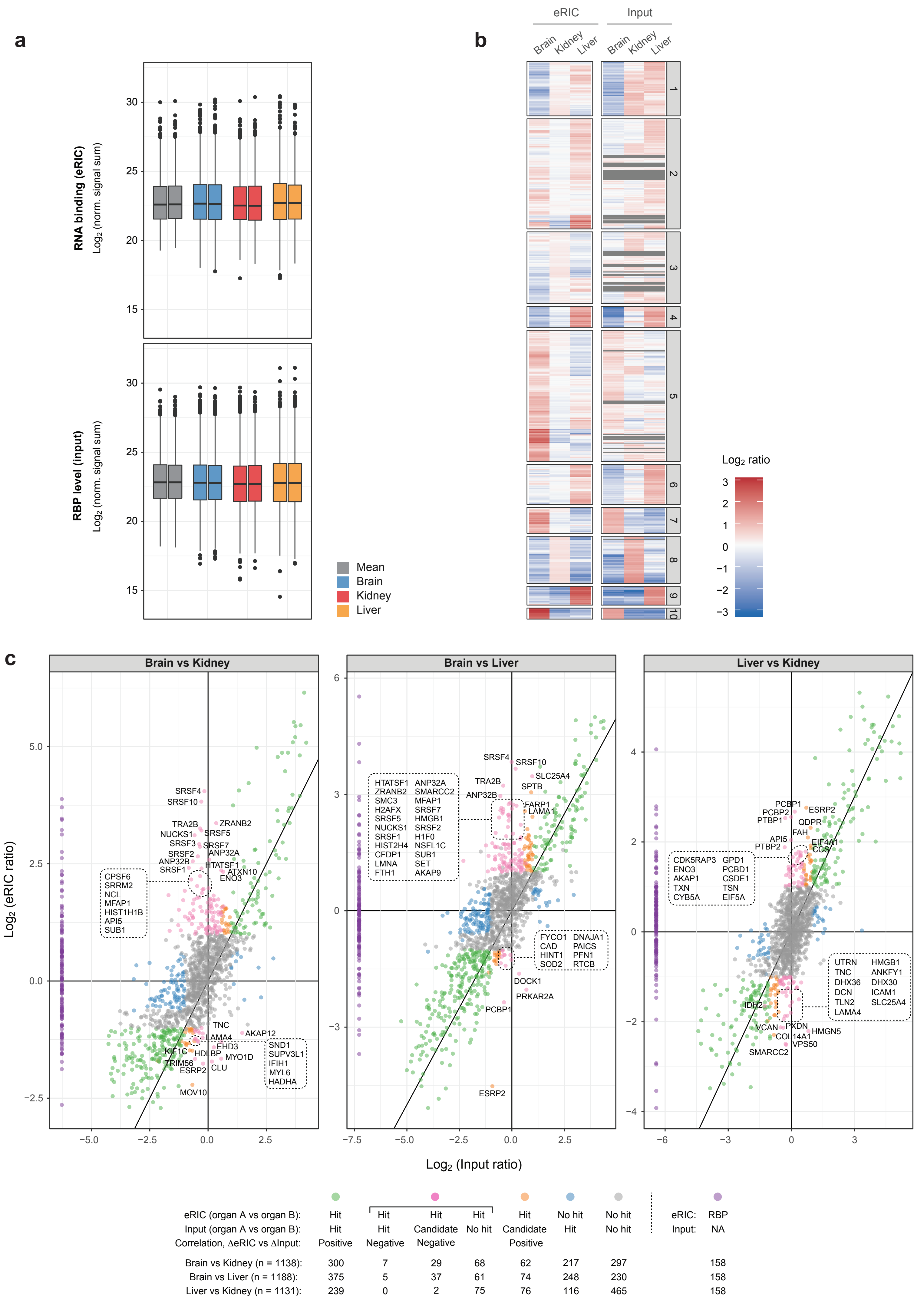
a
RNA binding (eRIC)
Log2 (norm. signal sum)
RBP level (input)
Log2 (norm. signal sum)
30
25
20
15
Mean
Brain
Kidney
Liver
b
eRIC
Input
Brain Kidney Liver
Brain Kidney Liver
1
2
3
4
5
6
7
8
9
10
Log2 ratio
3
2
1
0
−1
−2
−3
c
Brain vs Kidney
Brain vs Liver
Liver vs Kidney
Log2 (eRIC ratio)
Log2 (Input ratio)
SRSF4
SRSF10
TRA2B
ZRANB2
NUCKS1
SRSF5
SRSF3
SRSF7
SRSF2
ANP32A
ANP32B
SRSF1
HTATSF1
ATXN10
ENO3
CPSF6
SRRM2
NCL
MFAP1
HIST1H1B
API5
SUB1
TNC
LAMA4
AKAP12
KIF1C
HDLBP
EHD3
TRIM56
MYO1D
ESRP2
CLU
SND1
SUPV3L1
IFIH1
MYL6
HADHA
MOV10
HTATSF1 ANP32A
ZRANB2 SMARCC2
SMC3 MFAP1
H2AFX SRSF7
SRSF5 HMGB1
NUCKS1 SRSF2
SRSF1 H1F0
HIST2H4 NSFL1C
CFDP1 SUB1
LMNA SET
FTH1 AKAP9
SRSF4
SRSF10
SLC25A4
TRA2B
SPTB
ANP32B
FARP1
LAMA1
FYCO1 DNAJA1
CAD PAICS
HINT1 PFN1
SOD2 RTCB
DOCK1
PRKAR2A
PCBP1
ESRP2
PCBP1
PCBP2
ESRP2
PTBP1
QDPR
FAH
API5
PTBP2
EIF4A1
CCS
CDK5RAP3 GPD1
ENO3 PCBD1
AKAP1 CSDE1
TXN TSN
CYB5A EIF5A
UTRN HMGB1
TNC ANKFY1
DHX36 DHX30
DCN ICAM1
TLN2 SLC25A4
LAMA4
IDH2
VCAN
PXDN
HMGN5
COL14A1
VPS50
SMARCC2
5.0
2.5
0.0
−2.5
−5.0
6
3
0
−3
−7.5
4
2
−2
−4
−6
eRIC (organ A vs organ B): Hit | Hit | Hit | Hit | Hit | No hit | No hit | eRIC: RBP
Input (organ A vs organ B): Hit | Hit | Candidate | No hit | Candidate | Hit | No hit | Input: NA
Correlation, ΔeRIC vs ΔInput: Positive | Negative | Negative | | Positive
Brain vs Kidney (n = 1138): 300 | 7 | 29 | 68 | 62 | 217 | 297 | 158
Brain vs Liver (n = 1188): 375 | 5 | 37 | 61 | 74 | 248 | 230 | 158
Liver vs Kidney (n = 1131): 239 | 0 | 2 | 75 | 76 | 116 | 465 | 158

Supplementary Figure 4. Organ-specific differences of RBP activity. **a-c** Data were analyzed assuming equal mean signal intensity across the poly(A) RNA-bound proteomes studied. **a** Normalized signal sum of identified RBPs in each of the two crosslinked ex vivo eRIC eluates (upper panel) and input samples (bottom panel). Blue, brain; red, kidney; orange, liver. Center lines indicate medians, box borders represent the interquartile range (IQR), and whiskers extend to ±1.5 time the IQR; outliers are shown as black dots. **b** Hierarchical clustering and heatmap of the RBPs identified from brain, kidney, and liver, depicting protein abundance in eRIC (left columns) and input samples (right columns) (shown as the Log.2 ratio of protein abundance in each eRIC or input sample relative to the average protein abundance in corresponding eRIC and input samples). **c** Pairwise comparison (expressed as Log.2 ratio) of RBP signal intensities in eRIC eluates (y-axis) and input samples (x-axis) across the three organs analyzed. The names of representative RBPs exhibiting differences in RNA-binding but no corresponding change in overall protein abundance are indicated; when dot density is too high, the corresponding protein names are indicated inside rounded rectangles. Purple dots correspond to RBPs detected in eRIC eluates but giving no signal in input; an artificial input intensity of low magnitude was imputed to these proteins in order to incorporate them in the graphic. **a-c** For each organ, four +UV eRIC eluates were generated, each derived from a single mouse; eRIC eluates from two mice were combined, rendering *n* = 2. Organ sections from four mice were pooled to generate one -UV eRIC eluate per organ (*n* = 1).

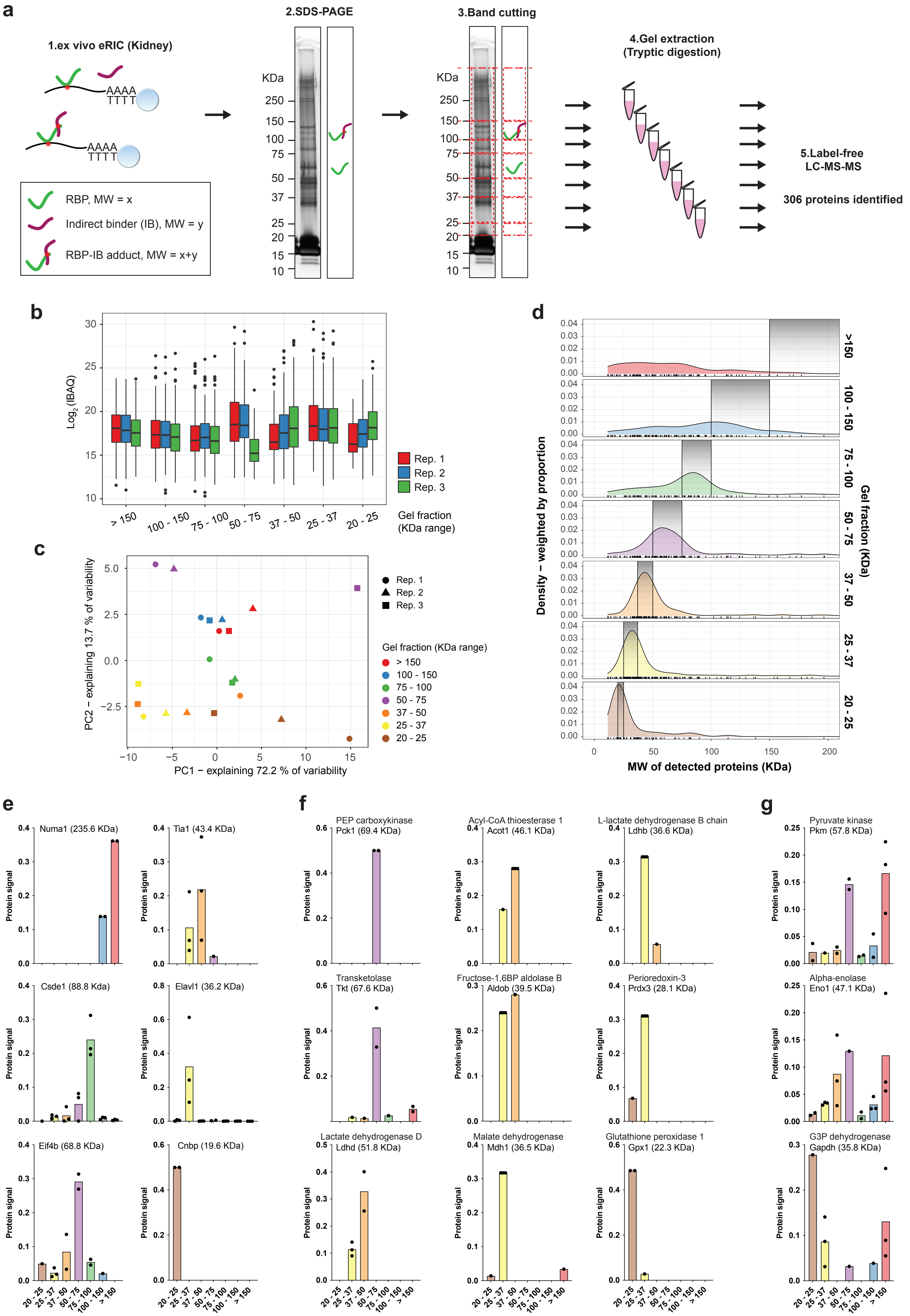
a
1.ex vivo eRIC (Kidney)
AAAA
TTTT
RBP, MW = x
Indirect binder (IB), MW = y
RBP-IB adduct, MW = x+y
2.SDS-PAGE
3.Band cutting
KDa
250
150
100
75
50
37
25
20
15
10
4.Gel extraction
(Tryptic digestion)
5.Label-free
LC-MS-MS
306 proteins identified
b
Log2 (IBAQ)
Rep. 1
Rep. 2
Rep. 3
Gel fraction
(KDa range)
> 150
100 - 150
75 - 100
50 - 75
37 - 50
25 - 37
20 - 25
c
PC2 − explaining 13.7 % of variability
PC1 − explaining 72.2 % of variability
Gel fraction (KDa range)
d
Density − weighted by proportion
MW of detected proteins (KDa)
Gel fraction (KDa)
e
Numa1 (235.6 KDa)
Tia1 (43.4 KDa)
Csde1 (88.8 Kda)
Elavl1 (36.2 KDa)
Eif4b (68.8 KDa)
Cnbp (19.6 KDa)
Protein signal
f
PEP carboxykinase
Pck1 (69.4 KDa)
Acyl-CoA thioesterase 1
Acot1 (46.1 KDa)
L-lactate dehydrogenase B chain
Ldhb (36.6 KDa)
Transketolase
Tkt (67.6 KDa)
Fructose-1,6BP aldolase B
Aldob (39.5 KDa)
Perioredoxin-3
Prdx3 (28.1 KDa)
Lactate dehydrogenase D
Ldhd (51.8 KDa)
Malate dehydrogenase
Mdh1 (36.5 KDa)
Glutathione peroxidase 1
Gpx1 (22.3 KDa)
g
Pyruvate kinase
Pkm (57.8 KDa)
Alpha-enolase
Eno1 (47.1 KDa)
G3P dehydrogenase
Gapdh (35.8 KDa)

Supplementary Figure 5. Global assessment of protein-protein crosslinks in ex vivo eRIC eluates. a Schematic representation of the experimental approach to unbiasedly assess protein-protein crosslinks in eRIC eluates. The proteins captured by ex vivo eRIC from kidney were separated according to their molecular weight (MW) by SDS polyacrylamide gel electrophoresis. The gel was divided into 7 fractions of defined MW that were subjected to label-free MS, and the distribution of each protein across the fractions was determined. Proteins that have formed protein-protein crosslinks should shift to higher MW fractions. **b** Protein signal (iBAQ) across gel fractions and replicates. Center lines indicate medians, box borders represent the interquartile range (IQR), and whiskers extend to ±1.5 time the IQR; outliers are shown as black dots. **c** Principal component (PC) analysis of identified proteins. **d** Density plot depicting the distribution of observed MW of identified proteins for each gel fraction. The density is weighted by the proportion of abundance of each identified protein (iBAQ values divided by the sum of all observed iBAQ values in all gel fractions per protein). **e, f** Distribution profiles of representative examples of canonical (**e**) and enzyme-RBPs (**f**) of different MW across gel fractions. **g** Profiles of enzyme-RBPs displaying a bimodal distribution. PEP, phosphoenolpyruvate; Fructose-1,6BP, beta-D-fructose 1,6-biphosphate; G3P, glyceraldehyde 3-phosphate. **b**-**g** n = 3 biologically independent experiments. Source data are provided as a Source Data file.

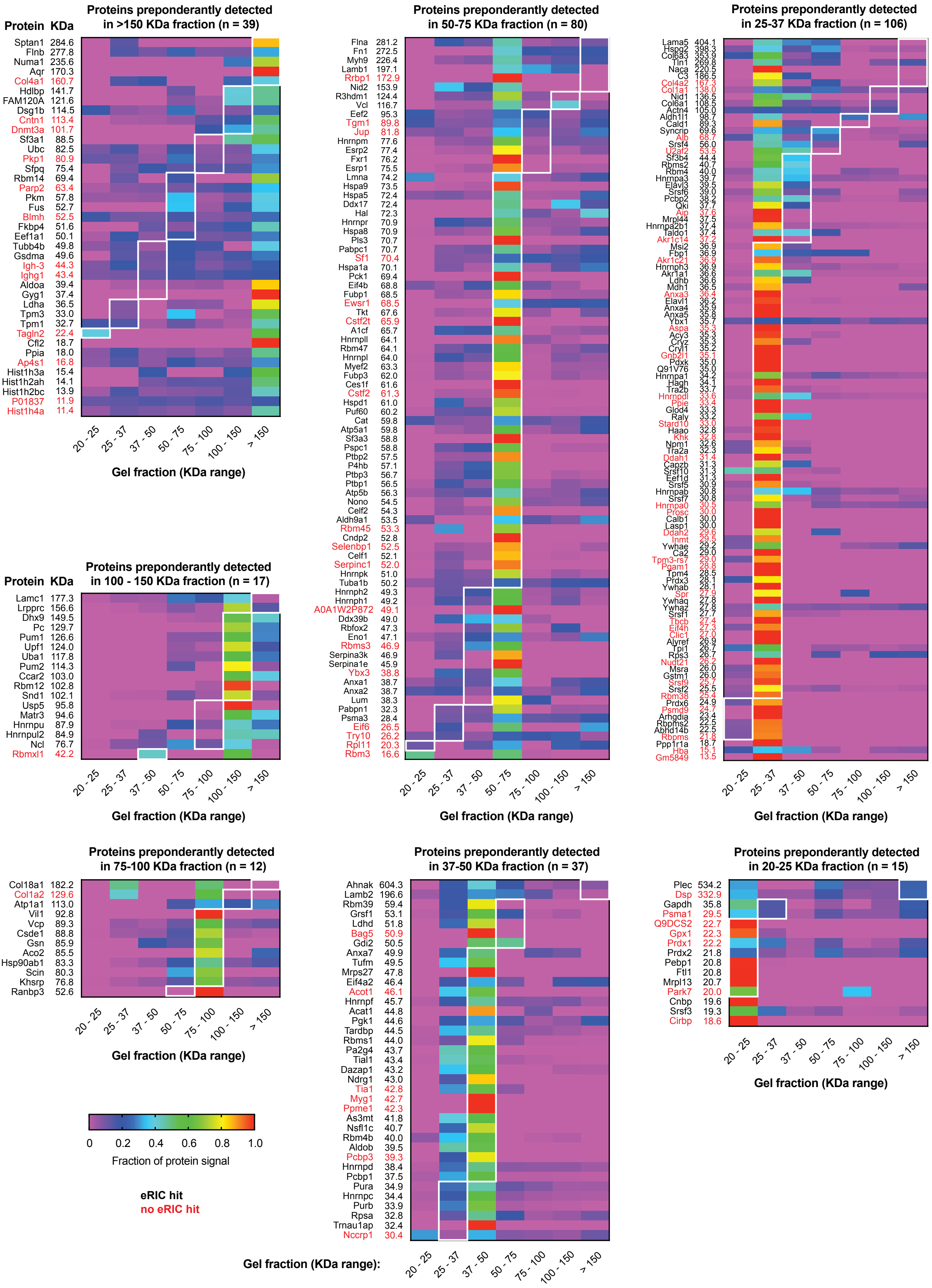

Supplementary Figure 6. Global assessment of protein-protein crosslinks in ex vivo eRIC eluates. Distribution profiles of identified RBPs across gel fractions. Proteins were classified according to the fraction in which they were preponderantly detected. White rectangles indicate protein locations expected from the predicted monomeric molecular mass. Protein names in black and red correspond to proteins that scored or not, respectively, as eRIC hits in at least one studied organ. *n* = 3 biologically independent experiments.

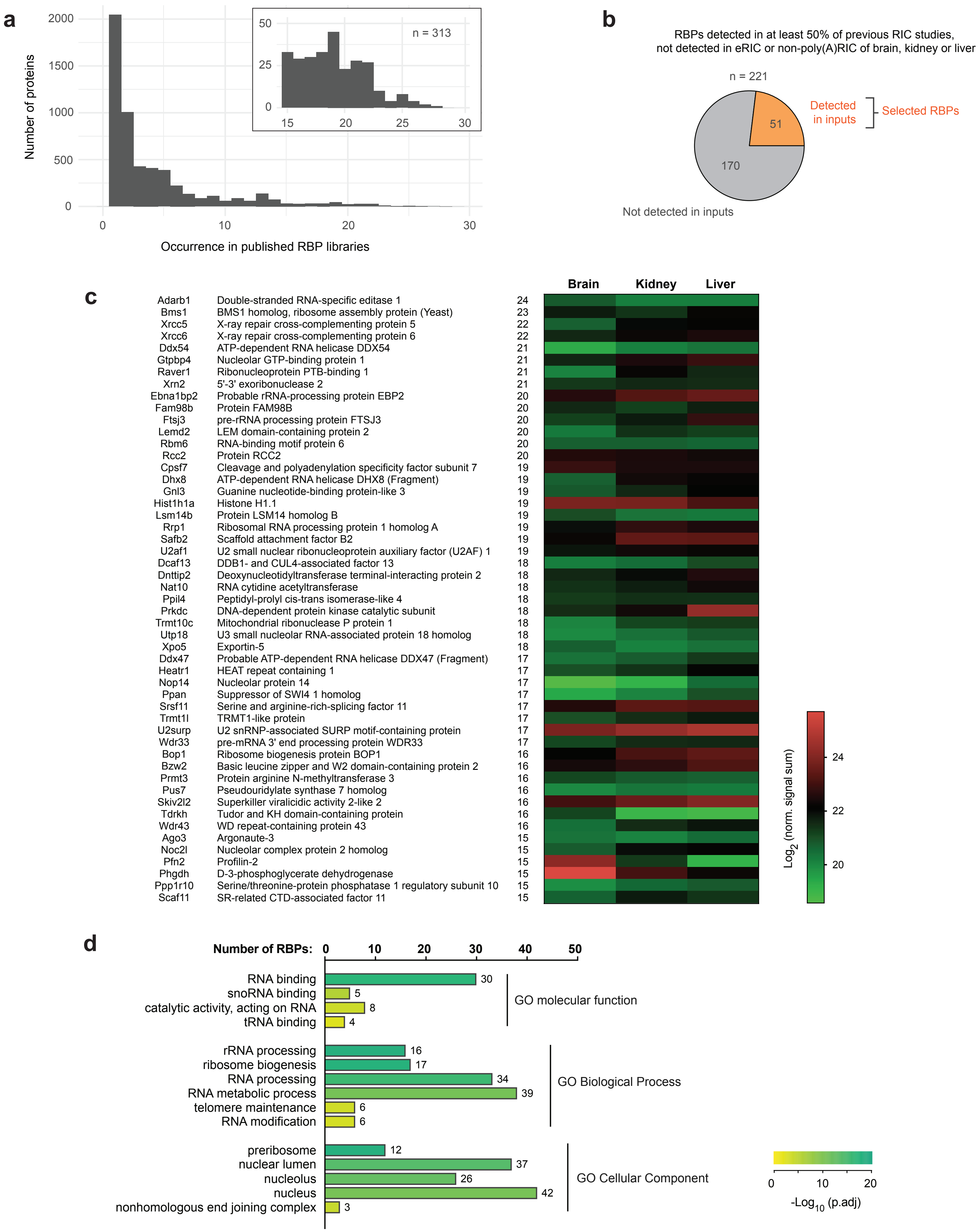

Supplementary Figure 7. The RNA-binding proteomes of brain, kidney and liver lack RBPs commonly identified in cultured cells. The eRIC dataset was compared to a combined list of 6518 RBPs from 30 published RBP libraries generated from cultured cells. **a** Number of RBPs (y-axis) identified across an increasing number of studies (x-axis) that were not detected in mouse organs by ex vivo eRIC. **b** Proteins not detected in eRIC or non-poly(A)RIC eluates in organs and detected in at least 50% of previous RBP libraries were classified into two groups based on whether or not they were detected in inputs. The “selected RBPs” were employed for further analysis. **c** Gene and protein names of the selected RBPs, depicting their expression levels in brain, kidney and liver. Numbers next to RBP names are the number of cell lines studies (total: 30) where the proteins were hits. **d** GO-term enrichment of the RBPs selected in **b** (Fisher's one-tailed test with g:SCS multiple testing correction). The number of RBPs in each category is shown.

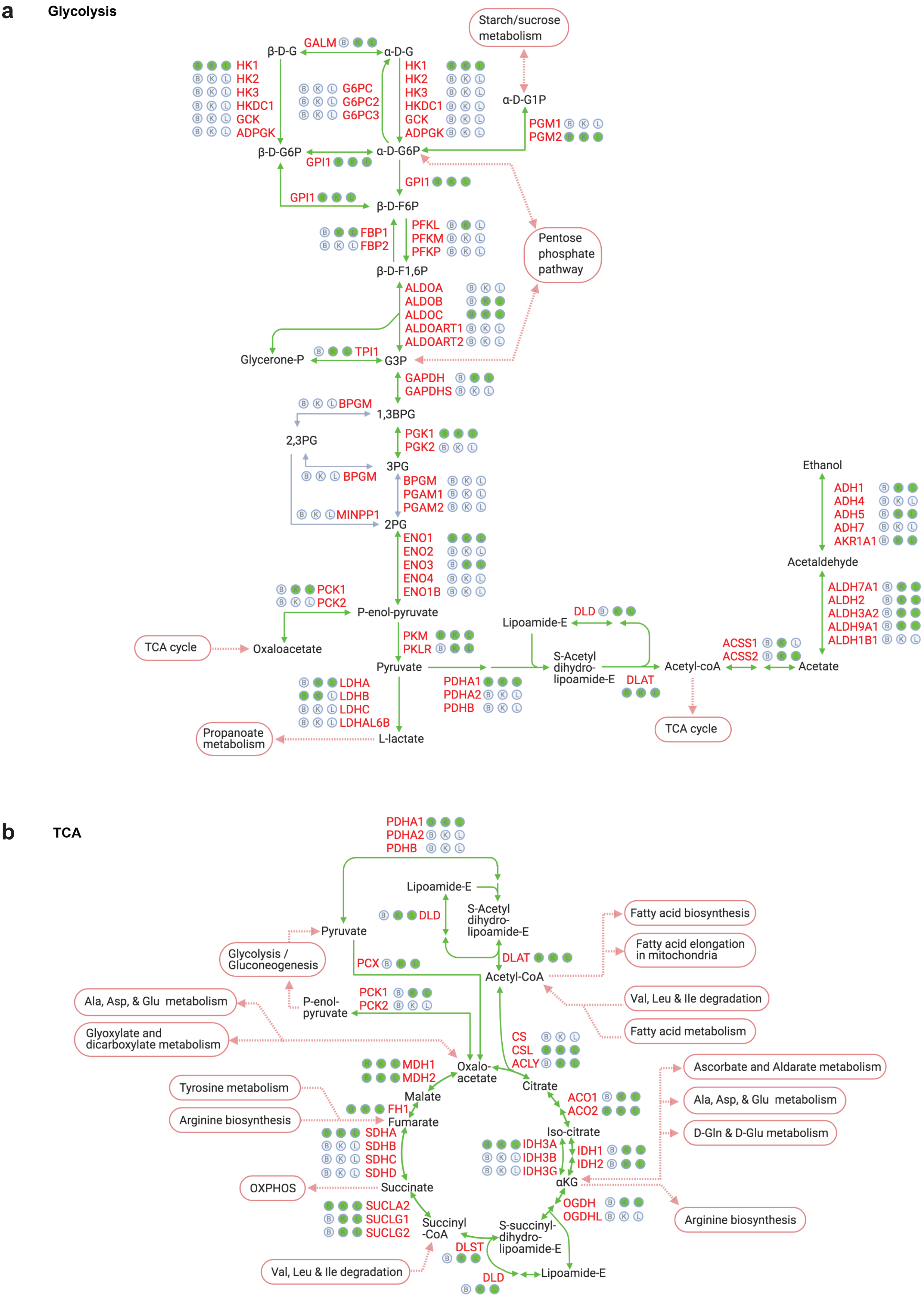

Supplementary Figure 8. Enzyme-RBPs in energy metabolism. **a, b** Analysis as in Figure 6b for glycolysis (**a**) and the tricarboxylic acid (TCA) cycle (**b**).
