# Supplementary material for: The RNA-binding protein landscapes differ between mammalian organs and cultured cells
Source: Nat Commun. 2023 Apr 12;14:2074. doi: 10.1038/s41467-023-37494-w (PMC10097726; doi:10.1038/s41467-023-37494-w)
Supplement: Supplementary file 3 — Description of Additional Supplementary Files [file 41467_2023_37494_MOESM3_ESM.docx]

**Description of Additional Supplementary Files**

File Name: Supplementary Data 1

Description: ex vivo eRIC and total proteome of mouse brain, kidney and liver. Also: occurrence of the detected organ RBPs in previous studies; occurrence of novel RBPs identified in organs in total proteomes of cell lines; list of RBPs found in cultured cells that were not detected in eRIC eluates from organs.

File Name: Supplementary Data 2

Description: Pairwise comparison across brain, kidney and liver of protein intensities in eRIC and input samples. eRIC data was normalized without assuming equal mean protein intensity across organs (related to Figure 3).

File Name: Supplementary Data 3

Description: non-poly(A)RIC of mouse brain, kidney and liver (performed with 2 µg of captured RNA per sample).

File Name: Supplementary Data 4

Description: Comprehensive non-poly(A)RIC of mouse liver (performed with 40 µg of captured RNA per sample).

File Name: Supplementary Data 5

Description: Pairwise comparison across brain, kidney and liver of protein intensities in eRIC and input samples. eRIC data was normalized assuming same mean protein intensity across organs (related to Supplementary Figure 4).

File Name: Supplementary Data 6

Description: Global assessment of protein-protein crosslinks in ex vivo eRIC eluates of mouse kidney origin.

File Name: Supplementary Data 7

Description: Protein intensities in inputs and eRIC eluates of the enzymes of intermediary metabolism (metabolite interconversion enzymes in PANTHER protein class) detected in input samples and/or eRIC eluates. The domains, catalytic activities and cofactors of each enzyme are also provided.

File Name: Supplementary Data 8

Description: Full results of gene ontology enrichment analysis of eRIC hits in brain, kidney and liver (related to Figure 2b). BP, biological process; MF, molecular function; CC, cellular component.

File Name: Supplementary Data 9

Description: Full results of gene ontology and KEGG enrichment analysis of: i) RBPs exclusively or simultaneously detected in non-poly(A) RIC or ex vivo eRIC (related to Figure 4d and Supplementary Figure 3e-g). ii) proteins identified in RBP profiling studies in cultured cells (>=50% previous reports) and in inputs of organs but not in eRIC eluates from organs (related to Supplementary Figure 7d). BP, biological process; MF, molecular function; CC, cellular component.

File Name: Supplementary Data 10

Description: List of employed InterPro domains involved in nucleotide binding (related to Figure 7c).

File Name: Supplementary Data 11

Description: Access information to the previously published datasets used in this study.
