# Supplementary material for: The RNA-binding protein landscapes differ between mammalian organs and cultured cells
Source: Nat Commun. 2023 Apr 12;14:2074. doi: 10.1038/s41467-023-37494-w (PMC10097726; doi:10.1038/s41467-023-37494-w)
Supplement: Supplementary file 11 — Supplementary Data 8 [file 41467_2023_37494_MOESM11_ESM.pdf]

GENEONTOLOGY Unifying Biology PANTHER Classification System

LOGIN REGISTER CONTACT US

Home | About | PANTHER Data | PANTHER Tools | PANTHER Services | Workspace | Downloads | Help/Tutorial

PANTHER 15.0 released!

Analysis Summary: Please report in publication

Analysis Type: PANTHER Overrepresentation Test (Released 20200407)

Annotation Version and Release Date: GO Ontology database Released 2020-02-21

Analyzed List: upload_1 (Mus musculus) Change

Reference List: Mus musculus (all genes in database) Change

Annotation Data Set: GO biological process complete

Test Type: (•) Fisher's Exact ( ) Binomial

Correction: ( ) Calculate False Discovery Rate (•) Use the Bonferroni correction for multiple testing ( ) No correction

Results

	Reference list	upload_1
Uniquely Mapped IDS:	22265 out of 22265	622 out of 622
Unmapped IDs:	0	0
Multiple mapping information:	0	0

Bonferroni count: 8894

Export Table | XML with user input ids | JSON with user input ids

Displaying only results for Bonferroni-corrected for P < 0.05, click here to display all results

	Mus musculus (REF)	upload_1 (Hierarchy NEW!)				
GO biological process complete	#	#	expected	Fold Enrichment	+/-	P value
viral translational termination-reinitiation	5	5	.14	35.80	+	2.92E-02
↳viral process	131	19	3.66	5.19	+	2.43E-04
↳symbiotic process	228	30	6.37	4.71	+	2.09E-07
↳viral translation	14	7	.39	17.90	+	7.86E-03
↳viral gene expression	20	8	.56	14.32	+	4.86E-03
positive regulation of mRNA binding	8	6	.22	26.85	+	8.72E-03
↳regulation of mRNA binding	10	7	.28	25.06	+	1.44E-03
↳regulation of RNA binding	12	8	.34	23.86	+	2.39E-04
↳regulation of molecular function	2559	111	71.49	1.55	+	4.04E-02
↳biological regulation	12152	436	339.48	1.28	+	6.40E-11
↳positive regulation of RNA binding	9	6	.25	23.86	+	1.42E-02
positive regulation of establishment of protein localization to telomere	10	7	.28	25.06	+	1.44E-03
↳positive regulation of establishment of protein localization	462	37	12.91	2.87	+	3.63E-04
↳positive regulation of biological process	6100	261	170.41	1.53	+	1.33E-10
↳regulation of biological process	11534	414	322.22	1.28	+	2.04E-09
↳regulation of establishment of protein localization	771	53	21.54	2.46	+	7.95E-05
↳regulation of protein localization	1075	77	30.03	2.56	+	2.39E-09
↳regulation of localization	2886	141	80.62	1.75	+	5.38E-07

↳positive regulation of protein localization to chromosome, telomeric region	13	7	.36	19.27	+	5.36E-03
↳regulation of protein localization to chromosome, telomeric region	15	7	.42	16.70	+	1.13E-02
↳regulation of cellular protein localization	560	54	15.64	3.45	+	3.01E-10
↳regulation of cellular localization	837	70	23.38	2.99	+	3.07E-11
↳positive regulation of cellular protein localization	308	31	8.60	3.60	+	3.93E-05
↳regulation of establishment of protein localization to telomere	11	7	.31	22.78	+	2.31E-03
↳regulation of establishment of protein localization to chromosome	12	7	.34	20.88	+	3.57E-03
tRNA aminoacylation for protein translation	40	18	1.12	16.11	+	7.47E-11
↳tRNA aminoacylation	43	18	1.20	14.98	+	2.02E-10
↳amino acid activation	44	18	1.23	14.64	+	2.77E-10
↳cellular amino acid metabolic process	231	29	6.45	4.49	+	1.24E-06
↳primary metabolic process	6269	316	175.13	1.80	+	1.20E-27
↳metabolic process	7222	351	201.76	1.74	+	1.06E-29
↳carboxylic acid metabolic process	761	52	21.26	2.45	+	1.27E-04
↳oxoacid metabolic process	803	52	22.43	2.32	+	6.25E-04
↳organic acid metabolic process	829	52	23.16	2.25	+	1.82E-03
↳cellular metabolic process	6331	332	176.86	1.88	+	1.30E-33
↳cellular process	14059	536	392.76	1.36	+	1.31E-32
↳small molecule metabolic process	1432	73	40.00	1.82	+	1.34E-02
↳organic substance metabolic process	6701	332	187.20	1.77	+	1.39E-28
↳organonitrogen compound metabolic process	4270	202	119.29	1.69	+	9.80E-11
↳nitrogen compound metabolic process	5718	301	159.74	1.88	+	5.73E-29
↳tRNA metabolic process	165	20	4.61	4.34	+	1.52E-03
↳ncRNA metabolic process	416	31	11.62	2.67	+	1.89E-02
↳RNA metabolic process	1232	126	34.42	3.66	+	4.54E-31
↳nucleic acid metabolic process	1767	141	49.36	2.86	+	8.48E-25
↳macromolecule metabolic process	5056	285	141.25	2.02	+	1.19E-31
↳nucleobase-containing compound metabolic process	2191	165	61.21	2.70	+	2.36E-27
↳cellular nitrogen compound metabolic process	2789	208	77.91	2.67	+	3.25E-36
↳organic cyclic compound metabolic process	2616	174	73.08	2.38	+	5.37E-23
↳heterocycle metabolic process	2328	171	65.04	2.63	+	2.37E-27
↳cellular aromatic compound metabolic process	2402	169	67.10	2.52	+	7.26E-25
↳translation	306	61	8.55	7.14	+	2.10E-26
↳cellular protein metabolic process	2844	161	79.45	2.03	+	4.45E-14
↳protein metabolic process	3420	171	95.54	1.79	+	2.90E-10
↳cellular macromolecule metabolic process	3958	206	110.57	1.86	+	1.25E-15
↳gene expression	1616	158	45.14	3.50	+	2.07E-38
↳peptide biosynthetic process	326	61	9.11	6.70	+	4.34E-25
↳organonitrogen compound biosynthetic process	1070	90	29.89	3.01	+	1.45E-15
↳organic substance biosynthetic process	2079	124	58.08	2.14	+	2.71E-11
↳biosynthetic process	2161	128	60.37	2.12	+	1.50E-11
↳peptide metabolic process	448	67	12.52	5.35	+	7.95E-23
↳cellular amide metabolic process	665	74	18.58	3.98	+	1.85E-18
↳amide biosynthetic process	421	64	11.76	5.44	+	5.28E-22
↳cellular nitrogen compound biosynthetic process	1118	93	31.23	2.98	+	6.56E-16
↳cellular biosynthetic process	1985	122	55.45	2.20	+	6.36E-12
↳cellular macromolecule biosynthetic process	1193	88	33.33	2.64	+	8.61E-12

↳macromolecule biosynthetic process	1227	88	34.28	2.57	+	5.55E-11
stress granule assembly	16	7	.45	15.66	+	1.58E-02
↳cellular component assembly	1941	124	54.22	2.29	+	2.42E-13
↳cellular component organization	5045	245	140.94	1.74	+	3.35E-16
↳cellular component organization or biogenesis	5239	250	146.36	1.71	+	1.04E-15
↳cellular component biogenesis	2168	130	60.57	2.15	+	2.99E-12
↳organelle organization	3054	174	85.32	2.04	+	6.32E-16
regulation of alternative mRNA splicing, via spliceosome	66	26	1.84	14.10	+	1.09E-15
↳regulation of mRNA splicing, via spliceosome	110	37	3.07	12.04	+	1.75E-21
↳regulation of RNA splicing	145	43	4.05	10.62	+	1.42E-23
↳regulation of gene expression	3827	191	106.91	1.79	+	4.06E-12
↳regulation of macromolecule metabolic process	5484	255	153.20	1.66	+	9.63E-15
↳regulation of metabolic process	5976	270	166.95	1.62	+	2.31E-14
↳regulation of RNA metabolic process	3165	147	88.42	1.66	+	6.05E-06
↳regulation of nucleobase-containing compound metabolic process	3401	161	95.01	1.69	+	1.49E-07
↳regulation of primary metabolic process	5349	244	149.43	1.63	+	1.17E-12
↳regulation of nitrogen compound metabolic process	5198	242	145.21	1.67	+	1.59E-13
↳regulation of cellular metabolic process	5552	254	155.10	1.64	+	1.07E-13
↳regulation of cellular process	10826	394	302.44	1.30	+	3.86E-09
↳regulation of mRNA processing	149	43	4.16	10.33	+	3.57E-23
↳regulation of mRNA metabolic process	255	65	7.12	9.12	+	7.64E-34
alternative mRNA splicing, via spliceosome	18	7	.50	13.92	+	2.96E-02
↳mRNA splicing, via spliceosome	196	42	5.48	7.67	+	2.76E-18
↳mRNA processing	405	73	11.31	6.45	+	9.02E-30
↳RNA processing	752	87	21.01	4.14	+	2.03E-23
↳mRNA metabolic process	531	85	14.83	5.73	+	6.98E-32
↳RNA splicing, via transesterification reactions with bulged adenosine as nucleophile	196	42	5.48	7.67	+	2.76E-18
↳RNA splicing, via transesterification reactions	196	42	5.48	7.67	+	2.76E-18
↳RNA splicing	318	68	8.88	7.65	+	1.85E-31
3'-UTR-mediated mRNA destabilization	18	7	.50	13.92	+	2.96E-02
↳mRNA destabilization	32	13	.89	14.54	+	1.14E-06
↳RNA destabilization	35	13	.98	13.30	+	2.79E-06
↳regulation of RNA stability	109	27	3.05	8.87	+	3.92E-12
↳regulation of cellular catabolic process	698	65	19.50	3.33	+	2.91E-12
↳regulation of catabolic process	843	73	23.55	3.10	+	1.36E-12
↳posttranscriptional regulation of gene expression	427	81	11.93	6.79	+	7.28E-35
↳regulation of biological quality	3959	213	110.60	1.93	+	3.97E-18
↳positive regulation of nucleobase-containing compound metabolic process	1811	86	50.59	1.70	+	2.48E-02
↳positive regulation of cellular metabolic process	3270	142	91.35	1.55	+	1.11E-03
↳positive regulation of cellular process	5424	235	151.53	1.55	+	2.28E-09
↳positive regulation of metabolic process	3560	155	99.45	1.56	+	1.84E-04
↳positive regulation of nitrogen compound metabolic process	3086	139	86.21	1.61	+	1.47E-04
↳positive regulation of macromolecule metabolic process	3256	141	90.96	1.55	+	1.45E-03
↳positive regulation of cellular catabolic process	367	35	10.25	3.41	+	1.45E-05
↳positive regulation of catabolic process	436	40	12.18	3.28	+	2.79E-06
↳positive regulation of mRNA catabolic process	50	15	1.40	10.74	+	1.45E-06
↳positive regulation of mRNA metabolic process	93	24	2.60	9.24	+	7.87E-11

↳regulation of mRNA catabolic process	121	29	3.38	8.58	+	6.72E-13
↳regulation of mRNA stability	98	27	2.74	9.86	+	4.09E-13
↳negative regulation of translation	130	34	3.63	9.36	+	1.34E-16
↳negative regulation of cellular protein metabolic process	1004	71	28.05	2.53	+	5.74E-08
↳negative regulation of cellular metabolic process	2469	129	68.97	1.87	+	7.94E-08
↳negative regulation of metabolic process	2766	145	77.27	1.88	+	1.59E-09
↳negative regulation of biological process	5169	247	144.40	1.71	+	1.74E-15
↳negative regulation of cellular process	4632	217	129.40	1.68	+	1.15E-11
↳regulation of cellular protein metabolic process	2531	137	70.71	1.94	+	1.19E-09
↳regulation of protein metabolic process	2723	147	76.07	1.93	+	9.65E-11
↳negative regulation of protein metabolic process	1072	72	29.95	2.40	+	2.98E-07
↳negative regulation of nitrogen compound metabolic process	2272	118	63.47	1.86	+	9.41E-07
↳negative regulation of macromolecule metabolic process	2494	132	69.67	1.89	+	1.55E-08
↳negative regulation of cellular amide metabolic process	149	35	4.16	8.41	+	7.05E-16
↳regulation of cellular amide metabolic process	384	71	10.73	6.62	+	1.80E-29
↳regulation of translation	333	70	9.30	7.52	+	4.46E-32
↳regulation of cellular macromolecule biosynthetic process	3333	156	93.11	1.68	+	9.83E-07
↳regulation of macromolecule biosynthetic process	3419	159	95.51	1.66	+	1.05E-06
↳regulation of biosynthetic process	3644	167	101.80	1.64	+	7.38E-07
↳regulation of cellular biosynthetic process	3571	164	99.76	1.64	+	1.07E-06
↳negative regulation of macromolecule biosynthetic process	1414	71	39.50	1.80	+	2.83E-02
↳negative regulation of gene expression	1643	93	45.90	2.03	+	2.28E-06
positive regulation of gene silencing by miRNA	24	9	.67	13.42	+	1.46E-03
↳positive regulation of posttranscriptional gene silencing	25	9	.70	12.89	+	1.94E-03
↳regulation of posttranscriptional gene silencing	44	10	1.23	8.14	+	1.48E-02
↳regulation of gene silencing by miRNA	41	10	1.15	8.73	+	8.51E-03
↳regulation of gene silencing by RNA	44	10	1.23	8.14	+	1.48E-02
negative regulation of mRNA splicing, via spliceosome	24	9	.67	13.42	+	1.46E-03
↳negative regulation of RNA splicing	29	10	.81	12.34	+	5.68E-04
↳negative regulation of mRNA processing	32	10	.89	11.19	+	1.22E-03
↳negative regulation of mRNA metabolic process	82	21	2.29	9.17	+	4.26E-09
regulation of cytoplasmic translation	25	9	.70	12.89	+	1.94E-03
mRNA cis splicing, via spliceosome	28	10	.78	12.78	+	4.33E-04
tricarboxylic acid cycle	32	10	.89	11.19	+	1.22E-03
↳generation of precursor metabolites and energy	286	29	7.99	3.63	+	1.07E-04
translational initiation	55	17	1.54	11.06	+	5.83E-08
nuclear migration	31	9	.87	10.39	+	8.74E-03
↳nucleus localization	36	9	1.01	8.95	+	2.51E-02
↳organelle localization	474	46	13.24	3.47	+	2.12E-08
↳cellular localization	2053	166	57.35	2.89	+	4.85E-31
↳localization	4864	233	135.88	1.71	+	4.04E-14
↳intracellular transport	1182	116	33.02	3.51	+	1.11E-26
↳transport	3572	190	99.79	1.90	+	6.94E-15
↳establishment of localization	3711	197	103.67	1.90	+	1.73E-15
↳establishment of organelle localization	331	34	9.25	3.68	+	4.30E-06
positive regulation of telomere maintenance via telomere lengthening	35	10	.98	10.23	+	2.46E-03
↳positive regulation of telomere maintenance	50	11	1.40	7.88	+	5.92E-03

↳positive regulation of DNA metabolic process	212	21	5.92	3.55	+	1.55E-02
↳regulation of DNA metabolic process	368	28	10.28	2.72	+	4.07E-02
↳positive regulation of organelle organization	596	50	16.65	3.00	+	3.10E-07
↳positive regulation of cellular component organization	1254	86	35.03	2.45	+	9.87E-10
↳regulation of cellular component organization	2537	157	70.87	2.22	+	5.18E-17
↳regulation of organelle organization	1270	95	35.48	2.68	+	1.81E-13
↳regulation of telomere maintenance	81	15	2.26	6.63	+	4.20E-04
positive regulation of dendritic spine morphogenesis	29	8	.81	9.87	+	4.87E-02
↳positive regulation of dendritic spine development	65	14	1.82	7.71	+	2.26E-04
↳regulation of dendritic spine development	97	16	2.71	5.90	+	6.28E-04
↳regulation of dendrite development	194	21	5.42	3.87	+	4.18E-03
↳regulation of neuron projection development	609	43	17.01	2.53	+	9.43E-04
↳regulation of plasma membrane bounded cell projection organization	781	52	21.82	2.38	+	2.38E-04
↳regulation of cell projection organization	790	52	22.07	2.36	+	4.70E-04
↳regulation of neuron differentiation	776	48	21.68	2.21	+	7.99E-03
↳regulation of neurogenesis	953	58	26.62	2.18	+	7.84E-04
↳generation of neurons	1663	90	46.46	1.94	+	3.63E-05
↳neurogenesis	1771	93	49.48	1.88	+	9.34E-05
↳nervous system development	2266	114	63.30	1.80	+	1.27E-05
↳regulation of cell development	1092	66	30.51	2.16	+	1.21E-04
↳regulation of cell differentiation	1870	88	52.24	1.68	+	2.44E-02
↳regulation of developmental process	2684	118	74.98	1.57	+	9.98E-03
↳regulation of nervous system development	1070	61	29.89	2.04	+	3.77E-03
↳regulation of multicellular organismal process	3223	143	90.04	1.59	+	2.54E-04
↳positive regulation of dendrite development	110	16	3.07	5.21	+	2.92E-03
↳positive regulation of neuron projection development	369	30	10.31	2.91	+	5.25E-03
↳positive regulation of cell projection organization	474	36	13.24	2.72	+	1.87E-03
↳positive regulation of neuron differentiation	469	33	13.10	2.52	+	3.59E-02
↳positive regulation of neurogenesis	583	38	16.29	2.33	+	3.34E-02
↳positive regulation of cell development	665	42	18.58	2.26	+	2.36E-02
↳positive regulation of cell morphogenesis involved in differentiation	189	22	5.28	4.17	+	7.08E-04
↳regulation of cell morphogenesis involved in differentiation	348	34	9.72	3.50	+	1.41E-05
↳regulation of cell morphogenesis	537	47	15.00	3.13	+	3.29E-07
↳regulation of anatomical structure morphogenesis	1090	62	30.45	2.04	+	2.93E-03
↳regulation of dendritic spine morphogenesis	56	11	1.56	7.03	+	1.57E-02
↳regulation of postsynapse organization	131	16	3.66	4.37	+	2.35E-02
↳regulation of synapse organization	273	28	7.63	3.67	+	1.53E-04
↳regulation of synapse structure or activity	285	30	7.96	3.77	+	2.72E-05
positive regulation of mRNA splicing, via spliceosome	33	9	.92	9.76	+	1.36E-02
↳positive regulation of mRNA processing	44	11	1.23	8.95	+	1.97E-03
↳positive regulation of gene expression	1995	97	55.73	1.74	+	1.77E-03
↳positive regulation of RNA splicing	50	15	1.40	10.74	+	1.45E-06
positive regulation of translation	123	33	3.44	9.60	+	2.56E-16
↳positive regulation of biosynthetic process	1953	92	54.56	1.69	+	1.32E-02
↳positive regulation of cellular amide metabolic process	149	34	4.16	8.17	+	5.19E-15
↳positive regulation of cellular biosynthetic process	1912	91	53.41	1.70	+	1.03E-02
spliceosomal complex assembly	45	12	1.26	9.55	+	3.01E-04

↳ribonucleoprotein complex assembly	163	25	4.55	5.49	+	5.44E-07
↳cellular protein-containing complex assembly	653	47	18.24	2.58	+	1.47E-04
↳protein-containing complex assembly	1003	67	28.02	2.39	+	1.97E-06
↳protein-containing complex subunit organization	1144	77	31.96	2.41	+	4.86E-08
↳ribonucleoprotein complex biogenesis	392	33	10.95	3.01	+	6.78E-04
↳ribonucleoprotein complex subunit organization	170	26	4.75	5.47	+	2.38E-07
regulation of translational initiation	59	15	1.65	9.10	+	1.02E-05
mRNA stabilization	41	10	1.15	8.73	+	8.51E-03
↳negative regulation of mRNA catabolic process	51	11	1.42	7.72	+	7.03E-03
↳negative regulation of RNA catabolic process	61	13	1.70	7.63	+	7.92E-04
↳negative regulation of cellular catabolic process	241	27	6.73	4.01	+	5.05E-05
↳negative regulation of catabolic process	301	28	8.41	3.33	+	1.01E-03
↳RNA stabilization	48	11	1.34	8.20	+	4.17E-03
posttranscriptional gene silencing by RNA	47	11	1.31	8.38	+	3.47E-03
↳gene silencing by RNA	77	12	2.15	5.58	+	4.57E-02
↳posttranscriptional gene silencing	48	11	1.34	8.20	+	4.17E-03
Golgi to plasma membrane transport	52	11	1.45	7.57	+	8.31E-03
↳vesicle-mediated transport to the plasma membrane	85	15	2.37	6.32	+	7.35E-04
↳vesicle-mediated transport	1271	81	35.51	2.28	+	1.72E-07
↳post-Golgi vesicle-mediated transport	88	15	2.46	6.10	+	1.10E-03
↳Golgi vesicle transport	250	35	6.98	5.01	+	8.73E-10
cortical cytoskeleton organization	59	12	1.65	7.28	+	3.85E-03
↳cytoskeleton organization	1070	75	29.89	2.51	+	1.60E-08
regulation of actin filament depolymerization	51	10	1.42	7.02	+	4.71E-02
↳regulation of actin polymerization or depolymerization	185	22	5.17	4.26	+	5.03E-04
↳regulation of actin filament-based process	409	31	11.43	2.71	+	1.36E-02
↳regulation of cytoskeleton organization	559	51	15.62	3.27	+	1.12E-08
↳regulation of supramolecular fiber organization	368	34	10.28	3.31	+	5.19E-05
↳regulation of actin filament length	188	22	5.25	4.19	+	6.50E-04
↳regulation of cellular component size	407	39	11.37	3.43	+	1.46E-06
↳regulation of anatomical structure size	571	47	15.95	2.95	+	2.21E-06
↳regulation of protein depolymerization	89	13	2.49	5.23	+	3.53E-02
↳regulation of protein-containing complex disassembly	121	16	3.38	4.73	+	9.18E-03
positive regulation of axon extension	57	11	1.59	6.91	+	1.83E-02
↳positive regulation of axonogenesis	105	14	2.93	4.77	+	3.95E-02
↳regulation of axonogenesis	207	20	5.78	3.46	+	3.86E-02
↳positive regulation of developmental growth	216	21	6.03	3.48	+	2.04E-02
↳positive regulation of growth	299	26	8.35	3.11	+	9.35E-03
↳positive regulation of cell growth	187	21	5.22	4.02	+	2.40E-03
↳regulation of axon extension	113	15	3.16	4.75	+	1.90E-02
negative regulation of protein polymerization	73	14	2.04	6.86	+	8.03E-04
↳negative regulation of protein-containing complex assembly	136	18	3.80	4.74	+	1.94E-03
↳regulation of protein-containing complex assembly	434	43	12.12	3.55	+	6.07E-08
↳regulation of cellular component biogenesis	964	70	26.93	2.60	+	1.95E-08
↳negative regulation of cellular component organization	734	47	20.51	2.29	+	3.87E-03
↳regulation of protein polymerization	224	31	6.26	4.95	+	3.01E-08
axo-dendritic transport	73	13	2.04	6.37	+	4.89E-03

↳transport along microtubule	155	19	4.33	4.39	+	2.63E-03
↳microtubule-based transport	184	19	5.14	3.70	+	2.76E-02
↳microtubule-based movement	267	24	7.46	3.22	+	1.42E-02
↳microtubule-based process	666	45	18.61	2.42	+	1.80E-03
↳cytoskeleton-dependent intracellular transport	186	20	5.20	3.85	+	8.59E-03
mRNA transport	107	18	2.99	6.02	+	7.32E-05
↳RNA transport	150	21	4.19	5.01	+	7.92E-05
↳nucleic acid transport	150	21	4.19	5.01	+	7.92E-05
↳nucleobase-containing compound transport	183	22	5.11	4.30	+	4.22E-04
↳organic substance transport	1856	118	51.85	2.28	+	1.61E-12
↳nitrogen compound transport	1550	112	43.30	2.59	+	2.62E-15
↳establishment of RNA localization	152	21	4.25	4.95	+	9.76E-05
↳RNA localization	170	24	4.75	5.05	+	5.93E-06
↳macromolecule localization	2266	153	63.30	2.42	+	3.83E-20
endoplasmic reticulum to Golgi vesicle-mediated transport	105	17	2.93	5.80	+	3.13E-04
positive regulation of viral process	82	13	2.29	5.67	+	1.57E-02
positive regulation of DNA biosynthetic process	76	12	2.12	5.65	+	4.06E-02
protein folding	146	22	4.08	5.39	+	1.03E-05
protein stabilization	174	26	4.86	5.35	+	3.77E-07
↳regulation of protein stability	278	30	7.77	3.86	+	1.60E-05
lysosomal transport	94	14	2.63	5.33	+	1.23E-02
↳vacuolar transport	131	16	3.66	4.37	+	2.35E-02
Golgi organization	111	16	3.10	5.16	+	3.26E-03
↳endomembrane system organization	367	41	10.25	4.00	+	5.82E-09
protein import into nucleus	98	14	2.74	5.11	+	1.91E-02
↳import into nucleus	102	14	2.85	4.91	+	2.92E-02
↳nucleocytoplasmic transport	203	21	5.67	3.70	+	8.21E-03
↳nuclear transport	203	21	5.67	3.70	+	8.21E-03
↳establishment of protein localization to organelle	302	38	8.44	4.50	+	1.54E-09
↳protein localization to organelle	622	65	17.38	3.74	+	1.53E-14
↳cellular protein localization	1423	114	39.75	2.87	+	3.42E-19
↳cellular macromolecule localization	1430	114	39.95	2.85	+	4.96E-19
↳protein localization	1963	136	54.84	2.48	+	4.64E-18
↳establishment of protein localization	1330	105	37.16	2.83	+	6.81E-17
↳protein transport	1246	98	34.81	2.82	+	2.15E-15
↳peptide transport	1276	98	35.65	2.75	+	9.78E-15
↳amide transport	1301	99	36.35	2.72	+	1.14E-14
↳protein localization to nucleus	172	19	4.81	3.95	+	1.11E-02
↳intracellular protein transport	765	70	21.37	3.28	+	4.42E-13
maintenance of location in cell	103	14	2.88	4.87	+	3.23E-02
cytosolic transport	132	17	3.69	4.61	+	5.92E-03
regulation of microtubule cytoskeleton organization	203	26	5.67	4.58	+	7.62E-06
↳regulation of microtubule-based process	241	28	6.73	4.16	+	1.26E-05
regulation of nucleocytoplasmic transport	125	16	3.49	4.58	+	1.35E-02
↳regulation of intracellular transport	352	33	9.83	3.36	+	6.28E-05
↳regulation of transport	1972	100	55.09	1.82	+	1.33E-04
regulation of actin filament polymerization	168	21	4.69	4.47	+	4.66E-04
negative regulation of supramolecular fiber organization	154	19	4.30	4.42	+	2.41E-03

establishment of vesicle localization	142	17	3.97	4.29	+	1.48E-02
↳establishment of localization in cell	353	29	9.86	2.94	+	6.64E-03
↳vesicle localization	154	18	4.30	4.18	+	1.01E-02
negative regulation of cytoskeleton organization	161	19	4.50	4.22	+	4.47E-03
ATP metabolic process	170	19	4.75	4.00	+	9.44E-03
establishment of protein localization to membrane	180	19	5.03	3.78	+	2.05E-02
endosomal transport	203	20	5.67	3.53	+	2.94E-02
vesicle organization	234	23	6.54	3.52	+	5.71E-03
protein targeting	217	21	6.06	3.46	+	2.18E-02
establishment or maintenance of cell polarity	207	20	5.78	3.46	+	3.86E-02
positive regulation of protein-containing complex assembly	221	21	6.17	3.40	+	2.84E-02
negative regulation of neuron death	247	23	6.90	3.33	+	1.34E-02
↳negative regulation of cell death	1041	59	29.08	2.03	+	7.31E-03
↳regulation of cell death	1672	84	46.71	1.80	+	3.17E-03
regulation of muscle system process	242	22	6.76	3.25	+	3.24E-02
actin cytoskeleton organization	500	38	13.97	2.72	+	8.43E-04
↳actin filament-based process	555	41	15.50	2.64	+	5.37E-04
cell part morphogenesis	507	37	14.16	2.61	+	3.17E-03
cellular response to nitrogen compound	512	35	14.30	2.45	+	2.84E-02
↳response to nitrogen compound	851	55	23.77	2.31	+	2.51E-04
↳response to chemical	3484	144	97.33	1.48	+	1.35E-02
↳cellular response to chemical stimulus	2359	106	65.90	1.61	+	1.76E-02
neuron projection development	691	47	19.30	2.43	+	8.03E-04
↳neuron development	843	49	23.55	2.08	+	3.73E-02
response to organonitrogen compound	745	49	20.81	2.35	+	8.58E-04
↳response to organic substance	2488	115	69.51	1.65	+	1.09E-03
cellular macromolecule catabolic process	762	48	21.29	2.25	+	3.99E-03
↳catabolic process	1730	84	48.33	1.74	+	1.51E-02
↳macromolecule catabolic process	858	51	23.97	2.13	+	9.68E-03
↳organic substance catabolic process	1433	76	40.03	1.90	+	1.60E-03
regulation of protein transport	739	44	20.64	2.13	+	4.99E-02
positive regulation of transport	1099	63	30.70	2.05	+	1.93E-03
cellular response to stress	1446	74	40.40	1.83	+	9.84E-03
Unclassified	1901	14	53.11	.26	-	0.00E00
sensory perception	1642	7	45.87	.15	-	7.96E-09
G protein-coupled receptor signaling pathway	1851	7	51.71	.14	-	3.75E-11

About | Release Information | Contact Us | System Requirements | Privacy Policy | Disclaimer
© Copyright 2020 Paul Thomas All Rights Reserved.

PANTHER - Compare lists to reference list

LOGIN REGISTER CONTACT US

Home | About | PANTHER Data | PANTHER Tools | PANTHER Services | Workspace | Downloads | Help/Tutorial

PANTHER 15.0 released!

Analysis Summary: Please report in publication

Analysis Type: PANTHER Overrepresentation Test (Released 20200407)

Annotation Version and Release Date: GO Ontology database Released 2020-02-21

Analyzed List: upload_1 (Mus musculus) Change

Reference List: Mus musculus (all genes in database) Change

Annotation Data Set: GO molecular function complete

Test Type: (•) Fisher's Exact ( ) Binomial

Correction: ( ) Calculate False Discovery Rate (•) Use the Bonferroni correction for multiple testing ( ) No correction

Results

	Reference list	upload_1
Uniquely Mapped IDS:	22265 out of 22265	622 out of 622
Unmapped IDs:	0	0
Multiple mapping information:	0	0

Bonferroni count: 2756

Export Table | XML with user input ids | JSON with user input ids

Displaying only results for Bonferroni-corrected for P < 0.05, click here to display all results

	Mus musculus (REF)	upload_1 (▽ Hierarchy NEW!)				
GO molecular function complete	#	#	expected	Fold Enrichment	+/-	P value
N6-methyladenosine-containing RNA binding	8	6	.22	26.85	+	2.70E-03
↳RNA binding	1086	202	30.34	6.66	+	9.99E-97
↳nucleic acid binding	3161	236	88.31	2.67	+	2.21E-43
↳organic cyclic compound binding	5175	346	144.57	2.39	+	6.07E-62
↳binding	13351	568	372.98	1.52	+	1.11E-64
↳heterocyclic compound binding	5072	344	141.69	2.43	+	7.35E-63
RNA stem-loop binding	14	8	.39	20.45	+	1.79E-04
sequence-specific mRNA binding	13	6	.36	16.52	+	2.17E-02
↳mRNA binding	272	79	7.60	10.40	+	5.27E-46
aminoacyl-tRNA ligase activity	41	18	1.15	15.72	+	3.25E-11
↳ligase activity, forming carbon-oxygen bonds	41	18	1.15	15.72	+	3.25E-11
↳ligase activity	146	25	4.08	6.13	+	2.04E-08
↳catalytic activity	5659	230	158.09	1.45	+	1.02E-06
↳catalytic activity, acting on a tRNA	113	18	3.16	5.70	+	4.82E-05
↳catalytic activity, acting on RNA	337	41	9.41	4.35	+	1.42E-10
RNA cap binding	17	7	.47	14.74	+	6.75E-03
mRNA 5'-UTR binding	24	9	.67	13.42	+	4.53E-04
mRNA 3'-UTR AU-rich region binding	27	10	.75	13.26	+	1.01E-04
↳mRNA 3'-UTR binding	87	29	2.43	11.93	+	1.13E-16
↳AU-rich element binding	28	10	.78	12.78	+	1.34E-04
RNA helicase activity	53	17	1.48	11.48	+	1.10E-08

↳helicase activity	148	21	4.13	5.08	+	1.98E-05
↳ATPase activity, coupled	279	28	7.79	3.59	+	7.25E-05
↳ATPase activity	412	37	11.51	3.21	+	6.91E-06
↳nucleoside-triphosphatase activity	748	61	20.90	2.92	+	1.79E-09
↳pyrophosphatase activity	801	66	22.38	2.95	+	1.20E-10
↳hydrolase activity, acting on acid anhydrides, in phosphorus-containing anhydrides	804	66	22.46	2.94	+	1.41E-10
↳hydrolase activity, acting on acid anhydrides	804	66	22.46	2.94	+	1.41E-10
↳hydrolase activity	2449	113	68.42	1.65	+	5.50E-04
translation repressor activity	22	7	.61	11.39	+	2.71E-02
↳translation regulator activity	126	40	3.52	11.36	+	3.59E-23
translation initiation factor activity	48	15	1.34	11.19	+	2.78E-07
↳translation factor activity, RNA binding	77	25	2.15	11.62	+	7.39E-14
↳translation regulator activity, nucleic acid binding	95	29	2.65	10.93	+	8.33E-16
pre-mRNA binding	33	10	.92	10.85	+	4.81E-04
ribosome binding	64	19	1.79	10.63	+	1.89E-09
↳ribonucleoprotein complex binding	146	30	4.08	7.36	+	2.28E-12
↳protein-containing complex binding	1451	119	40.54	2.94	+	1.47E-21
Ran GTPase binding	34	10	.95	10.53	+	6.08E-04
↳Ras GTPase binding	399	38	11.15	3.41	+	9.18E-07
↳small GTPase binding	416	40	11.62	3.44	+	2.39E-07
↳GTPase binding	515	51	14.39	3.54	+	1.94E-10
↳enzyme binding	2331	176	65.12	2.70	+	2.47E-30
↳protein binding	9112	438	254.55	1.72	+	4.29E-45
poly-purine tract binding	32	9	.89	10.07	+	3.39E-03
↳single-stranded RNA binding	89	22	2.49	8.85	+	6.75E-10
double-stranded RNA binding	82	23	2.29	10.04	+	1.96E-11
miRNA binding	30	8	.84	9.55	+	1.87E-02
↳regulatory RNA binding	43	9	1.20	7.49	+	2.73E-02
ADP binding	40	10	1.12	8.95	+	2.17E-03
↳adenyl ribonucleotide binding	1453	115	40.59	2.83	+	1.65E-19
↳adenyl nucleotide binding	1464	117	40.90	2.86	+	3.03E-20
↳purine nucleotide binding	1798	142	50.23	2.83	+	4.26E-25
↳nucleotide binding	2030	155	56.71	2.73	+	2.11E-26
↳small molecule binding	2421	173	67.63	2.56	+	6.35E-27
↳nucleoside phosphate binding	2030	155	56.71	2.73	+	2.11E-26
↳purine ribonucleotide binding	1786	140	49.89	2.81	+	2.31E-24
↳ribonucleotide binding	1802	141	50.34	2.80	+	1.68E-24
↳carbohydrate derivative binding	2123	148	59.31	2.50	+	4.07E-21
↳anion binding	2703	179	75.51	2.37	+	2.94E-24
↳ion binding	5485	244	153.23	1.59	+	7.14E-12
structural constituent of cytoskeleton	64	15	1.79	8.39	+	8.21E-06
↳structural molecule activity	576	53	16.09	3.29	+	8.99E-10
Hsp90 protein binding	47	10	1.31	7.62	+	7.70E-03
↳heat shock protein binding	140	23	3.91	5.88	+	2.94E-07
unfolded protein binding	92	19	2.57	7.39	+	4.23E-07
rRNA binding	70	13	1.96	6.65	+	9.92E-04
single-stranded DNA binding	111	16	3.10	5.16	+	1.01E-03
ubiquitin protein ligase binding	308	35	8.60	4.07	+	5.93E-08
↳ubiquitin-like protein ligase binding	323	36	9.02	3.99	+	5.04E-08
protein C-terminus binding	231	25	6.45	3.87	+	1.07E-04
Rho GTPase binding	162	17	4.53	3.76	+	2.30E-02

actin filament binding	201	21	5.62	3.74	+	2.20E-03
↳actin binding	426	37	11.90	3.11	+	1.58E-05
↳cytoskeletal protein binding	976	81	27.27	2.97	+	7.56E-14
microtubule binding	247	25	6.90	3.62	+	3.48E-04
↳tubulin binding	346	32	9.67	3.31	+	4.43E-05
ATP binding	1390	113	38.83	2.91	+	5.67E-20
↳drug binding	1644	128	45.93	2.79	+	1.34E-21
↳purine ribonucleoside triphosphate binding	1713	138	47.85	2.88	+	4.86E-25
GTP binding	355	27	9.92	2.72	+	1.87E-02
↳purine ribonucleoside binding	362	28	10.11	2.77	+	9.43E-03
↳ribonucleoside binding	365	29	10.20	2.84	+	3.85E-03
↳nucleoside binding	375	29	10.48	2.77	+	6.34E-03
↳purine nucleoside binding	366	28	10.22	2.74	+	1.15E-02
↳guanyl ribonucleotide binding	379	29	10.59	2.74	+	7.71E-03
↳guanyl nucleotide binding	379	29	10.59	2.74	+	7.71E-03
protein kinase binding	727	54	20.31	2.66	+	1.02E-06
↳kinase binding	813	58	22.71	2.55	+	9.94E-07
protein domain specific binding	786	55	21.96	2.50	+	5.38E-06
identical protein binding	1983	120	55.40	2.17	+	1.17E-11
Unclassified	2116	17	59.11	.29	-	0.00E00
signaling receptor activity	2339	2	65.34	.03	-	3.30E-23
↳molecular transducer activity	2344	2	65.48	.03	-	3.35E-23

About | Release Information | Contact Us | System Requirements | Privacy Policy | Disclaimer
© Copyright 2020 Paul Thomas All Rights Reserved.

PANTHER 15.0 released!

Analysis Summary: Please report in publication

Analysis Type: PANTHER Overrepresentation Test (Released 20200407)

Annotation Version and Release Date: GO Ontology database Released 2020-02-21

Analyzed List: upload_1 (Mus musculus) Change

Reference List: Mus musculus (all genes in database) Change

Annotation Data Set: GO cellular component complete

Test Type: (•) Fisher's Exact ( ) Binomial

Correction: ( ) Calculate False Discovery Rate (•) Use the Bonferroni correction for multiple testing ( ) No correction

Results

	Reference list	upload_1
Uniquely Mapped IDS:	22265 out of 22265	622 out of 622
Unmapped IDs:	0	0
Multiple mapping information:	0	0

Bonferroni count: 1452

Export Table | XML with user input ids | JSON with user input ids

Displaying only results for Bonferroni-corrected for P < 0.05, click here to display all results

	Mus musculus (REF)	upload_1 (Hierarchy NEW!)				
GO cellular component complete	#	#	expected	Fold Enrichment	+/-	P value
dendritic filopodium	5	5	.14	35.80	+	4.77E-03
↳dendrite	705	68	19.70	3.45	+	1.77E-14
↳neuron projection	1516	112	42.35	2.64	+	6.30E-17
↳plasma membrane bounded cell projection	2281	136	63.72	2.13	+	1.30E-13
↳cell projection	2499	151	69.81	2.16	+	4.02E-16
↳cellular anatomical entity	18638	604	520.68	1.16	+	3.98E-23
↳dendritic tree	708	68	19.78	3.44	+	2.17E-14
↳somatodendritic compartment	1028	92	28.72	3.20	+	1.84E-18

↳filopodium	93	12	2.60	4.62	+	4.14E-02
↳actin-based cell projection	216	22	6.03	3.65	+	9.42E-04
eukaryotic translation initiation factor 3 complex, eIF3m	8	6	.22	26.85	+	1.42E-03
↳eukaryotic translation initiation factor 3 complex	15	11	.42	26.25	+	4.24E-08
↳cytoplasm	10945	546	305.76	1.79	+	9.57E-87
↳intracellular	13691	601	382.47	1.57	+	3.23E-92
↳protein-containing complex	5309	340	148.31	2.29	+	9.02E-56
chaperonin-containing T-complex	10	7	.28	25.06	+	2.36E-04
↳chaperone complex	22	12	.61	19.52	+	6.74E-08
↳cytosol	3533	271	98.70	2.75	+	7.79E-55
aminoacyl-tRNA synthetase multienzyme complex	12	8	.34	23.86	+	3.90E-05
zona pellucida receptor complex	13	7	.36	19.27	+	8.76E-04
messenger ribonucleoprotein complex	13	6	.36	16.52	+	1.15E-02
↳ribonucleoprotein complex	703	103	19.64	5.24	+	2.66E-37
postsynaptic cytoskeleton	19	8	.53	15.07	+	5.81E-04
↳postsynapse	727	87	20.31	4.28	+	3.76E-25
↳synapse	1435	143	40.09	3.57	+	1.02E-35
↳cell junction	2028	162	56.65	2.86	+	2.60E-30
↳cytoskeleton	2135	131	59.64	2.20	+	8.51E-14
↳intracellular non-membrane-bounded organelle	4162	252	116.27	2.17	+	4.42E-32
↳intracellular organelle	11954	540	333.95	1.62	+	5.06E-65
↳organelle	12303	543	343.70	1.58	+	1.01E-61
↳non-membrane-bounded organelle	4181	253	116.80	2.17	+	3.09E-32
cytoplasmic stress granule	66	26	1.84	14.10	+	1.79E-16
↳cytoplasmic ribonucleoprotein granule	206	46	5.75	7.99	+	9.84E-22
↳ribonucleoprotein granule	217	50	6.06	8.25	+	2.39E-24
↳supramolecular complex	1197	122	33.44	3.65	+	1.47E-30
postsynaptic cytosol	26	9	.73	12.39	+	4.16E-04
↳region of cytosol	40	10	1.12	8.95	+	1.14E-03
endoplasmic reticulum exit site	21	7	.59	11.93	+	1.11E-02
↳endoplasmic reticulum	1639	83	45.79	1.81	+	4.19E-04
↳endomembrane system	3884	195	108.50	1.80	+	1.56E-13
↳intracellular membrane-bounded organelle	10291	473	287.49	1.65	+	2.74E-47
↳membrane-bounded organelle	11048	491	308.64	1.59	+	8.66E-47
myelin sheath	213	66	5.95	11.09	+	1.23E-39
proteasome regulatory particle	24	7	.67	10.44	+	2.29E-02
↳proteasome accessory complex	26	7	.73	9.64	+	3.55E-02
↳proteasome complex	66	11	1.84	5.97	+	1.04E-02

↳endopeptidase complex	67	11	1.87	5.88	+	1.19E-02
↳peptidase complex	93	12	2.60	4.62	+	4.14E-02
↳catalytic complex	1341	74	37.46	1.98	+	9.71E-05
polysome	73	17	2.04	8.34	+	4.17E-07
smooth endoplasmic reticulum	36	8	1.01	7.95	+	3.09E-02
catalytic step 2 spliceosome	83	18	2.32	7.76	+	3.41E-07
↳spliceosomal complex	197	38	5.50	6.90	+	1.04E-15
↳nucleus	6824	330	190.64	1.73	+	6.11E-27
mitochondrial nucleoid	47	10	1.31	7.62	+	4.06E-03
↳mitochondrial matrix	272	33	7.60	4.34	+	2.45E-08
↳intracellular organelle lumen	4311	242	120.43	2.01	+	2.85E-25
↳organelle lumen	4312	242	120.46	2.01	+	2.88E-25
↳membrane-enclosed lumen	4312	242	120.46	2.01	+	2.88E-25
↳mitochondrion	1803	124	50.37	2.46	+	1.31E-16
↳nucleoid	47	10	1.31	7.62	+	4.06E-03
intercalated disc	62	13	1.73	7.51	+	1.53E-04
↳cell-cell contact zone	85	13	2.37	5.47	+	3.66E-03
vesicle coat	53	10	1.48	6.75	+	1.04E-02
↳coated vesicle membrane	76	12	2.12	5.65	+	6.62E-03
↳vesicle	2039	94	56.96	1.65	+	3.55E-03
↳organelle membrane	2118	114	59.17	1.93	+	5.67E-08
↳whole membrane	1152	61	32.18	1.90	+	6.20E-03
↳coated vesicle	188	18	5.25	3.43	+	2.11E-02
↳bounding membrane of organelle	1098	56	30.67	1.83	+	3.67E-02
↳membrane coat	95	13	2.65	4.90	+	1.09E-02
↳coated membrane	95	13	2.65	4.90	+	1.09E-02
U2-type spliceosomal complex	87	16	2.43	6.58	+	2.67E-05
small nuclear ribonucleoprotein complex	60	10	1.68	5.97	+	2.71E-02
↳Sm-like protein family complex	71	12	1.98	6.05	+	3.53E-03
P-body	72	12	2.01	5.97	+	4.02E-03
Golgi-associated vesicle membrane	62	10	1.73	5.77	+	3.49E-02
small ribosomal subunit	78	12	2.18	5.51	+	8.41E-03
↳ribosomal subunit	202	20	5.64	3.54	+	4.49E-03
↳ribosome	234	27	6.54	4.13	+	4.64E-06
nuclear matrix	88	13	2.46	5.29	+	5.16E-03
↳nuclear periphery	113	17	3.16	5.39	+	1.33E-04
↳nuclear lumen	3899	207	108.92	1.90	+	1.57E-17
growth cone	205	30	5.73	5.24	+	3.11E-09

↳site of polarized growth	213	31	5.95	5.21	+	1.51E-09
↳distal axon	379	37	10.59	3.49	+	4.41E-07
↳axon	715	66	19.97	3.30	+	4.11E-13
dendritic spine	193	25	5.39	4.64	+	2.13E-06
↳neuron spine	199	26	5.56	4.68	+	8.48E-07
postsynaptic density	396	49	11.06	4.43	+	1.27E-13
↳asymmetric synapse	400	50	11.17	4.47	+	4.16E-14
↳neuron to neuron synapse	427	52	11.93	4.36	+	2.54E-14
↳postsynaptic specialization	435	50	12.15	4.11	+	9.00E-13
nuclear speck	315	37	8.80	4.20	+	3.40E-09
↳nuclear body	682	55	19.05	2.89	+	2.06E-08
↳nucleoplasm	3324	181	92.86	1.95	+	1.25E-15
glutamatergic synapse	508	57	14.19	4.02	+	2.00E-14
perikaryon	138	15	3.86	3.89	+	2.80E-02
↳neuronal cell body	710	68	19.83	3.43	+	2.48E-14
↳cell body	801	76	22.38	3.40	+	4.27E-16
lamellipodium	166	17	4.64	3.67	+	1.63E-02
↳cell leading edge	389	37	10.87	3.40	+	8.58E-07
perinuclear region of cytoplasm	657	67	18.35	3.65	+	2.28E-15
microtubule	420	42	11.73	3.58	+	1.34E-08
↳microtubule cytoskeleton	1173	74	32.77	2.26	+	3.70E-07
↳polymeric cytoskeletal fiber	682	61	19.05	3.20	+	2.23E-11
↳supramolecular fiber	902	76	25.20	3.02	+	1.99E-13
↳supramolecular polymer	909	76	25.39	2.99	+	2.94E-13
cell cortex	316	31	8.83	3.51	+	1.12E-05
mitochondrial protein complex	260	23	7.26	3.17	+	4.87E-03
nuclear envelope	423	34	11.82	2.88	+	1.95E-04
↳organelle envelope	1069	71	29.86	2.38	+	9.96E-08
↳envelope	1070	71	29.89	2.38	+	1.03E-07
presynapse	590	47	16.48	2.85	+	9.79E-07
actin cytoskeleton	497	38	13.88	2.74	+	1.19E-04
mitochondrial membrane	617	38	17.24	2.20	+	1.90E-02
nucleolus	808	46	22.57	2.04	+	1.84E-02
plasma membrane region	1218	63	34.03	1.85	+	7.52E-03
Golgi apparatus	1419	73	39.64	1.84	+	1.38E-03
extracellular space	1951	21	54.50	.39	-	2.98E-04
↳extracellular region	2797	42	78.14	.54	-	6.46E-03
integral component of plasma membrane	1499	12	41.88	.29	-	1.01E-04

↳intrinsic component of plasma membrane	1576	13	44.03	.30	-	8.06E-05
↳intrinsic component of membrane	6027	59	168.37	.35	-	1.03E-23
↳integral component of membrane	5854	55	163.54	.34	-	3.40E-24
Unclassified	1476	10	41.23	.24	-	0.00E00

About | Release Information | Contact Us | System Requirements | Privacy Policy | Disclaimer

© Copyright 2020 Paul Thomas All Rights Reserved.

LOGIN REGISTER CONTACT US

Home | About | PANTHER Data | PANTHER Tools | PANTHER Services | Workspace | Downloads | Help/Tutorial

PANTHER 15.0 released!

Analysis Summary: Please report in publication

Analysis Type: PANTHER Overrepresentation Test (Released 20200407)

Annotation Version and Release Date: GO Ontology database Released 2020-02-21

Analyzed List: upload_1 (Mus musculus) Change

Reference List: Mus musculus (all genes in database) Change

Annotation Data Set: GO biological process complete

Test Type: Fisher's Exact Binomial

Correction: Calculate False Discovery Rate Use the Bonferroni correction for multiple testing No correction

Results

	Reference list	upload_1
Uniquely Mapped IDS:	22265 out of 22265	1344 out of 1344
Unmapped IDs:	0	0
Multiple mapping information:	0	0

Bonferroni count: 8894

Export Table XML with user input ids JSON with user input ids

Displaying only results for Bonferroni-corrected for P < 0.05, click here to display all results

	Mus musculus (REF)	upload 1 (Hierarchy NEW!)				
GO biological process complete	#	#	expected	Fold Enrichment	+/-	P value
fatty acid beta-oxidation using acyl-CoA dehydrogenase	10	9	.60	14.91	+	2.99E-03
↳fatty acid beta-oxidation	49	25	2.96	8.45	+	1.29E-09
↳fatty acid oxidation	69	28	4.17	6.72	+	3.89E-09
↳fatty acid metabolic process	309	67	18.65	3.59	+	5.29E-13
↳monocarboxylic acid metabolic process	467	107	28.19	3.80	+	3.66E-24
↳carboxylic acid metabolic process	761	198	45.94	4.31	+	1.12E-55
↳oxoacid metabolic process	803	200	48.47	4.13	+	1.08E-53
↳organic acid metabolic process	829	203	50.04	4.06	+	1.44E-53
↳cellular metabolic process	6331	729	382.16	1.91	+	8.44E-78
↳metabolic process	7222	788	435.95	1.81	+	4.41E-77
↳cellular process	14059	1142	848.65	1.35	+	4.52E-63
↳small molecule metabolic process	1432	263	86.44	3.04	+	1.26E-49
↳organic substance metabolic process	6701	748	404.50	1.85	+	6.13E-75
↳cellular lipid metabolic process	801	91	48.35	1.88	+	5.09E-04
↳lipid metabolic process	1063	112	64.17	1.75	+	5.95E-04
↳primary metabolic process	6269	699	378.42	1.85	+	9.74E-67
↳lipid oxidation	75	28	4.53	6.18	+	1.98E-08
↳lipid modification	182	35	10.99	3.19	+	2.11E-04

↳oxidation-reduction process	787	136	47.51	2.86	+	5.24E-21
↳fatty acid catabolic process	73	28	4.41	6.35	+	1.17E-08
↳cellular lipid catabolic process	164	32	9.90	3.23	+	6.20E-04
↳cellular catabolic process	1492	215	90.06	2.39	+	1.79E-25
↳catabolic process	1730	248	104.43	2.37	+	5.42E-30
↳lipid catabolic process	259	44	15.63	2.81	+	9.62E-05
↳organic substance catabolic process	1433	222	86.50	2.57	+	1.49E-30
↳monocarboxylic acid catabolic process	93	35	5.61	6.23	+	2.95E-11
↳carboxylic acid catabolic process	194	64	11.71	5.47	+	3.51E-20
↳organic acid catabolic process	194	64	11.71	5.47	+	3.51E-20
↳small molecule catabolic process	290	83	17.51	4.74	+	1.81E-23
positive regulation of establishment of protein localization to telomere	10	8	.60	13.25	+	2.52E-02
↳positive regulation of establishment of protein localization	462	71	27.89	2.55	+	2.88E-07
↳positive regulation of biological process	6100	522	368.22	1.42	+	1.25E-14
↳regulation of biological process	11534	822	696.24	1.18	+	2.12E-07
↳biological regulation	12152	877	733.54	1.20	+	1.16E-10
↳regulation of establishment of protein localization	771	105	46.54	2.26	+	3.15E-09
↳regulation of protein localization	1075	147	64.89	2.27	+	3.34E-14
↳regulation of localization	2886	290	174.21	1.66	+	3.26E-13
↳regulation of cellular protein localization	560	93	33.80	2.75	+	2.18E-12
↳regulation of cellular localization	837	118	50.52	2.34	+	1.55E-11
↳positive regulation of cellular protein localization	308	55	18.59	2.96	+	2.75E-07
↳regulation of establishment of protein localization to telomere	11	8	.66	12.05	+	4.13E-02
viral translation	14	10	.85	11.83	+	2.89E-03
↳viral gene expression	20	11	1.21	9.11	+	5.11E-03
↳viral process	131	29	7.91	3.67	+	2.41E-04
↳symbiotic process	228	46	13.76	3.34	+	3.44E-07
↳interspecies interaction between organisms	1474	135	88.98	1.52	+	4.01E-02
tricarboxylic acid metabolic process	15	10	.91	11.04	+	4.57E-03
alternative mRNA splicing, via spliceosome	18	11	1.09	10.12	+	2.32E-03
↳mRNA splicing, via spliceosome	196	50	11.83	4.23	+	2.28E-11
↳mRNA processing	405	104	24.45	4.25	+	7.65E-27
↳RNA processing	752	139	45.39	3.06	+	4.66E-24
↳RNA metabolic process	1232	197	74.37	2.65	+	3.29E-28
↳nucleic acid metabolic process	1767	221	106.66	2.07	+	7.36E-19
↳macromolecule metabolic process	5056	530	305.20	1.74	+	3.40E-36
↳nucleobase-containing compound metabolic process	2191	308	132.26	2.33	+	1.98E-37
↳cellular nitrogen compound metabolic process	2789	436	168.35	2.59	+	1.66E-70
↳nitrogen compound metabolic process	5718	640	345.16	1.85	+	1.26E-58
↳organic cyclic compound metabolic process	2616	359	157.91	2.27	+	4.87E-43
↳heterocycle metabolic process	2328	332	140.53	2.36	+	2.59E-42
↳cellular aromatic compound metabolic process	2402	337	144.99	2.32	+	1.24E-41
↳gene expression	1616	277	97.55	2.84	+	2.05E-47
↳mRNA metabolic process	531	130	32.05	4.06	+	1.33E-32
↳RNA splicing, via transesterification reactions with bulged adenosine as nucleophile	196	50	11.83	4.23	+	2.28E-11
↳RNA splicing, via transesterification reactions	196	50	11.83	4.23	+	2.28E-11
↳RNA splicing	318	87	19.20	4.53	+	1.41E-23

acyl-CoA biosynthetic process	20	12	1.21	9.94	+	7.64E-04
↳purine ribonucleotide biosynthetic process	115	27	6.94	3.89	+	2.54E-04
↳purine ribonucleotide metabolic process	256	68	15.45	4.40	+	3.24E-17
↳ribonucleotide metabolic process	266	69	16.06	4.30	+	4.98E-17
↳nucleotide metabolic process	352	79	21.25	3.72	+	1.36E-16
↳nucleoside phosphate metabolic process	362	79	21.85	3.62	+	5.77E-16
↳phosphate-containing compound metabolic process	1670	163	100.81	1.62	+	7.63E-05
↳phosphorus metabolic process	1692	164	102.14	1.61	+	9.54E-05
↳nucleobase-containing small molecule metabolic process	426	91	25.71	3.54	+	2.82E-18
↳organophosphate metabolic process	710	104	42.86	2.43	+	8.04E-11
↳ribose phosphate metabolic process	277	71	16.72	4.25	+	2.36E-17
↳carbohydrate derivative metabolic process	794	102	47.93	2.13	+	1.62E-07
↳purine nucleotide metabolic process	273	71	16.48	4.31	+	1.17E-17
↳purine-containing compound metabolic process	313	76	18.89	4.02	+	1.42E-17
↳organonitrogen compound metabolic process	4270	474	257.75	1.84	+	1.08E-36
↳ribonucleotide biosynthetic process	124	28	7.49	3.74	+	2.90E-04
↳nucleotide biosynthetic process	171	37	10.32	3.58	+	5.26E-06
↳nucleoside phosphate biosynthetic process	178	39	10.74	3.63	+	1.32E-06
↳organophosphate biosynthetic process	366	52	22.09	2.35	+	1.42E-03
↳organic substance biosynthetic process	2079	282	125.50	2.25	+	3.44E-31
↳biosynthetic process	2161	292	130.45	2.24	+	3.82E-32
↳heterocycle biosynthetic process	718	79	43.34	1.82	+	1.47E-02
↳cellular biosynthetic process	1985	269	119.82	2.24	+	2.18E-29
↳organic cyclic compound biosynthetic process	848	93	51.19	1.82	+	1.65E-03
↳cellular nitrogen compound biosynthetic process	1118	197	67.49	2.92	+	2.49E-33
↳ribose phosphate biosynthetic process	131	30	7.91	3.79	+	7.18E-05
↳purine nucleotide biosynthetic process	125	29	7.55	3.84	+	9.90E-05
↳purine-containing compound biosynthetic process	130	30	7.85	3.82	+	6.17E-05
↳organonitrogen compound biosynthetic process	1070	197	64.59	3.05	+	1.09E-35
↳coenzyme biosynthetic process	99	24	5.98	4.02	+	8.86E-04
↳coenzyme metabolic process	204	57	12.31	4.63	+	7.53E-15
↳cofactor metabolic process	368	90	22.21	4.05	+	1.57E-21
↳cofactor biosynthetic process	163	35	9.84	3.56	+	1.81E-05
↳amide biosynthetic process	421	138	25.41	5.43	+	6.42E-47
↳cellular amide metabolic process	665	184	40.14	4.58	+	1.00E-54
↳ribonucleoside bisphosphate biosynthetic process	30	13	1.81	7.18	+	4.07E-03
↳ribonucleoside bisphosphate metabolic process	94	36	5.67	6.34	+	7.68E-12
↳nucleoside bisphosphate metabolic process	94	36	5.67	6.34	+	7.68E-12
↳nucleoside bisphosphate biosynthetic process	30	13	1.81	7.18	+	4.07E-03
↳thioester biosynthetic process	20	12	1.21	9.94	+	7.64E-04
↳thioester metabolic process	78	33	4.71	7.01	+	1.22E-11
↳sulfur compound metabolic process	267	68	16.12	4.22	+	2.23E-16
↳sulfur compound biosynthetic process	72	24	4.35	5.52	+	4.85E-06
↳purine nucleoside bisphosphate biosynthetic process	30	13	1.81	7.18	+	4.07E-03
↳purine nucleoside bisphosphate metabolic process	94	36	5.67	6.34	+	7.68E-12
↳acyl-CoA metabolic process	78	33	4.71	7.01	+	1.22E-11
negative regulation of mRNA splicing, via spliceosome	24	14	1.45	9.66	+	8.30E-05

↳regulation of mRNA splicing, via spliceosome	110	46	6.64	6.93	+	4.54E-17
↳regulation of RNA splicing	145	57	8.75	6.51	+	1.04E-20
↳regulation of gene expression	3827	357	231.01	1.55	+	9.96E-13
↳regulation of macromolecule metabolic process	5484	494	331.04	1.49	+	1.09E-17
↳regulation of metabolic process	5976	546	360.73	1.51	+	3.60E-22
↳regulation of RNA metabolic process	3165	255	191.05	1.33	+	3.10E-02
↳regulation of nucleobase-containing compound metabolic process	3401	275	205.30	1.34	+	7.35E-03
↳regulation of primary metabolic process	5349	483	322.89	1.50	+	3.32E-17
↳regulation of nitrogen compound metabolic process	5198	463	313.77	1.48	+	4.95E-15
↳regulation of cellular metabolic process	5552	501	335.14	1.49	+	3.22E-18
↳regulation of cellular process	10826	771	653.50	1.18	+	4.40E-06
↳regulation of mRNA processing	149	56	8.99	6.23	+	1.56E-19
↳regulation of mRNA metabolic process	255	93	15.39	6.04	+	1.82E-33
↳negative regulation of RNA splicing	29	16	1.75	9.14	+	1.33E-05
↳negative regulation of macromolecule metabolic process	2494	253	150.55	1.68	+	2.40E-11
↳negative regulation of metabolic process	2766	281	166.97	1.68	+	3.72E-13
↳negative regulation of biological process	5169	479	312.02	1.54	+	3.15E-19
↳negative regulation of cellular metabolic process	2469	241	149.04	1.62	+	6.06E-09
↳negative regulation of cellular process	4632	423	279.61	1.51	+	7.51E-15
↳negative regulation of nitrogen compound metabolic process	2272	219	137.15	1.60	+	3.10E-07
↳negative regulation of gene expression	1643	169	99.18	1.70	+	1.09E-06
↳negative regulation of mRNA processing	32	16	1.93	8.28	+	3.95E-05
↳negative regulation of mRNA metabolic process	82	33	4.95	6.67	+	3.87E-11
stress granule assembly	16	9	.97	9.32	+	4.84E-02
↳cellular component assembly	1941	244	117.17	2.08	+	1.13E-21
↳cellular component organization	5045	479	304.54	1.57	+	1.56E-21
↳cellular component organization or biogenesis	5239	491	316.25	1.55	+	4.37E-21
↳cellular component biogenesis	2168	259	130.87	1.98	+	1.98E-20
↳organelle organization	3054	307	184.35	1.67	+	2.60E-14
tricarboxylic acid cycle	32	18	1.93	9.32	+	9.90E-07
↳aerobic respiration	66	21	3.98	5.27	+	1.13E-04
↳cellular respiration	129	32	7.79	4.11	+	4.23E-06
↳energy derivation by oxidation of organic compounds	191	45	11.53	3.90	+	6.10E-09
↳generation of precursor metabolites and energy	286	74	17.26	4.29	+	2.08E-18
acetyl-CoA metabolic process	29	16	1.75	9.14	+	1.33E-05
glutamate metabolic process	24	13	1.45	8.97	+	5.46E-04
↳dicarboxylic acid metabolic process	81	36	4.89	7.36	+	1.82E-13
↳glutamine family amino acid metabolic process	57	21	3.44	6.10	+	1.34E-05
↳alpha-amino acid metabolic process	164	56	9.90	5.66	+	6.61E-18
↳cellular amino acid metabolic process	231	79	13.94	5.67	+	1.99E-26
aromatic amino acid family catabolic process	19	10	1.15	8.72	+	2.27E-02
↳aromatic compound catabolic process	292	56	17.63	3.18	+	1.55E-08
↳cellular amino acid catabolic process	90	34	5.43	6.26	+	6.69E-11
↳organonitrogen compound catabolic process	900	116	54.33	2.14	+	5.88E-09
↳organic cyclic compound catabolic process	323	64	19.50	3.28	+	1.07E-10
↳aromatic amino acid family metabolic process	34	13	2.05	6.33	+	1.26E-02
translational elongation	30	15	1.81	8.28	+	1.20E-04

↳cellular macromolecule biosynthetic process	1193	162	72.01	2.25	+	1.04E-15
↳cellular macromolecule metabolic process	3958	386	238.92	1.62	+	1.85E-17
↳macromolecule biosynthetic process	1227	163	74.07	2.20	+	3.59E-15
↳translation	306	112	18.47	6.06	+	4.09E-41
↳cellular protein metabolic process	2844	288	171.67	1.68	+	1.51E-13
↳protein metabolic process	3420	332	206.44	1.61	+	7.77E-14
↳peptide biosynthetic process	326	118	19.68	6.00	+	4.10E-43
↳peptide metabolic process	448	138	27.04	5.10	+	2.60E-44
tRNA aminoacylation for protein translation	40	20	2.41	8.28	+	4.65E-07
↳tRNA aminoacylation	43	20	2.60	7.71	+	1.27E-06
↳amino acid activation	44	20	2.66	7.53	+	1.75E-06
↳tRNA metabolic process	165	28	9.96	2.81	+	4.42E-02
↳ncRNA metabolic process	416	55	25.11	2.19	+	4.57E-03
mRNA destabilization	32	16	1.93	8.28	+	3.95E-05
↳RNA destabilization	35	16	2.11	7.57	+	1.07E-04
↳regulation of RNA stability	109	43	6.58	6.54	+	5.01E-15
↳regulation of cellular catabolic process	698	128	42.13	3.04	+	1.34E-21
↳regulation of catabolic process	843	141	50.89	2.77	+	1.08E-20
↳posttranscriptional regulation of gene expression	427	135	25.78	5.24	+	2.53E-44
↳regulation of biological quality	3959	421	238.98	1.76	+	2.67E-27
↳positive regulation of cellular metabolic process	3270	276	197.39	1.40	+	1.92E-04
↳positive regulation of cellular process	5424	461	327.41	1.41	+	2.09E-11
↳positive regulation of metabolic process	3560	310	214.90	1.44	+	6.54E-07
↳positive regulation of nitrogen compound metabolic process	3086	258	186.28	1.38	+	1.36E-03
↳positive regulation of macromolecule metabolic process	3256	270	196.54	1.37	+	1.32E-03
↳positive regulation of cellular catabolic process	367	72	22.15	3.25	+	3.57E-12
↳positive regulation of catabolic process	436	77	26.32	2.93	+	4.94E-11
↳positive regulation of mRNA catabolic process	50	23	3.02	7.62	+	6.35E-08
↳positive regulation of mRNA metabolic process	93	34	5.61	6.06	+	1.46E-10
↳regulation of mRNA catabolic process	121	49	7.30	6.71	+	7.12E-18
↳regulation of mRNA stability	98	42	5.92	7.10	+	1.13E-15
↳negative regulation of translation	130	46	7.85	5.86	+	9.73E-15
↳negative regulation of cellular protein metabolic process	1004	133	60.61	2.19	+	1.49E-11
↳regulation of cellular protein metabolic process	2531	274	152.78	1.79	+	4.13E-16
↳regulation of protein metabolic process	2723	290	164.37	1.76	+	1.66E-16
↳negative regulation of protein metabolic process	1072	139	64.71	2.15	+	1.35E-11
↳negative regulation of cellular amide metabolic process	149	49	8.99	5.45	+	8.74E-15
↳regulation of cellular amide metabolic process	384	113	23.18	4.87	+	4.91E-34
↳regulation of translation	333	108	20.10	5.37	+	1.52E-35
↳regulation of cellular macromolecule biosynthetic process	3333	271	201.19	1.35	+	5.96E-03
↳regulation of macromolecule biosynthetic process	3419	282	206.38	1.37	+	9.94E-04
↳regulation of biosynthetic process	3644	315	219.97	1.43	+	9.71E-07
↳regulation of cellular biosynthetic process	3571	299	215.56	1.39	+	8.14E-05
production of miRNAs involved in gene silencing by miRNA	27	13	1.63	7.98	+	1.57E-03
↳gene silencing by miRNA	40	17	2.41	7.04	+	8.86E-05
↳posttranscriptional gene silencing by RNA	47	19	2.84	6.70	+	2.34E-05
			4.65	5.16	+	1.48E-05

↳gene silencing by RNA	77	24				
↳posttranscriptional gene silencing	48	20	2.90	6.90	+	5.88E-06
↳production of small RNA involved in gene silencing by RNA	29	13	1.75	7.43	+	2.99E-03
↳dsRNA processing	29	13	1.75	7.43	+	2.99E-03
regulation of alternative mRNA splicing, via spliceosome	66	30	3.98	7.53	+	5.30E-11
translational initiation	55	25	3.32	7.53	+	9.59E-09
NADH metabolic process	27	12	1.63	7.36	+	9.12E-03
alpha-amino acid catabolic process	75	32	4.53	7.07	+	2.72E-11
sulfur amino acid metabolic process	31	13	1.87	6.95	+	5.47E-03
mRNA stabilization	41	17	2.47	6.87	+	1.19E-04
↳negative regulation of mRNA catabolic process	51	19	3.08	6.17	+	6.91E-05
↳negative regulation of RNA catabolic process	61	23	3.68	6.25	+	1.54E-06
↳negative regulation of cellular catabolic process	241	42	14.55	2.89	+	1.09E-04
↳negative regulation of catabolic process	301	46	18.17	2.53	+	1.01E-03
↳positive regulation of gene expression	1995	173	120.43	1.44	+	3.99E-02
↳RNA stabilization	48	19	2.90	6.56	+	3.10E-05
actin filament capping	32	13	1.93	6.73	+	7.30E-03
↳negative regulation of actin filament depolymerization	38	15	2.29	6.54	+	1.41E-03
↳negative regulation of cytoskeleton organization	161	33	9.72	3.40	+	1.39E-04
↳regulation of cytoskeleton organization	559	80	33.74	2.37	+	2.74E-07
↳regulation of organelle organization	1270	156	76.66	2.03	+	1.50E-11
↳regulation of cellular component organization	2537	278	153.14	1.82	+	3.79E-17
↳negative regulation of organelle organization	392	50	23.66	2.11	+	3.26E-02
↳negative regulation of cellular component organization	734	90	44.31	2.03	+	2.18E-05
↳negative regulation of protein depolymerization	72	20	4.35	4.60	+	1.61E-03
↳regulation of protein depolymerization	89	23	5.37	4.28	+	6.08E-04
↳regulation of protein-containing complex disassembly	121	30	7.30	4.11	+	1.48E-05
↳negative regulation of protein-containing complex disassembly	81	20	4.89	4.09	+	7.91E-03
↳negative regulation of supramolecular fiber organization	154	36	9.30	3.87	+	1.45E-06
↳regulation of supramolecular fiber organization	368	59	22.21	2.66	+	2.40E-06
↳regulation of actin filament depolymerization	51	17	3.08	5.52	+	1.57E-03
↳regulation of actin polymerization or depolymerization	185	34	11.17	3.04	+	8.65E-04
↳regulation of actin filament organization	271	40	16.36	2.45	+	1.33E-02
↳regulation of actin filament-based process	409	54	24.69	2.19	+	6.27E-03
↳regulation of actin filament length	188	34	11.35	3.00	+	1.21E-03
↳regulation of cellular component size	407	62	24.57	2.52	+	5.18E-06
↳regulation of anatomical structure size	571	84	34.47	2.44	+	3.47E-08
↳negative regulation of actin filament polymerization	58	18	3.50	5.14	+	1.67E-03
↳regulation of actin filament polymerization	168	32	10.14	3.16	+	1.00E-03
↳regulation of protein polymerization	224	44	13.52	3.25	+	1.92E-06
↳regulation of protein-containing complex assembly	434	64	26.20	2.44	+	9.04E-06
↳regulation of cellular component biogenesis	964	113	58.19	1.94	+	2.14E-06
↳negative regulation of protein polymerization	73	22	4.41	4.99	+	1.17E-04
↳negative regulation of protein-containing complex assembly	136	28	8.21	3.41	+	1.55E-03
nuclear migration	31	12	1.87	6.41	+	2.89E-02
↳organelle localization	474	70	28.61	2.45	+	1.87E-06
↳cellular localization	2053	268	123.93	2.16	+	9.79E-27
			293.61	1.50	+	4.79E-15

↳localization	4864	440				
↳intracellular transport	1182	183	71.35	2.56	+	2.74E-24
↳transport	3572	347	215.62	1.61	+	9.82E-15
↳establishment of localization	3711	358	224.01	1.60	+	5.99E-15
↳establishment of organelle localization	331	55	19.98	2.75	+	4.04E-06
positive regulation of translation	123	46	7.42	6.20	+	1.65E-15
↳positive regulation of cellular protein metabolic process	1564	143	94.41	1.51	+	2.49E-02
↳positive regulation of protein metabolic process	1673	151	100.99	1.50	+	2.31E-02
↳positive regulation of cellular amide metabolic process	149	50	8.99	5.56	+	1.92E-15
serine family amino acid metabolic process	33	12	1.99	6.02	+	4.88E-02
cortical actin cytoskeleton organization	42	15	2.54	5.92	+	4.03E-03
↳cortical cytoskeleton organization	59	18	3.56	5.05	+	2.07E-03
↳cytoskeleton organization	1070	131	64.59	2.03	+	5.21E-09
↳actin cytoskeleton organization	500	75	30.18	2.48	+	1.54E-07
↳actin filament-based process	555	80	33.50	2.39	+	2.16E-07
alpha-amino acid biosynthetic process	51	18	3.08	5.85	+	3.36E-04
↳cellular amino acid biosynthetic process	55	19	3.32	5.72	+	1.87E-04
↳carboxylic acid biosynthetic process	224	50	13.52	3.70	+	1.89E-09
↳organic acid biosynthetic process	225	51	13.58	3.76	+	6.52E-10
↳small molecule biosynthetic process	446	76	26.92	2.82	+	3.83E-10
cellular aldehyde metabolic process	52	18	3.14	5.73	+	4.29E-04
positive regulation of RNA splicing	50	17	3.02	5.63	+	1.24E-03
regulation of translational initiation	59	20	3.56	5.62	+	1.04E-04
pyruvate metabolic process	69	22	4.17	5.28	+	4.96E-05
cytoplasmic translation	64	20	3.86	5.18	+	3.21E-04
ribosome assembly	62	19	3.74	5.08	+	9.06E-04
↳ribonucleoprotein complex biogenesis	392	62	23.66	2.62	+	1.86E-06
cofactor catabolic process	56	17	3.38	5.03	+	4.72E-03
mitochondrial translation	52	15	3.14	4.78	+	3.69E-02
↳mitochondrial gene expression	81	21	4.89	4.29	+	2.14E-03
maintenance of protein location in cell	77	22	4.65	4.73	+	2.61E-04
↳maintenance of location in cell	103	27	6.22	4.34	+	3.49E-05
↳maintenance of location	173	35	10.44	3.35	+	6.89E-05
↳cellular protein localization	1423	189	85.90	2.20	+	3.82E-18
↳cellular macromolecule localization	1430	190	86.32	2.20	+	2.73E-18
↳macromolecule localization	2266	273	136.78	2.00	+	2.72E-22
↳protein localization	1963	238	118.49	2.01	+	5.22E-19
↳maintenance of protein location	108	26	6.52	3.99	+	2.96E-04
antibiotic metabolic process	91	26	5.49	4.73	+	1.47E-05
↳drug metabolic process	412	87	24.87	3.50	+	4.61E-17
glutathione metabolic process	58	16	3.50	4.57	+	2.89E-02
↳cellular modified amino acid metabolic process	156	34	9.42	3.61	+	2.21E-05
protein localization to endoplasmic reticulum	59	16	3.56	4.49	+	3.48E-02
↳protein localization to organelle	622	93	37.55	2.48	+	6.24E-10
purine ribonucleoside triphosphate metabolic process	59	16	3.56	4.49	+	3.48E-02
↳ribonucleoside triphosphate metabolic process	63	17	3.80	4.47	+	1.85E-02
nuclear-transcribed mRNA catabolic process	94	25	5.67	4.41	+	1.02E-04
↳mRNA catabolic process	119	30	7.18	4.18	+	1.06E-05
↳RNA catabolic process	147	32	8.87	3.61	+	6.63E-05

↳nucleobase-containing compound catabolic process	234	37	14.13	2.62	+	7.63E-03
↳heterocycle catabolic process	278	52	16.78	3.10	+	2.16E-07
↳cellular nitrogen compound catabolic process	272	51	16.42	3.11	+	3.12E-07
↳cellular macromolecule catabolic process	762	88	46.00	1.91	+	5.34E-04
↳macromolecule catabolic process	858	101	51.79	1.95	+	1.96E-05
ribonucleoprotein complex assembly	163	43	9.84	4.37	+	7.69E-10
↳cellular protein-containing complex assembly	653	92	39.42	2.33	+	2.36E-08
↳protein-containing complex assembly	1003	141	60.54	2.33	+	1.63E-14
↳protein-containing complex subunit organization	1144	156	69.06	2.26	+	3.49E-15
↳ribonucleoprotein complex subunit organization	170	45	10.26	4.39	+	1.84E-10
mRNA transport	107	28	6.46	4.34	+	1.86E-05
↳RNA transport	150	37	9.05	4.09	+	2.18E-07
↳nucleic acid transport	150	37	9.05	4.09	+	2.18E-07
↳nucleobase-containing compound transport	183	39	11.05	3.53	+	2.66E-06
↳organic substance transport	1856	216	112.04	1.93	+	5.08E-15
↳nitrogen compound transport	1550	190	93.56	2.03	+	8.20E-15
↳establishment of RNA localization	152	37	9.18	4.03	+	3.02E-07
↳RNA localization	170	42	10.26	4.09	+	9.48E-09
carbohydrate catabolic process	88	23	5.31	4.33	+	5.10E-04
↳carbohydrate metabolic process	405	62	24.45	2.54	+	4.43E-06
axo-dendritic transport	73	18	4.41	4.08	+	2.80E-02
↳transport along microtubule	155	29	9.36	3.10	+	5.38E-03
↳movement of cell or subcellular component	1395	133	84.21	1.58	+	7.12E-03
↳cytoskeleton-dependent intracellular transport	186	34	11.23	3.03	+	9.68E-04
regulation of telomere maintenance	81	19	4.89	3.89	+	2.82E-02
protein folding	146	34	8.81	3.86	+	5.07E-06
positive regulation of viral process	82	19	4.95	3.84	+	3.29E-02
↳regulation of viral process	183	32	11.05	2.90	+	5.39E-03
↳regulation of symbiosis, encompassing mutualism through parasitism	200	36	12.07	2.98	+	5.80E-04
protein export from nucleus	91	21	5.49	3.82	+	1.09E-02
↳nucleocytoplasmic transport	203	36	12.25	2.94	+	8.04E-04
↳nuclear transport	203	36	12.25	2.94	+	8.04E-04
↳intracellular protein transport	765	109	46.18	2.36	+	7.39E-11
↳protein transport	1246	167	75.21	2.22	+	6.22E-16
↳establishment of protein localization	1330	179	80.28	2.23	+	1.78E-17
↳peptide transport	1276	168	77.02	2.18	+	2.67E-15
↳amide transport	1301	169	78.53	2.15	+	5.89E-15
post-Golgi vesicle-mediated transport	88	20	5.31	3.77	+	2.37E-02
↳Golgi vesicle transport	250	49	15.09	3.25	+	1.95E-07
↳vesicle-mediated transport	1271	141	76.72	1.84	+	4.09E-07
protein stabilization	174	39	10.50	3.71	+	7.44E-07
↳regulation of protein stability	278	49	16.78	2.92	+	4.82E-06
endoplasmic reticulum to Golgi vesicle-mediated transport	105	23	6.34	3.63	+	7.55E-03
ATP metabolic process	170	37	10.26	3.61	+	4.57E-06
regulation of viral genome replication	93	20	5.61	3.56	+	4.89E-02
drug catabolic process	148	31	8.93	3.47	+	2.45E-04
cytosolic transport	132	27	7.97	3.39	+	2.85E-03

regulation of cell shape	158	31	9.54	3.25	+	8.88E-04
↳regulation of cell morphogenesis	537	83	32.42	2.56	+	3.54E-09
↳regulation of anatomical structure morphogenesis	1090	124	65.80	1.88	+	1.70E-06
↳regulation of developmental process	2684	239	162.02	1.48	+	3.13E-05
receptor-mediated endocytosis	138	27	8.33	3.24	+	6.11E-03
positive regulation of endocytosis	123	24	7.42	3.23	+	2.60E-02
↳positive regulation of transport	1099	131	66.34	1.97	+	2.57E-08
↳regulation of transport	1972	207	119.04	1.74	+	8.79E-10
↳positive regulation of cellular component organization	1254	153	75.70	2.02	+	4.56E-11
↳regulation of vesicle-mediated transport	598	77	36.10	2.13	+	5.12E-05
regulation of nucleocytoplasmic transport	125	24	7.55	3.18	+	3.32E-02
↳regulation of intracellular transport	352	59	21.25	2.78	+	7.42E-07
monocarboxylic acid biosynthetic process	138	26	8.33	3.12	+	1.80E-02
monosaccharide metabolic process	156	29	9.42	3.08	+	6.03E-03
establishment of vesicle localization	142	26	8.57	3.03	+	2.86E-02
↳establishment of localization in cell	353	47	21.31	2.21	+	2.72E-02
↳vesicle localization	154	27	9.30	2.90	+	3.81E-02
positive regulation of intracellular transport	198	36	11.95	3.01	+	4.65E-04
protein import	145	26	8.75	2.97	+	3.99E-02
establishment of protein localization to organelle	302	54	18.23	2.96	+	3.98E-07
cellular carbohydrate metabolic process	146	26	8.81	2.95	+	4.45E-02
positive regulation of cell morphogenesis involved in differentiation	189	33	11.41	2.89	+	3.72E-03
↳regulation of cell morphogenesis involved in differentiation	348	52	21.01	2.48	+	2.26E-04
↳regulation of cell development	1092	112	65.92	1.70	+	2.37E-03
actin filament organization	241	42	14.55	2.89	+	1.09E-04
↳supramolecular fiber organization	465	73	28.07	2.60	+	4.53E-08
regulation of microtubule cytoskeleton organization	203	35	12.25	2.86	+	2.19E-03
↳regulation of microtubule-based process	241	40	14.55	2.75	+	7.69E-04
response to metal ion	270	46	16.30	2.82	+	4.14E-05
↳response to inorganic substance	417	69	25.17	2.74	+	2.21E-08
↳response to chemical	3484	341	210.31	1.62	+	6.50E-15
positive regulation of protein-containing complex assembly	221	37	13.34	2.77	+	1.92E-03
↳positive regulation of cellular component biogenesis	524	64	31.63	2.02	+	6.90E-03
endomembrane system organization	367	61	22.15	2.75	+	4.05E-07
protein-containing complex localization	206	34	12.43	2.73	+	7.83E-03
endosomal transport	203	32	12.25	2.61	+	4.70E-02
positive regulation of developmental growth	216	34	13.04	2.61	+	2.36E-02
↳positive regulation of growth	299	42	18.05	2.33	+	2.33E-02
protein complex oligomerization	229	35	13.82	2.53	+	2.73E-02
negative regulation of apoptotic signaling pathway	232	35	14.00	2.50	+	3.46E-02
↳negative regulation of apoptotic process	906	113	54.69	2.07	+	8.52E-08
↳negative regulation of programmed cell death	926	114	55.90	2.04	+	1.39E-07
↳regulation of programmed cell death	1518	163	91.63	1.78	+	1.07E-07
↳regulation of cell death	1672	183	100.93	1.81	+	9.26E-10
↳negative regulation of cell death	1041	127	62.84	2.02	+	1.20E-08
↳regulation of apoptotic process	1493	161	90.12	1.79	+	1.09E-07
positive regulation of organelle organization	596	87	35.98	2.42	+	1.53E-08
positive regulation of protein transport	443	63	26.74	2.36	+	4.44E-05
↳regulation of protein transport	739	94	44.61	2.11	+	1.94E-06

↳regulation of peptide transport	781	95	47.14	2.02	+	1.44E-05
response to oxidative stress	337	47	20.34	2.31	+	8.96E-03
↳response to stress	3164	261	190.99	1.37	+	3.41E-03
negative regulation of proteolysis	345	46	20.83	2.21	+	3.64E-02
↳regulation of proteolysis	717	78	43.28	1.80	+	2.23E-02
cell part morphogenesis	507	66	30.60	2.16	+	6.21E-04
↳cellular component morphogenesis	844	98	50.95	1.92	+	5.75E-05
↳developmental process	5570	451	336.23	1.34	+	1.02E-07
↳anatomical structure development	5212	414	314.62	1.32	+	1.61E-05
↳cellular developmental process	3783	305	228.36	1.34	+	2.06E-03
negative regulation of hydrolase activity	401	51	24.21	2.11	+	2.67E-02
↳negative regulation of catalytic activity	701	76	42.32	1.80	+	3.64E-02
↳negative regulation of molecular function	1034	106	62.42	1.70	+	4.80E-03
↳regulation of molecular function	2559	242	154.47	1.57	+	1.15E-07
↳regulation of catalytic activity	1881	177	113.54	1.56	+	1.84E-04
↳regulation of hydrolase activity	994	118	60.00	1.97	+	4.18E-07
cell junction organization	466	56	28.13	1.99	+	4.78E-02
cellular response to nitrogen compound	512	61	30.91	1.97	+	2.21E-02
↳response to nitrogen compound	851	99	51.37	1.93	+	4.32E-05
↳cellular response to chemical stimulus	2359	234	142.40	1.64	+	2.99E-09
neuron projection development	691	82	41.71	1.97	+	4.84E-04
↳neuron development	843	89	50.89	1.75	+	1.43E-02
↳generation of neurons	1663	154	100.38	1.53	+	4.38E-03
↳neurogenesis	1771	161	106.90	1.51	+	6.92E-03
↳cell differentiation	3696	293	223.10	1.31	+	1.63E-02
↳system development	4226	338	255.10	1.32	+	6.50E-04
↳multicellular organism development	4844	384	292.40	1.31	+	1.32E-04
↳cell development	1750	167	105.64	1.58	+	2.08E-04
↳plasma membrane bounded cell projection organization	1060	106	63.99	1.66	+	1.68E-02
cell morphogenesis	736	87	44.43	1.96	+	2.12E-04
response to organic cyclic compound	621	72	37.49	1.92	+	9.15E-03
↳response to organic substance	2488	251	150.19	1.67	+	6.41E-11
response to organonitrogen compound	745	85	44.97	1.89	+	1.39E-03
response to hormone	611	69	36.88	1.87	+	2.77E-02
↳response to endogenous stimulus	1126	118	67.97	1.74	+	3.27E-04
regulation of plasma membrane bounded cell projection organization	781	87	47.14	1.85	+	2.59E-03
↳regulation of cell projection organization	790	87	47.69	1.82	+	4.42E-03
cellular response to cytokine stimulus	639	71	38.57	1.84	+	4.30E-02
↳cellular response to organic substance	1822	173	109.98	1.57	+	1.54E-04
↳response to cytokine	746	86	45.03	1.91	+	9.26E-04
cellular homeostasis	857	93	51.73	1.80	+	2.72E-03
↳homeostatic process	1644	155	99.24	1.56	+	1.63E-03
cellular response to oxygen-containing compound	912	95	55.05	1.73	+	1.18E-02
↳response to oxygen-containing compound	1296	131	78.23	1.67	+	4.24E-04
regulation of cell migration	914	93	55.17	1.69	+	3.46E-02
↳regulation of cell motility	965	99	58.25	1.70	+	1.16E-02
↳regulation of locomotion	1042	112	62.90	1.78	+	2.17E-04
↳regulation of cellular component movement	1054	116	63.62	1.82	+	3.70E-05

regulation of response to stress	1295	124	78.17	1.59	+	1.35E-02
cellular response to stress	1446	137	87.29	1.57	+	6.15E-03
positive regulation of molecular function	1477	139	89.16	1.56	+	7.72E-03
regulation of multicellular organismal process	3223	278	194.55	1.43	+	2.39E-05
Unclassified	1901	34	114.75	.30	-	0.00E00
G protein-coupled receptor signaling pathway	1851	18	111.73	.16	-	2.61E-24
↳signal transduction	4827	197	291.38	.68	-	2.14E-06
↳cell communication	5260	235	317.51	.74	-	1.06E-03
↳signaling	5138	218	310.15	.70	-	1.58E-05
positive regulation of B cell activation	269	1	16.24	.06	-	3.53E-02
sensory perception of chemical stimulus	1228	1	74.13	.01	-	7.81E-27
↳sensory perception	1642	17	99.12	.17	-	2.51E-20
↳nervous system process	2084	55	125.80	.44	-	7.65E-09

About | Release Information | Contact Us | System Requirements | Privacy Policy | Disclaimer
© Copyright 2020 Paul Thomas All Rights Reserved.

KIDNEY - MF

PANTHER - Compare lists to reference list

LOGIN REGISTER CONTACT US

Home | About | PANTHER Data | PANTHER Tools | PANTHER Services | Workspace | Downloads | Help/Tutorial

PANTHER 15.0 released!

Analysis Summary: Please report in publication

Analysis Type: PANTHER Overrepresentation Test (Released 20200407)

Annotation Version and Release Date: GO Ontology database Released 2020-02-21

Analyzed List: upload_1 (Mus musculus) Change

Reference List: Mus musculus (all genes in database) Change

Annotation Data Set: GO molecular function complete

Test Type: (•) Fisher's Exact ( ) Binomial

Correction: ( ) Calculate False Discovery Rate (•) Use the Bonferroni correction for multiple testing ( ) No correction

Results

	Reference list	upload_1
Uniquely Mapped IDS:	22265 out of 22265	1344 out of 1344
Unmapped IDs:	0	0
Multiple mapping information:	0	0

Bonferroni count: 2756

Export Table XML with user input ids JSON with user input ids

Displaying only results for Bonferroni-corrected for P < 0.05, click here to display all results

	Mus musculus (REF)	upload_1 (Hierarchy NEW!)				
GO molecular function complete	#	#	expected	Fold Enrichment	+/-	P value
ligase activity, forming carbon-carbon bonds	7	7	.42	16.57	+	1.27E-02
↳ligase activity	146	57	8.81	6.47	+	4.26E-21
↳catalytic activity	5659	564	341.60	1.65	+	6.60E-34
N6-methyladenosine-containing RNA binding	8	7	.48	14.50	+	2.26E-02
↳RNA binding	1086	338	65.56	5.16	+	4.40E-121
↳nucleic acid binding	3161	389	190.81	2.04	+	1.15E-37
↳organic cyclic compound binding	5175	660	312.38	2.11	+	7.59E-85
↳binding	13351	1179	805.92	1.46	+	2.91E-103
↳heterocyclic compound binding	5072	650	306.17	2.12	+	1.15E-83
acyl-CoA dehydrogenase activity	10	8	.60	13.25	+	7.79E-03
↳oxidoreductase activity, acting on the CH-CH group of donors	56	16	3.38	4.73	+	6.11E-03
↳oxidoreductase activity	791	117	47.75	2.45	+	2.11E-13
RNA stem-loop binding	14	10	.85	11.83	+	8.96E-04
translation elongation factor activity	17	11	1.03	10.72	+	4.70E-04
↳translation factor activity, RNA binding	77	39	4.65	8.39	+	7.46E-17
↳translation regulator activity, nucleic acid binding	95	45	5.73	7.85	+	7.47E-19
↳translation regulator activity	126	60	7.61	7.89	+	6.02E-26
mRNA 5'-UTR binding	24	14	1.45	9.66	+	2.57E-05
↳mRNA binding	272	120	16.42	7.31	+	1.08E-51
miRNA binding	30	17	1.81	9.39	+	9.28E-07
↳regulatory RNA binding	43	18	2.60	6.93	+	1.23E-05
acid-thiol ligase activity	27	15	1.63	9.20	+	1.25E-05

↳ligase activity, forming carbon-sulfur bonds	38	17	2.29	7.41	+	1.50E-05
CoA-ligase activity	24	12	1.45	8.28	+	1.06E-03
aminoacyl-tRNA ligase activity	41	20	2.47	8.08	+	2.03E-07
↳ligase activity, forming carbon-oxygen bonds	41	20	2.47	8.08	+	2.03E-07
↳catalytic activity, acting on RNA	337	63	20.34	3.10	+	5.28E-10
translation initiation factor activity	48	23	2.90	7.94	+	1.02E-08
C-acyltransferase activity	21	10	1.27	7.89	+	1.40E-02
RNA helicase activity	53	25	3.20	7.81	+	1.56E-09
↳helicase activity	148	35	8.93	3.92	+	6.18E-07
↳ATPase activity, coupled	279	51	16.84	3.03	+	2.15E-07
↳ATPase activity	412	69	24.87	2.77	+	4.83E-09
↳nucleoside-triphosphatase activity	748	112	45.15	2.48	+	4.36E-13
↳pyrophosphatase activity	801	117	48.35	2.42	+	6.11E-13
↳hydrolase activity, acting on acid anhydrides, in phosphorus-containing anhydrides	804	117	48.53	2.41	+	6.87E-13
↳hydrolase activity, acting on acid anhydrides	804	117	48.53	2.41	+	6.87E-13
↳hydrolase activity	2449	242	147.83	1.64	+	5.67E-10
double-stranded RNA binding	82	38	4.95	7.68	+	2.40E-15
poly(A) binding	24	11	1.45	7.59	+	6.32E-03
↳poly-purine tract binding	32	16	1.93	8.28	+	1.22E-05
↳single-stranded RNA binding	89	38	5.37	7.07	+	2.11E-14
oxidoreductase activity, acting on the CH-NH group of donors	27	12	1.63	7.36	+	2.83E-03
mRNA 3'-UTR binding	87	38	5.25	7.24	+	1.15E-14
NAD binding	61	26	3.68	7.06	+	3.37E-09
↳coenzyme binding	288	82	17.38	4.72	+	1.56E-23
↳cofactor binding	531	108	32.05	3.37	+	2.76E-21
↳nucleotide binding	2030	316	122.54	2.58	+	1.32E-47
↳small molecule binding	2421	370	146.14	2.53	+	8.96E-56
↳nucleoside phosphate binding	2030	316	122.54	2.58	+	1.32E-47
ADP binding	40	17	2.41	7.04	+	2.75E-05
↳adenyl ribonucleotide binding	1453	215	87.71	2.45	+	1.66E-27
↳adenyl nucleotide binding	1464	219	88.37	2.48	+	1.55E-28
↳purine nucleotide binding	1798	264	108.53	2.43	+	1.79E-34
↳purine ribonucleotide binding	1786	260	107.81	2.41	+	3.02E-33
↳ribonucleotide binding	1802	263	108.78	2.42	+	7.80E-34
↳carbohydrate derivative binding	2123	294	128.15	2.29	+	7.95E-35
↳anion binding	2703	391	163.16	2.40	+	6.47E-54
↳ion binding	5485	575	331.10	1.74	+	1.93E-41
poly(U) RNA binding	26	11	1.57	7.01	+	1.17E-02
↳poly-pyrimidine tract binding	30	13	1.81	7.18	+	1.26E-03
ribosome binding	64	27	3.86	6.99	+	1.51E-09
↳ribonucleoprotein complex binding	146	46	8.81	5.22	+	1.22E-13
↳protein-containing complex binding	1451	252	87.59	2.88	+	7.63E-44
rRNA binding	70	28	4.23	6.63	+	1.60E-09
pre-mRNA binding	33	13	1.99	6.53	+	2.99E-03
oxidoreductase activity, acting on the aldehyde or oxo group of donors, NAD or NADP as acceptor	47	18	2.84	6.34	+	3.74E-05
↳oxidoreductase activity, acting on the aldehyde or oxo group of donors	56	19	3.38	5.62	+	7.37E-05
Ran GTPase binding	34	12	2.05	5.85	+	1.94E-02
↳Ras GTPase binding	399	57	24.09	2.37	+	7.94E-05
↳small GTPase binding	416	59	25.11	2.35	+	4.92E-05
↳GTPase binding	515	73	31.09	2.35	+	9.78E-07
↳enzyme binding	2331	306	140.71	2.17	+	1.43E-32

↳protein binding	9112	865	550.03	1.57	+	1.27E-59
extracellular matrix structural constituent	143	48	8.63	5.56	+	3.10E-15
↳structural molecule activity	576	151	34.77	4.34	+	4.40E-42
structural constituent of cytoskeleton	64	21	3.86	5.44	+	2.24E-05
hydro-lyase activity	52	17	3.14	5.42	+	6.12E-04
↳carbon-oxygen lyase activity	62	18	3.74	4.81	+	1.18E-03
↳lyase activity	184	38	11.11	3.42	+	2.99E-06
flavin adenine dinucleotide binding	82	26	4.95	5.25	+	7.11E-07
structural constituent of ribosome	161	49	9.72	5.04	+	3.73E-14
unfolded protein binding	92	28	5.55	5.04	+	3.24E-07
NADP binding	47	14	2.84	4.93	+	1.75E-02
amino acid binding	71	21	4.29	4.90	+	1.01E-04
↳carboxylic acid binding	223	45	13.46	3.34	+	1.73E-07
↳organic acid binding	237	46	14.31	3.22	+	3.28E-07
tRNA binding	60	17	3.62	4.69	+	3.27E-03
actin filament binding	201	56	12.13	4.62	+	5.27E-15
↳actin binding	426	93	25.71	3.62	+	8.01E-20
↳cytoskeletal protein binding	976	163	58.92	2.77	+	9.40E-25
pyridoxal phosphate binding	51	14	3.08	4.55	+	3.82E-02
↳vitamin B6 binding	52	14	3.14	4.46	+	4.60E-02
↳drug binding	1644	242	99.24	2.44	+	3.15E-31
↳vitamin binding	132	27	7.97	3.39	+	8.84E-04
modified amino acid binding	100	24	6.04	3.98	+	3.22E-04
heat shock protein binding	140	33	8.45	3.90	+	2.16E-06
chaperone binding	101	22	6.10	3.61	+	4.36E-03
single-stranded DNA binding	111	23	6.70	3.43	+	5.34E-03
oxidoreductase activity, acting on the CH-OH group of donors, NAD or NADP as acceptor	141	28	8.51	3.29	+	9.11E-04
↳oxidoreductase activity, acting on CH-OH group of donors	149	30	8.99	3.34	+	2.69E-04
integrin binding	133	26	8.03	3.24	+	3.07E-03
↳cell adhesion molecule binding	245	39	14.79	2.64	+	1.04E-03
ion channel binding	140	25	8.45	2.96	+	2.00E-02
protein C-terminus binding	231	39	13.94	2.80	+	2.32E-04
magnesium ion binding	197	32	11.89	2.69	+	1.06E-02
↳metal ion binding	3499	299	211.21	1.42	+	3.71E-06
↳cation binding	3598	311	217.19	1.43	+	4.02E-07
ubiquitin protein ligase binding	308	48	18.59	2.58	+	1.19E-04
↳ubiquitin-like protein ligase binding	323	50	19.50	2.56	+	6.29E-05
GTP binding	355	55	21.43	2.57	+	1.06E-05
↳purine ribonucleoside triphosphate binding	1713	247	103.40	2.39	+	9.31E-31
↳purine ribonucleoside binding	362	57	21.85	2.61	+	3.87E-06
↳ribonucleoside binding	365	58	22.03	2.63	+	1.40E-06
↳nucleoside binding	375	59	22.64	2.61	+	1.89E-06
↳purine nucleoside binding	366	57	22.09	2.58	+	4.73E-06
↳guanyl ribonucleotide binding	379	58	22.88	2.54	+	5.21E-06
↳guanyl nucleotide binding	379	58	22.88	2.54	+	5.21E-06
microtubule binding	247	38	14.91	2.55	+	2.79E-03
↳tubulin binding	346	49	20.89	2.35	+	7.74E-04
sulfur compound binding	255	38	15.39	2.47	+	6.87E-03
amide binding	378	56	22.82	2.45	+	3.26E-05
phospholipid binding	427	63	25.78	2.44	+	3.77E-06
↳lipid binding	763	92	46.06	2.00	+	1.19E-05
ATP binding	1390	203	83.91	2.42	+	5.34E-25

protein kinase binding	727	93	43.88	2.12	+	7.15E-07
↳kinase binding	813	99	49.08	2.02	+	1.52E-06
protein domain specific binding	786	100	47.45	2.11	+	1.69E-07
protein homodimerization activity	648	75	39.12	1.92	+	1.36E-03
↳identical protein binding	1983	271	119.70	2.26	+	1.01E-30
↳protein dimerization activity	945	99	57.04	1.74	+	1.43E-03
calcium ion binding	612	67	36.94	1.81	+	2.97E-02
enzyme regulator activity	989	98	59.70	1.64	+	1.72E-02
Unclassified	2116	37	127.73	.29	-	0.00E00
receptor ligand activity	488	6	29.46	.20	-	1.61E-03
↳signaling receptor activator activity	493	6	29.76	.20	-	1.12E-03
↳receptor regulator activity	534	8	32.23	.25	-	3.28E-03
DNA-binding transcription activator activity, RNA polymerase II-specific	472	5	28.49	.18	-	5.96E-04
↳DNA-binding transcription activator activity	475	5	28.67	.17	-	6.18E-04
↳DNA-binding transcription factor activity	1066	20	64.35	.31	-	7.44E-07
↳transcription regulator activity	1445	49	87.23	.56	-	2.96E-02
↳DNA-binding transcription factor activity, RNA polymerase II-specific	814	13	49.14	.26	-	6.78E-06
odorant binding	473	2	28.55	.07	-	1.74E-06
G protein-coupled receptor activity	750	1	45.27	.02	-	7.86E-15
↳transmembrane signaling receptor activity	2139	5	129.12	.04	-	1.49E-45
↳signaling receptor activity	2339	13	141.19	.09	-	2.23E-41
↳molecular transducer activity	2344	13	141.49	.09	-	1.45E-41

About | Release Information | Contact Us | System Requirements | Privacy Policy | Disclaimer

© Copyright 2020 Paul Thomas All Rights Reserved.

LOGIN REGISTER CONTACT US

Home | About | PANTHER Data | PANTHER Tools | PANTHER Services | Workspace | Downloads | Help/Tutorial

PANTHER 15.0 released!

Analysis Summary: Please report in publication

Analysis Type: PANTHER Overrepresentation Test (Released 20200407)

Annotation Version and Release Date: GO Ontology database Released 2020-02-21

Analyzed List: upload_1 (Mus musculus) Change

Reference List: Mus musculus (all genes in database) Change

Annotation Data Set: GO cellular component complete

Test Type: Fisher's Exact Binomial

Correction: Calculate False Discovery Rate Use the Bonferroni correction for multiple testing No correction

Results

	Reference list	upload_1
Uniquely Mapped IDS:	22265 out of 22265	1344 out of 1344
Unmapped IDs:	0	0
Multiple mapping information:	0	0

Bonferroni count: 1452

Export Table XML with user input ids JSON with user input ids

Displaying only results for Bonferroni-corrected for P < 0.05, click here to display all results

	Mus musculus (REF)	upload_1 (Hierarchy NEW!)				
GO cellular component complete	#	#	expected	Fold Enrichment	+/-	P value
eukaryotic translation initiation factor 3 complex, eIF3m	8	7	.48	14.50	+	1.19E-02
↳eukaryotic translation initiation factor 3 complex	15	12	.91	13.25	+	1.25E-05
↳cytoplasm	10945	1140	660.68	1.73	+	2.25E-153
↳cellular anatomical entity	18638	1300	1125.06	1.16	+	3.10E-47
↳intracellular	13691	1234	826.44	1.49	+	4.05E-133
↳protein-containing complex	5309	630	320.47	1.97	+	2.03E-67
chaperonin-containing T-complex	10	8	.60	13.25	+	4.11E-03
↳chaperone complex	22	14	1.33	10.54	+	5.93E-06
↳cytosol	3533	541	213.27	2.54	+	4.05E-91
aminoacyl-tRNA synthetase multienzyme complex	12	9	.72	12.42	+	1.40E-03
zona pellucida receptor complex	13	9	.78	11.47	+	2.25E-03
endoplasmic reticulum chaperone complex	12	8	.72	11.04	+	1.07E-02

↳endoplasmic reticulum	1639	187	98.94	1.89	+	2.08E-12
↳endomembrane system	3884	380	234.45	1.62	+	4.47E-18
↳intracellular membrane-bounded organelle	10291	974	621.20	1.57	+	7.37E-77
↳intracellular organelle	11954	1102	721.59	1.53	+	7.78E-97
↳organelle	12303	1111	742.66	1.50	+	4.55E-92
↳membrane-bounded organelle	11048	1014	666.90	1.52	+	6.09E-76
messenger ribonucleoprotein complex	13	8	.78	10.19	+	1.64E-02
↳ribonucleoprotein complex	703	185	42.44	4.36	+	4.73E-53
proton-transporting two-sector ATPase complex, catalytic domain	14	8	.85	9.47	+	2.45E-02
cytoplasmic stress granule	66	35	3.98	8.79	+	1.12E-15
↳cytoplasmic ribonucleoprotein granule	206	73	12.43	5.87	+	8.90E-26
↳ribonucleoprotein granule	217	80	13.10	6.11	+	2.06E-29
↳intracellular non-membrane-bounded organelle	4162	477	251.23	1.90	+	1.72E-41
↳non-membrane-bounded organelle	4181	479	252.38	1.90	+	1.32E-41
↳supramolecular complex	1197	211	72.26	2.92	+	6.85E-37
postsynaptic cytoskeleton	19	10	1.15	8.72	+	3.71E-03
↳postsynapse	727	120	43.88	2.73	+	2.57E-17
↳synapse	1435	225	86.62	2.60	+	1.42E-32
↳cell junction	2028	284	122.42	2.32	+	1.95E-34
↳cytoskeleton	2135	241	128.88	1.87	+	1.70E-16
smooth endoplasmic reticulum	36	18	2.17	8.28	+	6.98E-07
mitochondrial nucleoid	47	22	2.84	7.75	+	2.27E-08
↳mitochondrial matrix	272	91	16.42	5.54	+	4.42E-31
↳intracellular organelle lumen	4311	467	260.23	1.79	+	2.68E-34
↳organelle lumen	4312	467	260.29	1.79	+	3.97E-34
↳membrane-enclosed lumen	4312	467	260.29	1.79	+	3.97E-34
↳mitochondrion	1803	321	108.84	2.95	+	1.19E-60
↳nucleoid	47	22	2.84	7.75	+	2.27E-08
polysomal ribosome	30	14	1.81	7.73	+	1.17E-04
↳polysome	73	35	4.41	7.94	+	1.30E-14
↳ribosome	234	70	14.13	4.96	+	4.65E-21
postsynaptic cytosol	26	12	1.57	7.65	+	1.09E-03
↳region of cytosol	40	14	2.41	5.80	+	1.93E-03
myelin sheath	213	96	12.86	7.47	+	1.20E-41
cytosolic small ribosomal subunit	46	20	2.78	7.20	+	5.30E-07
↳small ribosomal subunit	78	31	4.71	6.58	+	5.59E-11
↳ribosomal subunit	202	60	12.19	4.92	+	1.20E-17
↳cytosolic ribosome	117	37	7.06	5.24	+	6.87E-11
proteasome accessory complex	26	10	1.57	6.37	+	3.27E-02
↳proteasome complex	66	19	3.98	4.77	+	3.34E-04
↳endopeptidase complex	67	19	4.04	4.70	+	4.06E-04
↳peptidase complex	93	20	5.61	3.56	+	7.98E-03

↳catalytic complex	1341	122	80.95	1.51	+	2.84E-02
intercalated disc	62	21	3.74	5.61	+	7.44E-06
↳cell-cell contact zone	85	23	5.13	4.48	+	4.86E-05
↳cell-cell junction	497	58	30.00	1.93	+	1.47E-02
↳anchoring junction	619	80	37.37	2.14	+	3.41E-06
neuron projection cytoplasm	39	13	2.35	5.52	+	7.14E-03
↳cytoplasmic region	216	32	13.04	2.45	+	2.99E-02
↳plasma membrane bounded cell projection	2281	252	137.69	1.83	+	2.37E-16
↳cell projection	2499	275	150.85	1.82	+	5.42E-18
↳neuron projection	1516	184	91.51	2.01	+	1.20E-14
extracellular exosome	80	25	4.83	5.18	+	1.06E-06
↳extracellular vesicle	88	25	5.31	4.71	+	5.43E-06
↳extracellular organelle	105	25	6.34	3.94	+	1.06E-04
↳vesicle	2039	196	123.08	1.59	+	9.41E-07
stress fiber	85	26	5.13	5.07	+	7.14E-07
↳contractile actin filament bundle	85	26	5.13	5.07	+	7.14E-07
↳actin filament bundle	94	31	5.67	5.46	+	3.13E-09
↳actin cytoskeleton	497	88	30.00	2.93	+	7.49E-14
↳actomyosin	96	31	5.79	5.35	+	4.92E-09
P-body	72	22	4.35	5.06	+	1.54E-05
catalytic step 2 spliceosome	83	25	5.01	4.99	+	2.00E-06
↳spliceosomal complex	197	49	11.89	4.12	+	1.62E-11
↳nucleus	6824	609	411.92	1.48	+	1.54E-24
basement membrane	109	32	6.58	4.86	+	1.80E-08
↳collagen-containing extracellular matrix	366	81	22.09	3.67	+	1.50E-17
↳extracellular matrix	481	83	29.03	2.86	+	2.18E-12
vesicle coat	53	15	3.20	4.69	+	7.32E-03
↳coated vesicle membrane	76	18	4.59	3.92	+	7.40E-03
↳vesicle membrane	366	45	22.09	2.04	+	4.89E-02
↳organelle membrane	2118	246	127.85	1.92	+	2.19E-18
↳cytoplasmic vesicle	1905	172	114.99	1.50	+	7.13E-04
↳intracellular vesicle	1910	172	115.29	1.49	+	7.39E-04
↳whole membrane	1152	143	69.54	2.06	+	1.92E-11
↳coated vesicle	188	33	11.35	2.91	+	5.47E-04
↳bounding membrane of organelle	1098	115	66.28	1.74	+	1.03E-04
↳membrane coat	95	20	5.73	3.49	+	1.05E-02
↳coated membrane	95	20	5.73	3.49	+	1.05E-02
filopodium	93	25	5.61	4.45	+	1.39E-05
↳actin-based cell projection	216	43	13.04	3.30	+	3.49E-07
cytosolic large ribosomal subunit	68	18	4.10	4.39	+	1.93E-03
↳large ribosomal subunit	129	31	7.79	3.98	+	2.48E-06
nuclear matrix	88	22	5.31	4.14	+	3.10E-04

↳nuclear periphery	113	27	6.82	3.96	+	3.03E-05
↳nuclear lumen	3899	358	235.36	1.52	+	1.23E-12
mitochondrial ribosome	90	22	5.43	4.05	+	4.32E-04
↳organellar ribosome	90	22	5.43	4.05	+	4.32E-04
T-tubule	70	17	4.23	4.02	+	1.01E-02
↳sarcolemma	162	37	9.78	3.78	+	2.34E-07
cortical actin cytoskeleton	99	24	5.98	4.02	+	1.45E-04
↳cortical cytoskeleton	130	31	7.85	3.95	+	2.90E-06
↳cell cortex	316	64	19.07	3.36	+	7.16E-12
clathrin-coated pit	67	16	4.04	3.96	+	2.24E-02
↳plasma membrane region	1218	135	73.52	1.84	+	2.08E-07
endoplasmic reticulum-Golgi intermediate compartment	72	17	4.35	3.91	+	1.39E-02
growth cone	205	48	12.37	3.88	+	2.07E-10
↳site of polarized growth	213	49	12.86	3.81	+	2.04E-10
↳distal axon	379	60	22.88	2.62	+	6.23E-07
↳axon	715	103	43.16	2.39	+	3.64E-11
focal adhesion	156	36	9.42	3.82	+	3.22E-07
↳cell-substrate junction	168	38	10.14	3.75	+	1.69E-07
ruffle	149	33	8.99	3.67	+	4.38E-06
↳cell leading edge	389	73	23.48	3.11	+	2.75E-12
brush border	137	30	8.27	3.63	+	2.82E-05
↳cluster of actin-based cell projections	195	35	11.77	2.97	+	1.53E-04
microvillus	96	20	5.79	3.45	+	1.20E-02
peroxisome	144	30	8.69	3.45	+	7.39E-05
↳microbody	144	30	8.69	3.45	+	7.39E-05
U2-type spliceosomal complex	87	18	5.25	3.43	+	3.63E-02
mitochondrial protein complex	260	52	15.69	3.31	+	4.00E-09
lamellipodium	166	33	10.02	3.29	+	4.31E-05
plasma membrane raft	127	23	7.67	3.00	+	1.97E-02
↳membrane raft	372	56	22.46	2.49	+	8.43E-06
↳membrane microdomain	373	57	22.52	2.53	+	3.98E-06
↳membrane region	386	60	23.30	2.58	+	9.13E-07
postsynaptic density	396	70	23.90	2.93	+	1.37E-10
↳asymmetric synapse	400	71	24.15	2.94	+	7.62E-11
↳neuron to neuron synapse	427	74	25.78	2.87	+	6.44E-11
↳postsynaptic specialization	435	71	26.26	2.70	+	2.80E-09
dendritic spine	193	34	11.65	2.92	+	3.40E-04
↳dendrite	705	106	42.56	2.49	+	1.50E-12
↳dendritic tree	708	106	42.74	2.48	+	1.75E-12
↳somatodendritic compartment	1028	142	62.05	2.29	+	1.09E-14
↳neuron spine	199	35	12.01	2.91	+	2.35E-04
perinuclear region of cytoplasm	657	113	39.66	2.85	+	1.53E-17

nuclear speck	315	54	19.01	2.84	+	2.56E-07
↳nuclear body	682	83	41.17	2.02	+	2.55E-05
↳nucleoplasm	3324	302	200.65	1.51	+	2.01E-09
endocytic vesicle	191	32	11.53	2.78	+	2.00E-03
mitochondrial inner membrane	438	71	26.44	2.69	+	3.66E-09
↳mitochondrial membrane	617	95	37.24	2.55	+	1.09E-11
↳mitochondrial envelope	663	101	40.02	2.52	+	3.03E-12
↳organelle envelope	1069	158	64.53	2.45	+	2.28E-19
↳envelope	1070	158	64.59	2.45	+	2.42E-19
↳organelle inner membrane	482	74	29.10	2.54	+	1.65E-08
mitochondrial outer membrane	174	28	10.50	2.67	+	2.73E-02
↳organelle outer membrane	192	31	11.59	2.67	+	8.57E-03
↳outer membrane	192	31	11.59	2.67	+	8.57E-03
sarcomere	187	30	11.29	2.66	+	1.32E-02
↳myofibril	210	34	12.68	2.68	+	2.70E-03
↳contractile fiber	224	38	13.52	2.81	+	1.60E-04
↳supramolecular fiber	902	137	54.45	2.52	+	2.07E-17
↳supramolecular polymer	909	138	54.87	2.52	+	2.33E-17
microtubule	420	64	25.35	2.52	+	6.65E-07
↳microtubule cytoskeleton	1173	119	70.81	1.68	+	2.56E-04
↳polymeric cytoskeletal fiber	682	101	41.17	2.45	+	1.48E-11
glutamatergic synapse	508	73	30.66	2.38	+	3.70E-07
neuronal cell body	710	100	42.86	2.33	+	3.19E-10
↳cell body	801	115	48.35	2.38	+	1.28E-12
nuclear envelope	423	59	25.53	2.31	+	5.85E-05
cell projection membrane	295	41	17.81	2.30	+	6.10E-03
apical part of cell	429	51	25.90	1.97	+	3.25E-02
nuclear outer membrane-endoplasmic reticulum membrane network	566	64	34.17	1.87	+	1.03E-02
nucleolus	808	85	48.77	1.74	+	4.40E-03
integral component of plasma membrane	1499	34	90.49	.38	-	2.35E-08
↳intrinsic component of plasma membrane	1576	37	95.13	.39	-	2.22E-08
↳intrinsic component of membrane	6027	157	363.81	.43	-	6.06E-38
↳integral component of membrane	5854	148	353.37	.42	-	1.56E-38
Unclassified	1476	27	89.10	.30	-	0.00E00

About | Release Information | Contact Us | System Requirements | Privacy Policy | Disclaimer

© Copyright 2020 Paul Thomas All Rights Reserved.

LOGIN REGISTER CONTACT US

Home | About | PANTHER Data | PANTHER Tools | PANTHER Services | Workspace | Downloads | Help/Tutorial

PANTHER 15.0 released!

Analysis Summary: Please report in publication

Analysis Type: PANTHER Overrepresentation Test (Released 20200407)

Annotation Version and Release Date: GO Ontology database Released 2020-02-21

Analyzed List: upload_1 (Mus musculus) Change

Reference List: Mus musculus (all genes in database) Change

Annotation Data Set: GO biological process complete

Test Type: Fisher's Exact Binomial

Correction: Calculate False Discovery Rate Use the Bonferroni correction for multiple testing No correction

Results

	Reference list	upload_1
Uniquely Mapped IDS:	22265 out of 22265	1237 out of 1237
Unmapped IDs:	0	0
Multiple mapping information:	0	0

Bonferroni count: 8894

Export Table XML with user input ids JSON with user input ids

Displaying only results for Bonferroni-corrected for P < 0.05, click here to display all results

	Mus musculus (REF)	upload 1 (Hierarchy NEW!)				
GO biological process complete	#	#	expected	Fold Enrichment	+/-	P value
fatty acid beta-oxidation using acyl-CoA dehydrogenase	10	9	.56	16.20	+	1.53E-03
↳fatty acid beta-oxidation	49	25	2.72	9.18	+	2.20E-10
↳fatty acid oxidation	69	28	3.83	7.30	+	5.71E-10
↳fatty acid metabolic process	309	62	17.17	3.61	+	4.64E-12
↳monocarboxylic acid metabolic process	467	100	25.95	3.85	+	4.44E-23
↳carboxylic acid metabolic process	761	188	42.28	4.45	+	4.41E-55
↳oxoacid metabolic process	803	190	44.61	4.26	+	3.04E-53
↳organic acid metabolic process	829	193	46.06	4.19	+	3.08E-53
↳cellular metabolic process	6331	699	351.74	1.99	+	1.06E-84
↳metabolic process	7222	755	401.24	1.88	+	6.05E-85
↳cellular process	14059	1050	781.09	1.34	+	1.46E-57
↳small molecule metabolic process	1432	250	79.56	3.14	+	7.11E-50
↳organic substance metabolic process	6701	718	372.29	1.93	+	1.18E-82
↳cellular lipid metabolic process	801	84	44.50	1.89	+	1.75E-03
↳lipid metabolic process	1063	105	59.06	1.78	+	7.40E-04
↳primary metabolic process	6269	672	348.29	1.93	+	4.44E-74
↳lipid oxidation	75	28	4.17	6.72	+	2.99E-09
↳lipid modification	182	35	10.11	3.46	+	2.89E-05

↳oxidation-reduction process	787	130	43.72	2.97	+	1.68E-21
↳fatty acid catabolic process	73	28	4.06	6.90	+	1.75E-09
↳cellular lipid catabolic process	164	32	9.11	3.51	+	9.99E-05
↳cellular catabolic process	1492	206	82.89	2.49	+	8.77E-27
↳catabolic process	1730	237	96.12	2.47	+	3.63E-31
↳lipid catabolic process	259	44	14.39	3.06	+	9.01E-06
↳organic substance catabolic process	1433	213	79.61	2.68	+	5.44E-32
↳monocarboxylic acid catabolic process	93	35	5.17	6.77	+	2.75E-12
↳carboxylic acid catabolic process	194	62	10.78	5.75	+	1.16E-20
↳organic acid catabolic process	194	62	10.78	5.75	+	1.16E-20
↳small molecule catabolic process	290	80	16.11	4.97	+	6.88E-24
positive regulation of establishment of protein localization to telomere	10	8	.56	14.40	+	1.40E-02
↳positive regulation of establishment of protein localization	462	66	25.67	2.57	+	6.41E-07
↳positive regulation of biological process	6100	479	338.90	1.41	+	5.10E-13
↳regulation of biological process	11534	748	640.81	1.17	+	2.42E-05
↳biological regulation	12152	801	675.14	1.19	+	1.72E-08
↳regulation of establishment of protein localization	771	98	42.84	2.29	+	9.12E-09
↳regulation of protein localization	1075	136	59.72	2.28	+	2.67E-13
↳regulation of localization	2886	256	160.34	1.60	+	1.91E-09
↳regulation of cellular protein localization	560	87	31.11	2.80	+	9.66E-12
↳regulation of cellular localization	837	108	46.50	2.32	+	2.33E-10
↳positive regulation of cellular protein localization	308	51	17.11	2.98	+	9.52E-07
↳regulation of establishment of protein localization to telomere	11	8	.61	13.09	+	2.30E-02
↳regulation of establishment of protein localization to chromosome	12	8	.67	12.00	+	3.65E-02
viral translation	14	10	.78	12.86	+	1.39E-03
↳viral gene expression	20	11	1.11	9.90	+	2.33E-03
↳viral process	131	28	7.28	3.85	+	1.51E-04
↳symbiotic process	228	45	12.67	3.55	+	7.90E-08
↳interspecies interaction between organisms	1474	127	81.89	1.55	+	2.80E-02
tricarboxylic acid metabolic process	15	10	.83	12.00	+	2.21E-03
dicarboxylic acid biosynthetic process	12	8	.67	12.00	+	3.65E-02
↳dicarboxylic acid metabolic process	81	35	4.50	7.78	+	8.91E-14
↳carboxylic acid biosynthetic process	224	44	12.45	3.54	+	1.54E-07
↳organic acid biosynthetic process	225	45	12.50	3.60	+	5.36E-08
↳organic substance biosynthetic process	2079	273	115.51	2.36	+	5.28E-34
↳biosynthetic process	2161	283	120.06	2.36	+	2.65E-35
↳cellular biosynthetic process	1985	261	110.28	2.37	+	2.62E-32
↳small molecule biosynthetic process	446	70	24.78	2.82	+	3.00E-09
regulation of RNA binding	12	8	.67	12.00	+	3.65E-02
↳regulation of molecular function	2559	216	142.17	1.52	+	1.73E-05
negative regulation of mRNA splicing, via spliceosome	24	14	1.33	10.50	+	3.05E-05
↳regulation of mRNA splicing, via spliceosome	110	46	6.11	7.53	+	1.88E-18
↳regulation of RNA splicing	145	56	8.06	6.95	+	1.15E-21
↳regulation of gene expression	3827	334	212.62	1.57	+	7.60E-13
↳regulation of macromolecule metabolic process	5484	454	304.68	1.49	+	5.80E-16
↳regulation of metabolic process	5976	501	332.01	1.51	+	6.07E-20
↳regulation of nucleobase-containing compound metabolic process	3401	253	188.95	1.34	+	2.03E-02

↳regulation of primary metabolic process	5349	444	297.18	1.49	+	1.06E-15
↳regulation of nitrogen compound metabolic process	5198	426	288.79	1.48	+	1.19E-13
↳regulation of cellular metabolic process	5552	461	308.46	1.49	+	1.24E-16
↳regulation of cellular process	10826	698	601.47	1.16	+	8.92E-04
↳regulation of mRNA processing	149	56	8.28	6.76	+	3.39E-21
↳regulation of mRNA metabolic process	255	92	14.17	6.49	+	1.59E-35
↳negative regulation of RNA splicing	29	16	1.61	9.93	+	4.26E-06
↳negative regulation of macromolecule metabolic process	2494	238	138.56	1.72	+	1.13E-11
↳negative regulation of metabolic process	2766	262	153.67	1.70	+	5.81E-13
↳negative regulation of biological process	5169	436	287.18	1.52	+	2.00E-16
↳negative regulation of cellular metabolic process	2469	225	137.17	1.64	+	7.46E-09
↳negative regulation of cellular process	4632	383	257.34	1.49	+	3.62E-12
↳negative regulation of nitrogen compound metabolic process	2272	204	126.23	1.62	+	4.72E-07
↳negative regulation of gene expression	1643	166	91.28	1.82	+	8.36E-09
↳negative regulation of mRNA processing	32	16	1.78	9.00	+	1.28E-05
↳negative regulation of mRNA metabolic process	82	32	4.56	7.02	+	2.26E-11
stress granule assembly	16	9	.89	10.12	+	2.54E-02
↳cellular component assembly	1941	218	107.84	2.02	+	1.03E-17
↳cellular component organization	5045	421	280.29	1.50	+	8.72E-15
↳cellular component organization or biogenesis	5239	433	291.07	1.49	+	9.72E-15
↳cellular component biogenesis	2168	233	120.45	1.93	+	5.24E-17
↳organelle organization	3054	278	169.67	1.64	+	6.74E-12
tricarboxylic acid cycle	32	18	1.78	10.12	+	2.74E-07
↳aerobic respiration	66	20	3.67	5.45	+	1.34E-04
↳cellular respiration	129	31	7.17	4.33	+	2.27E-06
↳energy derivation by oxidation of organic compounds	191	43	10.61	4.05	+	5.38E-09
↳generation of precursor metabolites and energy	286	71	15.89	4.47	+	1.27E-18
alternative mRNA splicing, via spliceosome	18	10	1.00	10.00	+	7.69E-03
↳mRNA splicing, via spliceosome	196	49	10.89	4.50	+	3.98E-12
↳mRNA processing	405	101	22.50	4.49	+	6.72E-28
↳RNA processing	752	136	41.78	3.26	+	4.58E-26
↳RNA metabolic process	1232	194	68.45	2.83	+	1.13E-31
↳nucleic acid metabolic process	1767	215	98.17	2.19	+	1.95E-21
↳macromolecule metabolic process	5056	509	280.90	1.81	+	1.39E-40
↳nucleobase-containing compound metabolic process	2191	296	121.73	2.43	+	8.29E-40
↳cellular nitrogen compound metabolic process	2789	420	154.95	2.71	+	1.34E-74
↳nitrogen compound metabolic process	5718	613	317.68	1.93	+	7.09E-64
↳organic cyclic compound metabolic process	2616	346	145.34	2.38	+	2.56E-46
↳heterocycle metabolic process	2328	320	129.34	2.47	+	3.07E-45
↳cellular aromatic compound metabolic process	2402	325	133.45	2.44	+	8.86E-45
↳gene expression	1616	270	89.78	3.01	+	3.51E-51
↳mRNA metabolic process	531	127	29.50	4.30	+	2.08E-34
↳RNA splicing, via transesterification reactions with bulged adenosine as nucleophile	196	49	10.89	4.50	+	3.98E-12
↳RNA splicing, via transesterification reactions	196	49	10.89	4.50	+	3.98E-12
↳RNA splicing	318	85	17.67	4.81	+	9.33E-25
acyl-CoA biosynthetic process	20	11	1.11	9.90	+	2.33E-03
↳purine ribonucleotide biosynthetic process	115	26	6.39	4.07	+	1.83E-04

↳purine ribonucleotide metabolic process	256	63	14.22	4.43	+	4.74E-16
↳ribonucleotide metabolic process	266	64	14.78	4.33	+	6.41E-16
↳nucleotide metabolic process	352	74	19.56	3.78	+	6.52E-16
↳nucleoside phosphate metabolic process	362	74	20.11	3.68	+	2.57E-15
↳phosphate-containing compound metabolic process	1670	155	92.78	1.67	+	1.85E-05
↳phosphorus metabolic process	1692	156	94.00	1.66	+	2.34E-05
↳nucleobase-containing small molecule metabolic process	426	85	23.67	3.59	+	1.99E-17
↳organophosphate metabolic process	710	98	39.45	2.48	+	8.21E-11
↳ribose phosphate metabolic process	277	66	15.39	4.29	+	2.60E-16
↳carbohydrate derivative metabolic process	794	95	44.11	2.15	+	5.04E-07
↳purine nucleotide metabolic process	273	66	15.17	4.35	+	1.35E-16
↳purine-containing compound metabolic process	313	71	17.39	4.08	+	1.00E-16
↳organonitrogen compound metabolic process	4270	452	237.23	1.91	+	2.09E-39
↳ribonucleotide biosynthetic process	124	27	6.89	3.92	+	1.94E-04
↳nucleotide biosynthetic process	171	36	9.50	3.79	+	2.05E-06
↳nucleoside phosphate biosynthetic process	178	38	9.89	3.84	+	4.57E-07
↳organophosphate biosynthetic process	366	51	20.33	2.51	+	2.04E-04
↳nucleobase-containing compound biosynthetic process	653	68	36.28	1.87	+	3.29E-02
↳heterocycle biosynthetic process	718	77	39.89	1.93	+	2.27E-03
↳organic cyclic compound biosynthetic process	848	91	47.11	1.93	+	1.42E-04
↳aromatic compound biosynthetic process	734	74	40.78	1.81	+	3.47E-02
↳cellular nitrogen compound biosynthetic process	1118	193	62.11	3.11	+	2.27E-36
↳ribose phosphate biosynthetic process	131	29	7.28	3.98	+	4.27E-05
↳purine nucleotide biosynthetic process	125	28	6.94	4.03	+	6.28E-05
↳purine-containing compound biosynthetic process	130	29	7.22	4.02	+	3.68E-05
↳organonitrogen compound biosynthetic process	1070	192	59.45	3.23	+	3.01E-38
↳coenzyme biosynthetic process	99	23	5.50	4.18	+	7.72E-04
↳coenzyme metabolic process	204	53	11.33	4.68	+	6.13E-14
↳cofactor metabolic process	368	82	20.45	4.01	+	2.49E-19
↳cofactor biosynthetic process	163	33	9.06	3.64	+	2.67E-05
↳amide biosynthetic process	421	135	23.39	5.77	+	6.14E-49
↳cellular amide metabolic process	665	177	36.95	4.79	+	1.52E-55
↳ribonucleoside bisphosphate biosynthetic process	30	12	1.67	7.20	+	9.68E-03
↳ribonucleoside bisphosphate metabolic process	94	32	5.22	6.13	+	4.99E-10
↳nucleoside bisphosphate metabolic process	94	32	5.22	6.13	+	4.99E-10
↳nucleoside bisphosphate biosynthetic process	30	12	1.67	7.20	+	9.68E-03
↳thioester biosynthetic process	20	11	1.11	9.90	+	2.33E-03
↳thioester metabolic process	78	30	4.33	6.92	+	2.28E-10
↳sulfur compound metabolic process	267	62	14.83	4.18	+	1.10E-14
↳sulfur compound biosynthetic process	72	22	4.00	5.50	+	2.26E-05
↳purine nucleoside bisphosphate biosynthetic process	30	12	1.67	7.20	+	9.68E-03
↳purine nucleoside bisphosphate metabolic process	94	32	5.22	6.13	+	4.99E-10
↳acyl-CoA metabolic process	78	30	4.33	6.92	+	2.28E-10
aromatic amino acid family catabolic process	19	10	1.06	9.47	+	1.12E-02
↳aromatic compound catabolic process	292	56	16.22	3.45	+	6.39E-10
↳cellular amino acid catabolic process	90	32	5.00	6.40	+	1.86E-10

↳organonitrogen compound catabolic process	900	109	50.00	2.18	+	5.98E-09
↳cellular amino acid metabolic process	231	76	12.83	5.92	+	1.21E-26
↳organic cyclic compound catabolic process	323	64	17.95	3.57	+	2.63E-12
↳aromatic amino acid family metabolic process	34	13	1.89	6.88	+	5.23E-03
acetyl-CoA metabolic process	29	15	1.61	9.31	+	2.93E-05
glutamate metabolic process	24	12	1.33	9.00	+	1.47E-03
↳glutamine family amino acid metabolic process	57	20	3.17	6.32	+	1.69E-05
↳alpha-amino acid metabolic process	164	53	9.11	5.82	+	1.88E-17
translational elongation	30	15	1.67	9.00	+	4.18E-05
↳cellular macromolecule biosynthetic process	1193	161	66.28	2.43	+	5.38E-19
↳cellular macromolecule metabolic process	3958	371	219.90	1.69	+	2.72E-20
↳macromolecule biosynthetic process	1227	161	68.17	2.36	+	7.23E-18
↳translation	306	111	17.00	6.53	+	9.06E-44
↳cellular protein metabolic process	2844	279	158.01	1.77	+	4.13E-16
↳protein metabolic process	3420	318	190.01	1.67	+	7.90E-16
↳peptide biosynthetic process	326	116	18.11	6.40	+	3.22E-45
↳peptide metabolic process	448	134	24.89	5.38	+	1.17E-45
tRNA aminoacylation for protein translation	40	20	2.22	9.00	+	1.14E-07
↳tRNA aminoacylation	43	20	2.39	8.37	+	3.14E-07
↳amino acid activation	44	20	2.44	8.18	+	4.35E-07
↳tRNA metabolic process	165	28	9.17	3.05	+	9.79E-03
↳ncRNA metabolic process	416	55	23.11	2.38	+	2.70E-04
mRNA destabilization	32	16	1.78	9.00	+	1.28E-05
↳RNA destabilization	35	16	1.94	8.23	+	3.50E-05
↳regulation of RNA stability	109	42	6.06	6.94	+	1.46E-15
↳regulation of cellular catabolic process	698	122	38.78	3.15	+	7.56E-22
↳regulation of catabolic process	843	135	46.84	2.88	+	2.43E-21
↳posttranscriptional regulation of gene expression	427	133	23.72	5.61	+	5.98E-47
↳regulation of biological quality	3959	386	219.95	1.75	+	1.36E-24
↳positive regulation of cellular metabolic process	3270	258	181.67	1.42	+	1.32E-04
↳positive regulation of cellular process	5424	424	301.35	1.41	+	2.66E-10
↳positive regulation of metabolic process	3560	288	197.79	1.46	+	9.38E-07
↳positive regulation of nitrogen compound metabolic process	3086	240	171.45	1.40	+	1.65E-03
↳positive regulation of macromolecule metabolic process	3256	250	180.90	1.38	+	2.44E-03
↳positive regulation of cellular catabolic process	367	71	20.39	3.48	+	1.81E-13
↳positive regulation of catabolic process	436	75	24.22	3.10	+	6.12E-12
↳positive regulation of mRNA catabolic process	50	23	2.78	8.28	+	1.28E-08
↳positive regulation of mRNA metabolic process	93	34	5.17	6.58	+	1.47E-11
↳regulation of mRNA catabolic process	121	48	6.72	7.14	+	1.37E-18
↳regulation of mRNA stability	98	41	5.44	7.53	+	3.59E-16
↳negative regulation of translation	130	46	7.22	6.37	+	4.39E-16
↳negative regulation of cellular protein metabolic process	1004	124	55.78	2.22	+	4.88E-11
↳regulation of cellular protein metabolic process	2531	252	140.62	1.79	+	1.01E-14
↳regulation of protein metabolic process	2723	267	151.28	1.76	+	4.78E-15
↳negative regulation of protein metabolic process	1072	129	59.56	2.17	+	6.67E-11
↳negative regulation of cellular amide metabolic process	149	48	8.28	5.80	+	1.64E-15
↳regulation of cellular amide metabolic process	384	110	21.33	5.16	+	2.56E-35

↳regulation of translation	333	106	18.50	5.73	+	2.43E-37
↳regulation of cellular macromolecule biosynthetic process	3333	251	185.17	1.36	+	9.09E-03
↳regulation of macromolecule biosynthetic process	3419	261	189.95	1.37	+	1.77E-03
↳regulation of biosynthetic process	3644	291	202.45	1.44	+	2.93E-06
↳regulation of cellular biosynthetic process	3571	275	198.40	1.39	+	3.28E-04
production of miRNAs involved in gene silencing by miRNA	27	13	1.50	8.67	+	6.31E-04
↳gene silencing by miRNA	40	17	2.22	7.65	+	2.75E-05
↳posttranscriptional gene silencing by RNA	47	19	2.61	7.28	+	6.38E-06
↳gene silencing by RNA	77	24	4.28	5.61	+	3.08E-06
↳posttranscriptional gene silencing	48	20	2.67	7.50	+	1.49E-06
↳production of small RNA involved in gene silencing by RNA	29	13	1.61	8.07	+	1.21E-03
↳dsRNA processing	29	13	1.61	8.07	+	1.21E-03
translational initiation	55	25	3.06	8.18	+	1.68E-09
regulation of alternative mRNA splicing, via spliceosome	66	30	3.67	8.18	+	6.54E-12
NADH metabolic process	27	11	1.50	7.33	+	2.36E-02
actin filament capping	32	13	1.78	7.31	+	2.99E-03
↳negative regulation of actin filament depolymerization	38	15	2.11	7.10	+	5.09E-04
↳negative regulation of cytoskeleton organization	161	28	8.94	3.13	+	6.37E-03
↳regulation of cytoskeleton organization	559	69	31.06	2.22	+	8.83E-05
↳regulation of organelle organization	1270	142	70.56	2.01	+	6.21E-10
↳regulation of cellular component organization	2537	251	140.95	1.78	+	2.48E-14
↳negative regulation of cellular component organization	734	80	40.78	1.96	+	8.08E-04
↳negative regulation of protein depolymerization	72	17	4.00	4.25	+	3.02E-02
↳regulation of protein depolymerization	89	20	4.94	4.04	+	8.29E-03
↳regulation of protein-containing complex disassembly	121	27	6.72	4.02	+	1.24E-04
↳negative regulation of supramolecular fiber organization	154	30	8.56	3.51	+	2.94E-04
↳regulation of supramolecular fiber organization	368	51	20.45	2.49	+	2.31E-04
↳regulation of actin filament depolymerization	51	17	2.83	6.00	+	5.10E-04
↳regulation of actin polymerization or depolymerization	185	31	10.28	3.02	+	3.38E-03
↳regulation of actin filament length	188	31	10.44	2.97	+	4.60E-03
↳regulation of cellular component size	407	53	22.61	2.34	+	1.04E-03
↳regulation of anatomical structure size	571	74	31.72	2.33	+	2.51E-06
↳negative regulation of actin filament polymerization	58	18	3.22	5.59	+	5.16E-04
↳regulation of actin filament polymerization	168	29	9.33	3.11	+	4.65E-03
↳regulation of protein polymerization	224	39	12.45	3.13	+	4.38E-05
↳regulation of protein-containing complex assembly	434	57	24.11	2.36	+	2.49E-04
↳regulation of cellular component biogenesis	964	103	53.56	1.92	+	2.15E-05
↳negative regulation of protein polymerization	73	21	4.06	5.18	+	1.26E-04
↳negative regulation of protein-containing complex assembly	136	25	7.56	3.31	+	1.02E-02
alpha-amino acid catabolic process	75	30	4.17	7.20	+	9.89E-11
mRNA stabilization	41	16	2.28	7.02	+	2.10E-04
↳negative regulation of mRNA catabolic process	51	18	2.83	6.35	+	1.01E-04
↳negative regulation of RNA catabolic process	61	21	3.39	6.20	+	8.89E-06
↳negative regulation of cellular catabolic process	241	38	13.39	2.84	+	6.99E-04
↳negative regulation of catabolic process	301	42	16.72	2.51	+	4.37E-03
↳positive regulation of gene expression	1995	162	110.84	1.46	+	2.68E-02
↳RNA stabilization	48	18	2.67	6.75	+	4.66E-05
nuclear migration	31	12	1.72	6.97	+	1.28E-02

↳nucleus localization	36	12	2.00	6.00	+	4.55E-02
↳organelle localization	474	66	26.33	2.51	+	2.14E-06
↳cellular localization	2053	249	114.06	2.18	+	1.98E-25
↳localization	4864	399	270.23	1.48	+	2.00E-12
↳intracellular transport	1182	171	65.67	2.60	+	1.78E-23
↳transport	3572	322	198.45	1.62	+	3.81E-14
↳establishment of localization	3711	332	206.18	1.61	+	2.99E-14
↳establishment of organelle localization	331	52	18.39	2.83	+	3.30E-06
positive regulation of translation	123	44	6.83	6.44	+	2.03E-15
↳positive regulation of cellular amide metabolic process	149	48	8.28	5.80	+	1.64E-15
cortical actin cytoskeleton organization	42	15	2.33	6.43	+	1.47E-03
↳cortical cytoskeleton organization	59	18	3.28	5.49	+	6.41E-04
↳cytoskeleton organization	1070	118	59.45	1.98	+	1.96E-07
↳actin cytoskeleton organization	500	68	27.78	2.45	+	2.08E-06
↳actin filament-based process	555	72	30.83	2.34	+	4.48E-06
monosaccharide biosynthetic process	34	12	1.89	6.35	+	2.80E-02
↳monosaccharide metabolic process	156	28	8.67	3.23	+	3.65E-03
↳carbohydrate metabolic process	405	60	22.50	2.67	+	1.26E-06
cellular aldehyde metabolic process	52	18	2.89	6.23	+	1.29E-04
positive regulation of telomere maintenance via telomere lengthening	35	12	1.94	6.17	+	3.58E-02
↳positive regulation of organelle organization	596	82	33.11	2.48	+	2.13E-08
↳positive regulation of cellular component organization	1254	140	69.67	2.01	+	7.82E-10
↳regulation of telomere maintenance	81	19	4.50	4.22	+	8.87E-03
positive regulation of RNA splicing	50	17	2.78	6.12	+	4.02E-04
regulation of translational initiation	59	20	3.28	6.10	+	2.76E-05
alpha-amino acid biosynthetic process	51	17	2.83	6.00	+	5.10E-04
↳cellular amino acid biosynthetic process	55	18	3.06	5.89	+	2.63E-04
cytoplasmic translation	64	20	3.56	5.62	+	8.69E-05
ribosome assembly	62	19	3.44	5.52	+	2.63E-04
↳ribonucleoprotein complex biogenesis	392	62	21.78	2.85	+	5.17E-08
pyruvate metabolic process	69	20	3.83	5.22	+	2.49E-04
mitochondrial translation	52	15	2.89	5.19	+	1.41E-02
↳mitochondrial gene expression	81	21	4.50	4.67	+	5.75E-04
purine ribonucleoside triphosphate metabolic process	59	16	3.28	4.88	+	1.26E-02
↳purine nucleoside triphosphate metabolic process	69	17	3.83	4.43	+	1.84E-02
↳nucleoside triphosphate metabolic process	89	19	4.94	3.84	+	2.96E-02
↳ribonucleoside triphosphate metabolic process	63	17	3.50	4.86	+	6.33E-03
nuclear-transcribed mRNA catabolic process	94	25	5.22	4.79	+	2.12E-05
↳mRNA catabolic process	119	30	6.61	4.54	+	1.65E-06
↳RNA catabolic process	147	32	8.17	3.92	+	9.93E-06
↳nucleobase-containing compound catabolic process	234	37	13.00	2.85	+	9.70E-04
↳heterocycle catabolic process	278	52	15.45	3.37	+	1.16E-08
↳cellular nitrogen compound catabolic process	272	51	15.11	3.37	+	1.76E-08
↳cellular macromolecule catabolic process	762	86	42.34	2.03	+	5.70E-05
↳macromolecule catabolic process	858	97	47.67	2.03	+	4.15E-06
ribonucleoprotein complex assembly	163	43	9.06	4.75	+	5.12E-11
↳cellular protein-containing complex assembly	653	83	36.28	2.29	+	6.25E-07
	1003	127	55.72	2.28	+	2.95E-12

↳protein-containing complex assembly						
↳protein-containing complex subunit organization	1144	142	63.56	2.23	+	2.28E-13
↳ribonucleoprotein complex subunit organization	170	45	9.44	4.76	+	1.07E-11
mRNA transport	107	28	5.94	4.71	+	3.22E-06
↳RNA transport	150	37	8.33	4.44	+	2.22E-08
↳nucleic acid transport	150	37	8.33	4.44	+	2.22E-08
↳nucleobase-containing compound transport	183	38	10.17	3.74	+	9.18E-07
↳organic substance transport	1856	202	103.12	1.96	+	1.15E-14
↳nitrogen compound transport	1550	176	86.11	2.04	+	7.41E-14
↳establishment of RNA localization	152	37	8.44	4.38	+	3.10E-08
↳RNA localization	170	42	9.44	4.45	+	7.05E-10
↳macromolecule localization	2266	253	125.89	2.01	+	4.89E-21
endoplasmic reticulum organization	58	15	3.22	4.65	+	4.39E-02
↳endomembrane system organization	367	57	20.39	2.80	+	6.96E-07
antibiotic metabolic process	91	23	5.06	4.55	+	2.05E-04
↳drug metabolic process	412	83	22.89	3.63	+	3.54E-17
maintenance of protein location in cell	77	19	4.28	4.44	+	4.60E-03
↳maintenance of location in cell	103	24	5.72	4.19	+	3.88E-04
↳maintenance of location	173	32	9.61	3.33	+	3.01E-04
↳cellular protein localization	1423	175	79.06	2.21	+	6.72E-17
↳cellular macromolecule localization	1430	176	79.45	2.22	+	4.67E-17
↳protein localization	1963	218	109.06	2.00	+	4.39E-17
↳maintenance of protein location	108	23	6.00	3.83	+	2.96E-03
carbohydrate catabolic process	88	21	4.89	4.30	+	1.90E-03
protein folding	146	34	8.11	4.19	+	6.45E-07
positive regulation of viral process	82	19	4.56	4.17	+	1.04E-02
↳regulation of viral process	183	32	10.17	3.15	+	9.43E-04
↳regulation of symbiosis, encompassing mutualism through parasitism	200	36	11.11	3.24	+	7.96E-05
protein export from nucleus	91	21	5.06	4.15	+	3.06E-03
↳nuclear export	106	21	5.89	3.57	+	2.59E-02
↳nucleocytoplasmic transport	203	36	11.28	3.19	+	1.12E-04
↳nuclear transport	203	36	11.28	3.19	+	1.12E-04
↳intracellular protein transport	765	103	42.50	2.42	+	1.05E-10
↳protein transport	1246	154	69.23	2.22	+	1.03E-14
↳establishment of protein localization	1330	164	73.89	2.22	+	7.83E-16
↳peptide transport	1276	155	70.89	2.19	+	4.03E-14
↳amide transport	1301	156	72.28	2.16	+	8.27E-14
regulation of viral genome replication	93	20	5.17	3.87	+	1.50E-02
↳regulation of viral life cycle	142	25	7.89	3.17	+	2.03E-02
ATP metabolic process	170	36	9.44	3.81	+	1.78E-06
endoplasmic reticulum to Golgi vesicle-mediated transport	105	22	5.83	3.77	+	6.73E-03
↳Golgi vesicle transport	250	46	13.89	3.31	+	3.73E-07
↳vesicle-mediated transport	1271	128	70.61	1.81	+	6.08E-06
protein stabilization	174	36	9.67	3.72	+	3.09E-06
↳regulation of protein stability	278	46	15.45	2.98	+	7.97E-06
cellular modified amino acid metabolic process	156	32	8.67	3.69	+	3.51E-05
Golgi organization	111	22	6.17	3.57	+	1.51E-02
cytosolic transport	132	26	7.33	3.55	+	1.97E-03

drug catabolic process	148	29	8.22	3.53	+	4.44E-04
regulation of nucleocytoplasmic transport	125	23	6.94	3.31	+	2.67E-02
↳regulation of intracellular transport	352	52	19.56	2.66	+	2.45E-05
↳regulation of transport	1972	183	109.56	1.67	+	4.35E-07
establishment of vesicle localization	142	26	7.89	3.30	+	6.72E-03
↳establishment of localization in cell	353	46	19.61	2.35	+	5.69E-03
↳vesicle localization	154	27	8.56	3.16	+	8.72E-03
response to calcium ion	127	23	7.06	3.26	+	3.37E-02
↳response to metal ion	270	46	15.00	3.07	+	3.47E-06
↳response to inorganic substance	417	64	23.17	2.76	+	1.09E-07
↳response to chemical	3484	315	193.56	1.63	+	7.19E-14
cellular carbohydrate metabolic process	146	26	8.11	3.21	+	1.06E-02
receptor-mediated endocytosis	138	24	7.67	3.13	+	3.88E-02
regulation of mitochondrion organization	140	24	7.78	3.09	+	4.81E-02
regulation of cell shape	158	27	8.78	3.08	+	1.35E-02
↳regulation of cell morphogenesis	537	74	29.83	2.48	+	1.91E-07
↳regulation of anatomical structure morphogenesis	1090	110	60.56	1.82	+	1.08E-04
↳regulation of developmental process	2684	211	149.12	1.41	+	5.30E-03
establishment of protein localization to organelle	302	51	16.78	3.04	+	5.12E-07
↳protein localization to organelle	622	89	34.56	2.58	+	3.00E-10
protein-containing complex localization	206	34	11.44	2.97	+	1.29E-03
protein localization to nucleus	172	28	9.56	2.93	+	2.01E-02
actin filament organization	241	39	13.39	2.91	+	2.56E-04
↳supramolecular fiber organization	465	63	25.83	2.44	+	1.15E-05
positive regulation of intracellular transport	198	32	11.00	2.91	+	4.50E-03
↳positive regulation of transport	1099	116	61.06	1.90	+	3.25E-06
cytoskeleton-dependent intracellular transport	186	29	10.33	2.81	+	2.85E-02
positive regulation of protein-containing complex assembly	221	34	12.28	2.77	+	5.50E-03
↳positive regulation of cellular component biogenesis	524	60	29.11	2.06	+	6.65E-03
positive regulation of cell morphogenesis involved in differentiation	189	29	10.50	2.76	+	3.77E-02
↳regulation of cell morphogenesis involved in differentiation	348	46	19.33	2.38	+	4.48E-03
↳regulation of cell development	1092	100	60.67	1.65	+	3.36E-02
regulation of organelle assembly	207	31	11.50	2.70	+	2.79E-02
negative regulation of apoptotic signaling pathway	232	33	12.89	2.56	+	4.15E-02
↳negative regulation of apoptotic process	906	100	50.34	1.99	+	7.21E-06
↳negative regulation of programmed cell death	926	100	51.45	1.94	+	2.55E-05
↳regulation of programmed cell death	1518	141	84.34	1.67	+	1.15E-04
↳regulation of cell death	1672	159	92.89	1.71	+	2.34E-06
↳negative regulation of cell death	1041	111	57.84	1.92	+	6.25E-06
↳regulation of apoptotic process	1493	140	82.95	1.69	+	6.54E-05
regulation of microtubule-based process	241	34	13.39	2.54	+	3.37E-02
positive regulation of protein transport	443	58	24.61	2.36	+	1.89E-04
↳regulation of protein transport	739	87	41.06	2.12	+	7.59E-06
↳regulation of peptide transport	781	88	43.39	2.03	+	3.41E-05
organic hydroxy compound metabolic process	421	49	23.39	2.09	+	4.67E-02
regulation of vesicle-mediated transport	598	69	33.22	2.08	+	7.49E-04
cellular response to nitrogen compound	512	57	28.45	2.00	+	3.26E-02
↳response to nitrogen compound	851	94	47.28	1.99	+	2.34E-05
↳cellular response to chemical stimulus	2359	217	131.06	1.66	+	1.01E-08

response to organic cyclic compound	621	67	34.50	1.94	+	1.22E-02
↳response to organic substance	2488	232	138.23	1.68	+	3.88E-10
response to organonitrogen compound	745	80	41.39	1.93	+	1.08E-03
response to cytokine	746	77	41.45	1.86	+	8.65E-03
regulation of plasma membrane bounded cell projection organization	781	78	43.39	1.80	+	2.23E-02
↳regulation of cell projection organization	790	78	43.89	1.78	+	3.58E-02
regulation of hydrolase activity	994	97	55.22	1.76	+	4.06E-03
↳regulation of catalytic activity	1881	155	104.50	1.48	+	2.39E-02
cellular homeostasis	857	83	47.61	1.74	+	4.00E-02
↳homeostatic process	1644	141	91.34	1.54	+	9.59E-03
cellular response to oxygen-containing compound	912	88	50.67	1.74	+	1.90E-02
↳response to oxygen-containing compound	1296	121	72.00	1.68	+	9.75E-04
response to endogenous stimulus	1126	107	62.56	1.71	+	3.26E-03
regulation of response to stress	1295	117	71.95	1.63	+	9.09E-03
positive regulation of molecular function	1477	128	82.06	1.56	+	2.14E-02
cellular response to stress	1446	124	80.34	1.54	+	4.14E-02
↳response to stress	3164	237	175.79	1.35	+	2.84E-02
cellular response to organic substance	1822	156	101.23	1.54	+	2.58E-03
regulation of multicellular organismal process	3223	247	179.06	1.38	+	3.36E-03
developmental process	5570	392	309.46	1.27	+	2.82E-03
Unclassified	1901	32	105.62	.30	-	0.00E00
G protein-coupled receptor signaling pathway	1851	16	102.84	.16	-	1.16E-22
↳signal transduction	4827	177	268.18	.66	-	1.31E-06
↳cell communication	5260	209	292.24	.72	-	1.77E-04
↳signaling	5138	193	285.46	.68	-	2.15E-06
sensory perception of chemical stimulus	1228	1	68.23	.01	-	2.84E-24
↳sensory perception	1642	13	91.23	.14	-	1.19E-20
↳nervous system process	2084	44	115.78	.38	-	1.14E-10

About | Release Information | Contact Us | System Requirements | Privacy Policy | Disclaimer

© Copyright 2020 Paul Thomas All Rights Reserved.

LIVER - MF

PANTHER - Compare lists to reference list

GENEONTOLOGY Unifying Biology — PANTHER Classification System

LOGIN REGISTER CONTACT US

Home | About | PANTHER Data | PANTHER Tools | PANTHER Services | Workspace | Downloads | Help/Tutorial

PANTHER 15.0 released!

Analysis Summary: Please report in publication

Analysis Type: PANTHER Overrepresentation Test (Released 20200407)

Annotation Version and Release Date: GO Ontology database Released 2020-02-21

Analyzed List: upload_1 (Mus musculus) Change

Reference List: Mus musculus (all genes in database) Change

Annotation Data Set: GO molecular function complete

Test Type: Fisher's Exact Binomial

Correction: Calculate False Discovery Rate Use the Bonferroni correction for multiple testing No correction

Results

	Reference list	upload_1
Uniquely Mapped IDS:	22265 out of 22265	1237 out of 1237
Unmapped IDs:	0	0
Multiple mapping information:	0	0

Bonferroni count: 2756

Export Table XML with user input ids JSON with user input ids

Displaying only results for Bonferroni-corrected for P < 0.05, click here to display all results

	Mus musculus (REF)	upload 1 (▽ Hierarchy NEW!)				
GO molecular function complete	#	#	expected	Fold Enrichment	+/-	P value
N6-methyladenosine-containing RNA binding	8	7	.44	15.75	+	1.34E-02
↳RNA binding	1086	329	60.34	5.45	+	1.79E-125
↳nucleic acid binding	3161	371	175.62	2.11	+	1.23E-39
↳organic cyclic compound binding	5175	629	287.51	2.19	+	8.23E-89
↳binding	13351	1089	741.76	1.47	+	9.87E-98
↳heterocyclic compound binding	5072	620	281.79	2.20	+	7.78E-88
acyl-CoA dehydrogenase activity	10	8	.56	14.40	+	4.32E-03
↳oxidoreductase activity, acting on the CH-CH group of donors	56	16	3.11	5.14	+	2.19E-03
↳oxidoreductase activity	791	113	43.95	2.57	+	1.94E-14
↳catalytic activity	5659	542	314.40	1.72	+	1.36E-38
RNA stem-loop binding	14	10	.78	12.86	+	4.32E-04
translation elongation factor activity	17	11	.94	11.65	+	2.12E-04
↳translation factor activity, RNA binding	77	39	4.28	9.12	+	4.69E-18
↳translation regulator activity, nucleic acid binding	95	45	5.28	8.53	+	3.13E-20
↳translation regulator activity	126	59	7.00	8.43	+	5.45E-27
mRNA 5'-UTR binding	24	14	1.33	10.50	+	9.44E-06
↳mRNA binding	272	118	15.11	7.81	+	7.46E-54
miRNA binding	30	17	1.67	10.20	+	2.75E-07
↳regulatory RNA binding	43	18	2.39	7.53	+	3.55E-06
aminoacyl-tRNA ligase activity	41	20	2.28	8.78	+	4.98E-08
↳ligase activity, forming carbon-oxygen bonds	41	20	2.28	8.78	+	4.98E-08
↳ligase activity	146	53	8.11	6.53	+	7.39E-20

↳catalytic activity, acting on a tRNA	113	21	6.28	3.34	+	1.92E-02
↳catalytic activity, acting on RNA	337	63	18.72	3.36	+	1.50E-11
translation initiation factor activity	48	23	2.67	8.62	+	2.04E-09
RNA helicase activity	53	25	2.94	8.49	+	2.71E-10
↳helicase activity	148	33	8.22	4.01	+	9.98E-07
↳ATPase activity, coupled	279	49	15.50	3.16	+	1.14E-07
↳ATPase activity	412	66	22.89	2.88	+	1.97E-09
↳nucleoside-triphosphatase activity	748	109	41.56	2.62	+	3.18E-14
↳pyrophosphatase activity	801	114	44.50	2.56	+	2.53E-14
↳hydrolase activity, acting on acid anhydrides, in phosphorus-containing anhydrides	804	114	44.67	2.55	+	2.88E-14
↳hydrolase activity, acting on acid anhydrides	804	114	44.67	2.55	+	2.88E-14
↳hydrolase activity	2449	231	136.06	1.70	+	3.93E-11
poly(A) binding	24	11	1.33	8.25	+	2.93E-03
↳poly-purine tract binding	32	16	1.78	9.00	+	3.96E-06
↳single-stranded RNA binding	89	36	4.94	7.28	+	5.09E-14
double-stranded RNA binding	82	37	4.56	8.12	+	1.03E-15
acid-thiol ligase activity	27	12	1.50	8.00	+	1.23E-03
↳ligase activity, forming carbon-sulfur bonds	38	14	2.11	6.63	+	8.64E-04
mRNA 3'-UTR binding	87	37	4.83	7.65	+	4.88E-15
poly(U) RNA binding	26	11	1.44	7.62	+	5.45E-03
↳poly-pyrimidine tract binding	30	13	1.67	7.80	+	5.13E-04
NAD binding	61	25	3.39	7.38	+	3.25E-09
↳coenzyme binding	288	79	16.00	4.94	+	6.31E-24
↳cofactor binding	531	105	29.50	3.56	+	1.73E-22
↳nucleotide binding	2030	301	112.78	2.67	+	1.41E-48
↳small molecule binding	2421	354	134.51	2.63	+	7.77E-58
↳nucleoside phosphate binding	2030	301	112.78	2.67	+	1.41E-48
oxidoreductase activity, acting on the CH-NH group of donors	27	11	1.50	7.33	+	7.31E-03
ribosome binding	64	26	3.56	7.31	+	1.36E-09
↳ribonucleoprotein complex binding	146	45	8.11	5.55	+	2.79E-14
↳protein-containing complex binding	1451	230	80.61	2.85	+	1.41E-39
rRNA binding	70	28	3.89	7.20	+	2.35E-10
ADP binding	40	16	2.22	7.20	+	4.91E-05
↳adenyl ribonucleotide binding	1453	203	80.73	2.51	+	1.93E-27
↳adenyl nucleotide binding	1464	207	81.34	2.54	+	9.78E-29
↳purine nucleotide binding	1798	252	99.89	2.52	+	1.28E-35
↳purine ribonucleotide binding	1786	248	99.23	2.50	+	2.58E-34
↳ribonucleotide binding	1802	251	100.12	2.51	+	5.75E-35
↳carbohydrate derivative binding	2123	273	117.95	2.31	+	5.69E-33
↳anion binding	2703	368	150.17	2.45	+	1.79E-53
↳ion binding	5485	534	304.74	1.75	+	7.81E-40
pre-mRNA binding	33	13	1.83	7.09	+	1.23E-03
oxidoreductase activity, acting on the aldehyde or oxo group of donors, NAD or NADP as acceptor	47	18	2.61	6.89	+	1.10E-05
↳oxidoreductase activity, acting on the aldehyde or oxo group of donors	56	19	3.11	6.11	+	2.09E-05
Ran GTPase binding	34	12	1.89	6.35	+	8.68E-03
↳Ras GTPase binding	399	54	22.17	2.44	+	6.84E-05
↳small GTPase binding	416	55	23.11	2.38	+	8.35E-05
↳GTPase binding	515	69	28.61	2.41	+	1.02E-06
↳enzyme binding	2331	273	129.51	2.11	+	7.59E-27
↳protein binding	9112	791	506.24	1.56	+	4.64E-53
hydro-lyase activity	52	17	2.89	5.88	+	1.99E-04

↳carbon-oxygen lyase activity	62	18	3.44	5.23	+	3.71E-04
↳lyase activity	184	38	10.22	3.72	+	3.26E-07
flavin adenine dinucleotide binding	82	25	4.56	5.49	+	6.06E-07
unfolded protein binding	92	28	5.11	5.48	+	5.25E-08
structural constituent of ribosome	161	48	8.94	5.37	+	6.95E-15
↳structural molecule activity	576	124	32.00	3.87	+	3.82E-30
structural constituent of cytoskeleton	64	19	3.56	5.34	+	1.24E-04
amino acid binding	71	21	3.94	5.32	+	2.60E-05
↳carboxylic acid binding	223	44	12.39	3.55	+	4.20E-08
↳organic acid binding	237	45	13.17	3.42	+	7.58E-08
tRNA binding	60	17	3.33	5.10	+	1.10E-03
NADP binding	47	13	2.61	4.98	+	3.14E-02
pyridoxal phosphate binding	51	14	2.83	4.94	+	1.57E-02
↳vitamin B6 binding	52	14	2.89	4.85	+	1.89E-02
↳drug binding	1644	229	91.34	2.51	+	1.94E-31
↳vitamin binding	132	26	7.33	3.55	+	6.11E-04
actin filament binding	201	53	11.17	4.75	+	1.10E-14
↳actin binding	426	85	23.67	3.59	+	6.16E-18
↳cytoskeletal protein binding	976	151	54.22	2.78	+	2.13E-23
heat shock protein binding	140	33	7.78	4.24	+	2.89E-07
modified amino acid binding	100	22	5.56	3.96	+	1.02E-03
chaperone binding	101	20	5.61	3.56	+	1.38E-02
oxidoreductase activity, acting on the CH-OH group of donors, NAD or NADP as acceptor	141	27	7.83	3.45	+	5.90E-04
↳oxidoreductase activity, acting on CH-OH group of donors	149	29	8.28	3.50	+	1.56E-04
single-stranded DNA binding	111	21	6.17	3.41	+	1.51E-02
extracellular matrix structural constituent	143	27	7.94	3.40	+	7.54E-04
ion channel binding	140	23	7.78	2.96	+	4.27E-02
magnesium ion binding	197	32	10.94	2.92	+	1.26E-03
↳metal ion binding	3499	271	194.40	1.39	+	7.88E-05
↳cation binding	3598	282	199.90	1.41	+	1.13E-05
ubiquitin protein ligase binding	308	48	17.11	2.81	+	6.12E-06
↳ubiquitin-like protein ligase binding	323	50	17.95	2.79	+	3.47E-06
GTP binding	355	54	19.72	2.74	+	1.89E-06
↳purine ribonucleoside triphosphate binding	1713	235	95.17	2.47	+	2.14E-31
↳purine ribonucleoside binding	362	56	20.11	2.78	+	3.61E-07
↳ribonucleoside binding	365	57	20.28	2.81	+	1.79E-07
↳nucleoside binding	375	58	20.83	2.78	+	1.70E-07
↳purine nucleoside binding	366	56	20.33	2.75	+	7.96E-07
↳guanyl ribonucleotide binding	379	57	21.06	2.71	+	7.69E-07
↳guanyl nucleotide binding	379	57	21.06	2.71	+	7.69E-07
protein C-terminus binding	231	35	12.83	2.73	+	1.61E-03
amide binding	378	54	21.00	2.57	+	1.37E-05
ATP binding	1390	191	77.23	2.47	+	1.01E-24
microtubule binding	247	33	13.72	2.40	+	4.78E-02
↳tubulin binding	346	44	19.22	2.29	+	5.12E-03
phospholipid binding	427	57	23.72	2.40	+	3.52E-05
↳lipid binding	763	80	42.39	1.89	+	1.01E-03
sulfur compound binding	255	34	14.17	2.40	+	3.54E-02
GTPase activity	294	37	16.33	2.27	+	4.56E-02
protein domain specific binding	786	91	43.67	2.08	+	1.67E-06
protein kinase binding	727	81	40.39	2.01	+	8.75E-05
↳kinase binding	813	85	45.17	1.88	+	4.42E-04
protein homodimerization activity	648	71	36.00	1.97	+	1.07E-03

↳identical protein binding	1983	253	110.17	2.30	+	1.04E-29
↳protein dimerization activity	945	89	52.50	1.70	+	1.24E-02
enzyme regulator activity	989	90	54.95	1.64	+	3.80E-02
Unclassified	2116	32	117.56	.27	-	0.00E00
DNA-binding transcription activator activity, RNA polymerase II-specific	472	5	26.22	.19	-	3.49E-03
↳DNA-binding transcription activator activity	475	5	26.39	.19	-	3.59E-03
↳DNA-binding transcription factor activity	1066	20	59.22	.34	-	2.18E-05
↳DNA-binding transcription factor activity, RNA polymerase II-specific	814	13	45.22	.29	-	1.28E-04
receptor ligand activity	488	5	27.11	.18	-	1.76E-03
↳signaling receptor activator activity	493	5	27.39	.18	-	1.23E-03
↳receptor regulator activity	534	6	29.67	.20	-	1.16E-03
odorant binding	473	2	26.28	.08	-	1.19E-05
G protein-coupled receptor activity	750	1	41.67	.02	-	3.38E-13
↳transmembrane signaling receptor activity	2139	3	118.84	.03	-	2.70E-44
↳signaling receptor activity	2339	10	129.95	.08	-	3.34E-40
↳molecular transducer activity	2344	10	130.23	.08	-	3.47E-40

About | Release Information | Contact Us | System Requirements | Privacy Policy | Disclaimer
© Copyright 2020 Paul Thomas All Rights Reserved.

GENEONTOLOGY Unifying Biology

PANTHER Classification System

LOGIN REGISTER CONTACT US

Home | About | PANTHER Data | PANTHER Tools | PANTHER Services | Workspace | Downloads | Help/Tutorial

PANTHER 15.0 released!

Analysis Summary: Please report in publication

Analysis Type: PANTHER Overrepresentation Test (Released 20200407)

Annotation Version and Release Date: GO Ontology database Released 2020-02-21

Analyzed List: upload_1 (Mus musculus) Change

Reference List: Mus musculus (all genes in database) Change

Annotation Data Set: GO cellular component complete

Test Type: (•) Fisher's Exact ( ) Binomial

Correction: ( ) Calculate False Discovery Rate (•) Use the Bonferroni correction for multiple testing ( ) No correction

Results

	Reference list	upload_1
Uniquely Mapped IDS:	22265 out of 22265	1237 out of 1237
Unmapped IDs:	0	0
Multiple mapping information:	0	0

Bonferroni count: 1452

Export Table | XML with user input ids | JSON with user input ids

Displaying only results for Bonferroni-corrected for P < 0.05, click here to display all results

	Mus musculus (REF)	upload_1 (▽ Hierarchy NEW!)				
GO cellular component complete	#	#	expected	Fold Enrichment	+/-	P value
eukaryotic translation initiation factor 3 complex, eIF3m	8	7	.44	15.75	+	7.08E-03
↳eukaryotic translation initiation factor 3 complex	15	12	.83	14.40	+	5.18E-06
↳cytoplasm	10945	1073	608.08	1.76	+	9.25E-160
↳cellular anatomical entity	18638	1197	1035.49	1.16	+	8.12E-44
↳intracellular	13691	1155	760.65	1.52	+	1.73E-139
↳protein-containing complex	5309	588	294.96	1.99	+	1.31E-65
chaperonin-containing T-complex	10	8	.56	14.40	+	2.28E-03
↳chaperone complex	22	14	1.22	11.45	+	2.16E-06
↳cytosol	3533	514	196.29	2.62	+	1.09E-92
aminoacyl-tRNA synthetase multienzyme complex	12	9	.67	13.50	+	7.23E-04
zona pellucida receptor complex	13	9	.72	12.46	+	1.17E-03
endoplasmic reticulum chaperone complex	12	8	.67	12.00	+	5.97E-03
↳endoplasmic reticulum	1639	175	91.06	1.92	+	3.21E-12
↳endomembrane system						

	3884	351	215.79	1.63	+	5.41E-17
↳intracellular membrane-bounded organelle	10291	916	571.75	1.60	+	3.24E-80
↳intracellular organelle	11954	1033	664.14	1.56	+	1.91E-100
↳organelle	12303	1037	683.53	1.52	+	1.86E-93
↳membrane-bounded organelle	11048	950	613.81	1.55	+	3.44E-78
messenger ribonucleoprotein complex	13	8	.72	11.08	+	9.19E-03
↳ribonucleoprotein complex	703	182	39.06	4.66	+	1.60E-56
proton-transporting two-sector ATPase complex, catalytic domain	14	8	.78	10.29	+	1.38E-02
cytoplasmic stress granule	66	35	3.67	9.55	+	9.26E-17
↳cytoplasmic ribonucleoprotein granule	206	73	11.44	6.38	+	6.16E-28
↳ribonucleoprotein granule	217	80	12.06	6.64	+	8.37E-32
↳intracellular non-membrane-bounded organelle	4162	444	231.23	1.92	+	4.51E-40
↳non-membrane-bounded organelle	4181	445	232.29	1.92	+	6.15E-40
↳supramolecular complex	1197	196	66.50	2.95	+	6.23E-35
smooth endoplasmic reticulum	36	17	2.00	8.50	+	1.27E-06
mitochondrial nucleoid	47	22	2.61	8.43	+	4.87E-09
↳mitochondrial matrix	272	86	15.11	5.69	+	2.79E-30
↳intracellular organelle lumen	4311	438	239.51	1.83	+	3.06E-34
↳organelle lumen	4312	438	239.57	1.83	+	3.12E-34
↳membrane-enclosed lumen	4312	438	239.57	1.83	+	3.12E-34
↳mitochondrion	1803	304	100.17	3.03	+	1.76E-60
↳nucleoid	47	22	2.61	8.43	+	4.87E-09
polysomal ribosome	30	14	1.67	8.40	+	4.39E-05
↳polysome	73	35	4.06	8.63	+	1.11E-15
↳ribosome	234	69	13.00	5.31	+	2.15E-22
cytosolic small ribosomal subunit	46	20	2.56	7.83	+	1.33E-07
↳small ribosomal subunit	78	30	4.33	6.92	+	3.72E-11
↳ribosomal subunit	202	59	11.22	5.26	+	1.08E-18
↳cytosolic ribosome	117	37	6.50	5.69	+	6.05E-12
myelin sheath	213	88	11.83	7.44	+	2.52E-38
proteasome accessory complex	26	10	1.44	6.92	+	1.65E-02
↳proteasome complex	66	19	3.67	5.18	+	9.87E-05
↳endopeptidase complex	67	19	3.72	5.10	+	1.20E-04
↳peptidase complex	93	20	5.17	3.87	+	2.44E-03
↳catalytic complex	1341	119	74.50	1.60	+	2.29E-03
neuron projection cytoplasm	39	12	2.17	5.54	+	1.46E-02
↳cytoplasmic region	216	31	12.00	2.58	+	1.20E-02
↳plasma membrane bounded cell projection	2281	229	126.73	1.81	+	3.86E-14
↳cell projection	2499	249	138.84	1.79	+	2.45E-15
↳neuron projection	1516	166	84.23	1.97	+	2.14E-12
P-body	72	22	4.00	5.50	+	3.70E-06
catalytic step 2 spliceosome	83	25	4.61	5.42	+	3.95E-07
↳spliceosomal complex	197	48	10.94	4.39	+	3.02E-12

↳nucleus	6824	570	379.13	1.50	+	3.67E-25
intercalated disc	62	18	3.44	5.23	+	1.96E-04
↳cell-cell contact zone	85	20	4.72	4.24	+	7.26E-04
↳anchoring junction	619	72	34.39	2.09	+	4.62E-05
↳cell junction	2028	251	112.67	2.23	+	8.60E-28
stress fiber	85	23	4.72	4.87	+	1.14E-05
↳contractile actin filament bundle	85	23	4.72	4.87	+	1.14E-05
↳actin filament bundle	94	28	5.22	5.36	+	4.22E-08
↳actin cytoskeleton	497	81	27.61	2.93	+	9.99E-13
↳cytoskeleton	2135	220	118.62	1.85	+	1.54E-14
↳actomyosin	96	28	5.33	5.25	+	6.37E-08
cytosolic large ribosomal subunit	68	18	3.78	4.76	+	6.22E-04
↳large ribosomal subunit	129	31	7.17	4.33	+	3.71E-07
extracellular exosome	80	21	4.44	4.72	+	7.83E-05
↳extracellular vesicle	88	21	4.89	4.30	+	3.10E-04
↳extracellular organelle	105	21	5.83	3.60	+	3.71E-03
↳vesicle	2039	173	113.28	1.53	+	1.43E-04
nuclear matrix	88	22	4.89	4.50	+	7.95E-05
↳nuclear periphery	113	26	6.28	4.14	+	2.19E-05
↳nuclear lumen	3899	340	216.62	1.57	+	5.65E-14
vesicle coat	53	13	2.94	4.41	+	4.89E-02
↳coated vesicle membrane	76	16	4.22	3.79	+	3.28E-02
↳organelle membrane	2118	231	117.67	1.96	+	2.17E-18
↳cytoplasmic vesicle	1905	153	105.84	1.45	+	1.53E-02
↳intracellular vesicle	1910	153	106.12	1.44	+	1.96E-02
↳whole membrane	1152	125	64.00	1.95	+	2.52E-08
↳coated vesicle	188	30	10.44	2.87	+	2.08E-03
↳bounding membrane of organelle	1098	106	61.00	1.74	+	2.72E-04
↳membrane coat	95	18	5.28	3.41	+	3.58E-02
↳coated membrane	95	18	5.28	3.41	+	3.58E-02
filopodium	93	22	5.17	4.26	+	1.83E-04
↳actin-based cell projection	216	39	12.00	3.25	+	2.92E-06
mitochondrial ribosome	90	21	5.00	4.20	+	4.27E-04
↳organellar ribosome	90	21	5.00	4.20	+	4.27E-04
clathrin-coated pit	67	15	3.72	4.03	+	3.15E-02
↳plasma membrane region	1218	116	67.67	1.71	+	1.31E-04
endoplasmic reticulum-Golgi intermediate compartment	72	16	4.00	4.00	+	1.83E-02
cortical actin cytoskeleton	99	22	5.50	4.00	+	4.63E-04
↳cortical cytoskeleton	130	29	7.22	4.02	+	6.01E-06
↳cell cortex	316	62	17.56	3.53	+	1.88E-12
T-tubule	70	15	3.89	3.86	+	4.91E-02
↳sarcolemma	162	32	9.00	3.56	+	1.26E-05
brush border	137	29	7.61	3.81	+	1.66E-05

↳cluster of actin-based cell projections	195	33	10.83	3.05	+	1.92E-04
focal adhesion	156	33	8.67	3.81	+	1.68E-06
↳cell-substrate junction	168	35	9.33	3.75	+	7.55E-07
microvillus	96	20	5.33	3.75	+	3.73E-03
ruffle	149	31	8.28	3.74	+	7.47E-06
↳cell leading edge	389	69	21.61	3.19	+	3.93E-12
sarcoplasm	83	17	4.61	3.69	+	2.50E-02
peroxisome	144	29	8.00	3.62	+	4.31E-05
↳microbody	144	29	8.00	3.62	+	4.31E-05
mitochondrial protein complex	260	51	14.45	3.53	+	6.49E-10
U2-type spliceosomal complex	87	17	4.83	3.52	+	4.24E-02
growth cone	205	40	11.39	3.51	+	2.48E-07
↳site of polarized growth	213	41	11.83	3.46	+	2.11E-07
↳distal axon	379	51	21.06	2.42	+	1.08E-04
↳axon	715	90	39.72	2.27	+	2.43E-08
lamellipodium	166	32	9.22	3.47	+	2.10E-05
dendritic spine	193	32	10.72	2.98	+	4.44E-04
↳dendrite	705	97	39.17	2.48	+	2.17E-11
↳dendritic tree	708	97	39.34	2.47	+	2.67E-11
↳somatodendritic compartment	1028	129	57.11	2.26	+	5.64E-13
↳postsynapse	727	102	40.39	2.53	+	1.70E-12
↳synapse	1435	195	79.73	2.45	+	4.65E-25
↳neuron spine	199	33	11.06	2.98	+	2.91E-04
plasma membrane raft	127	21	7.06	2.98	+	4.77E-02
↳membrane raft	372	47	20.67	2.27	+	1.73E-03
↳membrane microdomain	373	48	20.72	2.32	+	1.01E-03
↳membrane region	386	51	21.45	2.38	+	1.52E-04
nuclear speck	315	52	17.50	2.97	+	1.12E-07
↳nuclear body	682	79	37.89	2.08	+	1.11E-05
↳nucleoplasm	3324	288	184.67	1.56	+	9.35E-11
mitochondrial inner membrane	438	69	24.33	2.84	+	6.22E-10
↳mitochondrial membrane	617	91	34.28	2.65	+	4.33E-12
↳mitochondrial envelope	663	95	36.83	2.58	+	5.30E-12
↳organelle envelope	1069	148	59.39	2.49	+	7.19E-19
↳envelope	1070	148	59.45	2.49	+	7.65E-19
↳organelle inner membrane	482	72	26.78	2.69	+	1.97E-09
collagen-containing extracellular matrix	366	56	20.33	2.75	+	4.20E-07
↳extracellular matrix	481	58	26.72	2.17	+	3.54E-04
endocytic vesicle	191	29	10.61	2.73	+	7.39E-03
postsynaptic density	396	60	22.00	2.73	+	1.09E-07
↳asymmetric synapse	400	61	22.22	2.74	+	6.44E-08
↳neuron to neuron synapse	427	63	23.72	2.66	+	8.05E-08
↳postsynaptic specialization	435	61	24.17	2.52	+	1.01E-06

perinuclear region of cytoplasm	657	99	36.50	2.71	+	5.69E-14
sarcomere	187	28	10.39	2.70	+	1.35E-02
↳myofibril	210	32	11.67	2.74	+	2.29E-03
↳contractile fiber	224	35	12.45	2.81	+	4.46E-04
↳supramolecular fiber	902	124	50.11	2.47	+	5.48E-15
↳supramolecular polymer	909	124	50.50	2.46	+	7.19E-15
organelle outer membrane	192	28	10.67	2.62	+	3.01E-02
↳outer membrane	192	28	10.67	2.62	+	3.01E-02
microtubule	420	59	23.33	2.53	+	1.87E-06
↳microtubule cytoskeleton	1173	110	65.17	1.69	+	6.30E-04
↳polymeric cytoskeletal fiber	682	91	37.89	2.40	+	7.47E-10
nuclear envelope	423	54	23.50	2.30	+	1.77E-04
cell projection membrane	295	37	16.39	2.26	+	2.52E-02
neuronal cell body	710	89	39.45	2.26	+	3.65E-08
↳cell body	801	104	44.50	2.34	+	7.08E-11
glutamatergic synapse	508	62	28.22	2.20	+	9.00E-05
endoplasmic reticulum membrane	540	57	30.00	1.90	+	2.25E-02
↳nuclear outer membrane-endoplasmic reticulum membrane network	566	62	31.45	1.97	+	2.75E-03
nucleolus	808	83	44.89	1.85	+	7.26E-04
Unclassified	1476	23	82.00	.28	-	0.00E00
integral component of plasma membrane	1499	23	83.28	.28	-	1.15E-11
↳intrinsic component of plasma membrane	1576	26	87.56	.30	-	2.04E-11
↳intrinsic component of membrane	6027	135	334.85	.40	-	3.17E-39
↳integral component of membrane	5854	127	325.24	.39	-	1.17E-39

© Copyright 2020 Paul Thomas All Rights Reserved.
