# Supplementary material for: The RNA-binding protein landscapes differ between mammalian organs and cultured cells
Source: Nat Commun. 2023 Apr 12;14:2074. doi: 10.1038/s41467-023-37494-w (PMC10097726; doi:10.1038/s41467-023-37494-w)
Supplement: Supplementary file 15 — Reporting Summary [file 41467_2023_37494_MOESM15_ESM.pdf]

nature portfolio

Corresponding author(s): Bruno Galy
Matthias W. Hentze

Last updated by author(s): Nov 10, 2022

Reporting Summary

Nature Portfolio wishes to improve the reproducibility of the work that we publish. This form provides structure for consistency and transparency in reporting. For further information on Nature Portfolio policies, see our Editorial Policies and the Editorial Policy Checklist.

Statistics

For all statistical analyses, confirm that the following items are present in the figure legend, table legend, main text, or Methods section.

n/a	Confirmed	
☐	☒	The exact sample size (*n*) for each experimental group/condition, given as a discrete number and unit of measurement
☐	☒	A statement on whether measurements were taken from distinct samples or whether the same sample was measured repeatedly
☐	☒	The statistical test(s) used AND whether they are one- or two-sided<br>*Only common tests should be described solely by name; describe more complex techniques in the Methods section.*
☐	☒	A description of all covariates tested
☐	☒	A description of any assumptions or corrections, such as tests of normality and adjustment for multiple comparisons
☐	☒	A full description of the statistical parameters including central tendency (e.g. means) or other basic estimates (e.g. regression coefficient) AND variation (e.g. standard deviation) or associated estimates of uncertainty (e.g. confidence intervals)
☐	☒	For null hypothesis testing, the test statistic (e.g. *F*, *t*, *r*) with confidence intervals, effect sizes, degrees of freedom and *P* value noted<br>*Give P values as exact values whenever suitable.*
☒	☐	For Bayesian analysis, information on the choice of priors and Markov chain Monte Carlo settings
☒	☐	For hierarchical and complex designs, identification of the appropriate level for tests and full reporting of outcomes
☐	☒	Estimates of effect sizes (e.g. Cohen's *d*, Pearson's *r*), indicating how they were calculated

Our web collection on statistics for biologists contains articles on many of the points above.

Software and code

Policy information about availability of computer code

Data collection

IsobarQuant, Mascot (v2.2.07) and MaxQuant (v1.6.17.0) were used to process the acquired raw mass spec data.

Data analysis

ex vivo eRIC, non-poly(A)RIC and total proteome analyses: The R programming language (ISBN 3-900051-07-0) was used to analyze the raw output data of IsobarQuant. Raw TMT reporter ion intensities were cleaned for batch effects using the ‘removeBatchEffects’ function of the limma package and further normalized using the vsn package. The replicate information was added as a factor in the design matrix given as an argument to the ‘lmFit’ function of limma. Pearson's correlations were calculated using the cor function in R.

Global assessment of protein-protein crosslinking: The raw output file of MaxQuant was processed using the R programming language (ISBN 3-900051-07-0). iBAQ values were cleaned for batch effects using the ‘removeBatchEffect’ function of the limma package.

Hit classification, GO and domain analysis: Mouse and human RNA interactome studies along with functional annotations were downloaded from the RBPbase v.0.2.0. The R package ‘ggupset’ was used for the visualization of overlaps between datasets. Fisher’s Exact with independent hypothesis weighting (IHW) for multiple hypothesis testing correction was used for overrepresentation analysis of protein domain information from MouseMine. GO-term enrichment analysis in Figure 2b were conducted with AmiGO 2 using the following parameters: analysis type: PANTHER overrepresentation test (Released 20200407); Annotation version and release date: GO ontology database Released 2020-02-21; reference list: Mus musculus (all genes in database); test type: Fisher’s exact with Bonferroni correction for multiple testing. Graphical representations were made with the ggplot2 R package. KEGG- and remaining GO-term enrichment analysis were performed in g:Profiler (https://biit.cs.ut.ee/gprofiler/gost) using the following parameters: organism: Mus musculus; statistical domain scope: only annotated genes; significance threshold: g:SCS threshold (tailor-made algorithm for multiple testing correction). PANTHER protein class analysis was performed using the PANTHER classification system v.16.0.

Analysis of nucleotide-binding domains and cofactors: InterPro domain and cofactor annotations were retrieved from the UniProt mouse database (release-2021_03).

PLA: To count the PLA signals per cell, the CellProfiler Software version 4.1.3 was used. The range of the signal spot size was set to 8 to 20 pixels and the range of the nuclear size (DAPI signal) was set to 100 to 400 pixels. In both instances, the global threshold strategy minimum cross-entropy was used. The threshold smoothing factor was 1.3488 for the PLA signals and 20 for the nuclei. The Alexa Fluor 488 signal was used as an outline of the cells. Clumped objects were separated by intensity and objects touching the border of the images were discarded. The PLA signal per cell was counted by combining the information of the cellular outline (Alexa Fluor 488) and the DAPI signal. The statistical analysis of these results was performed using Graphpad Prism version 9 (one-way ANOVA with Tukey post-hoc test).

For manuscripts utilizing custom algorithms or software that are central to the research but not yet described in published literature, software must be made available to editors and reviewers. We strongly encourage code deposition in a community repository (e.g. GitHub). See the Nature Portfolio guidelines for submitting code & software for further information.

Data

Policy information about availability of data

All manuscripts must include a data availability statement. This statement should provide the following information, where applicable:

- Accession codes, unique identifiers, or web links for publicly available datasets
- A description of any restrictions on data availability
- For clinical datasets or third party data, please ensure that the statement adheres to our policy

All data generated during this study are included in this published Article (and its Supplementary Information files). The mass spectrometry proteomics data have been deposited to the ProteomeXchange Consortium via the PRIDE73 partner repository with the dataset identifiers PXD032113 [http://www.ebi.ac.uk/pride/archive/projects/PXD032113] (ex vivo eRIC and total proteome of brain, kidney and liver), PXD038076 [http://www.ebi.ac.uk/pride/archive/projects/PXD038076] (non-poly(A)RIC of brain, kidney and liver), PXD038099 [http://www.ebi.ac.uk/pride/archive/projects/PXD038099] (comprehensive non-poly(A)RIC of liver), and PXD038100 [http://www.ebi.ac.uk/pride/archive/projects/PXD038100] (global assessment of protein-protein crosslinks in ex vivo eRIC eluates of kidney origin). Source data are provided with this paper. Access information to the previously published RBP-profiling studies and total proteome datasets used in this study is provided in Supplementary Data 11.

Human research participants

Policy information about studies involving human research participants and Sex and Gender in Research.

Reporting on sex and gender	NA
Population characteristics	NA
Recruitment	NA
Ethics oversight	NA

Note that full information on the approval of the study protocol must also be provided in the manuscript.

Field-specific reporting

Please select the one below that is the best fit for your research. If you are not sure, read the appropriate sections before making your selection.

☒ Life sciences ☐ Behavioural & social sciences ☐ Ecological, evolutionary & environmental sciences

For a reference copy of the document with all sections, see nature.com/documents/nr-reporting-summary-flat.pdf

Life sciences study design

All studies must disclose on these points even when the disclosure is negative.

Sample size	No sample size calculation was performed. Sample size was determined based on the minimum amount of captured RNA required. This was in turn defined based on preliminary pilot experiments using organs, as well as in previous results using cell lines.
Data exclusions	No data were excluded from the MS analyses presented in the manuscript (ex vivo eRIC, non-poly(A)RIC, total proteomes and global assessment of protein-protein crosslinks in ex vivo eRIC samples).<br><br>Samples with aberrant values relative to the rest of biologically independent replicates were excluded from the nucleic acid/protein quantifications depicted in Fig. 1b and c (2 samples, belonging to one out of four biologically independent experiments) and Fig. 3e (1 and 2 samples of kidney and liver origin, respectively, out of five biologically independent experiments).
Replication	ex vivo eRIC and non-poly(A)RIC of brain, kidney and liver: four irradiated samples per organ were first generated, each derived from the

	respective organs of a single mouse. To obtain sufficient material, we combined the eluates obtained from two mice, rendering two irradiated samples per tissue. Organ sections from four mice were pooled to generate one non-crosslinked eRIC control per organ studied.<br><br>Comprehensive non-poly(A)RIC of liver: we employed four irradiated samples, each derived from the liver of a single mouse. Organ sections from four mice were pooled to generate one non-crosslinked eRIC control.<br><br>Global assessment of protein-protein crosslinks in ex vivo eRIC samples: we employed 3 samples, each derived from the kidneys of a single mouse.<br><br>We used Principal Component Analysis (PCA) plots to visually inspect reproducibility of MS data. The employed differential abundance test (limma) takes into account reproducibility to calculate p-values. In the manuscript we show correlation of independent ex vivo eRIC experiments.
Randomization	Each sample of a particular experiment was included into the same single MS run. To further control for potential batch-effects, the batch was always part of the statistical test.
Blinding	The investigators conducting the MS data analysis were not involved in the experimental execution and were not aware of the expected outcome of the experiments.

Reporting for specific materials, systems and methods

We require information from authors about some types of materials, experimental systems and methods used in many studies. Here, indicate whether each material, system or method listed is relevant to your study. If you are not sure if a list item applies to your research, read the appropriate section before selecting a response.

Materials & experimental systems

n/a	Involved in the study
☐	☒ Antibodies
☒	☐ Eukaryotic cell lines
☒	☐ Palaeontology and archaeology
☐	☒ Animals and other organisms
☒	☐ Clinical data
☒	☐ Dual use research of concern

Methods

n/a	Involved in the study
☒	☐ ChIP-seq
☒	☐ Flow cytometry
☒	☐ MRI-based neuroimaging

Antibodies

Antibodies used	PRIMARY ANTIBODIES, WB: anti-ELAV-like protein 1 (ELAVL1)/Hu-antigen R (HuR) (Proteintech, 11910–1-AP, RRID:AB_11182183, 1:5000), anti-Nucleolin (Ncl) (Abcam, ab50279, RRID:AB_881762, 1:1000), anti-Beta-actin (Actb) (Sigma-Aldrich, A1978, RRID:AB_476692, 1:5000), and anti-Histone H4 (Abcam, ab10158, RRID:AB_296888, 1:4000).<br><br>PRIMARY ANTIBODIES, PLA: anti-biotin (Abcam, ab201341, RRID:AB_2861249, mouse: 1:400), anti-ENO1 (Proteintech, 11204-1-AP, RRID:AB_2099064, rabbit: 1:400), anti-SLC3A2 (Santa Cruz Biotechnology, sc-9160, RRID:AB_638288, rabbit: 1:400), anti-DDX6 (Novus Biologicals, NB200-192, RRID:AB_10000566, rabbit: 1:400), anti-PKM1 (Cell Signaling Technology, 7067, RRID:AB_2715534, rabbit: 1:400).<br><br>SECONDARY ANTIBODIES: anti-rabbit IgG-HRP (Abcam, ab97051, RRID:AB_10679369, 1:5000) or anti-mouse IgG-HRP (Abcam, ab6789, RRID: AB_955439, 1:5000), alpaca nanobody anti-rabbit IgG coupled with Alexa Fluor 488 (Chromotek, srbAF488-1-100, RRID:AB_2827585).
Validation	Primary antibodies were validated by the manufacturers as stated on their web-page and noted below. No further validations were performed.<br><br>anti-ELAVL1/HuR: Lysates from various cell lines (HEK293, HL60, HeLa, Jurkat, RAW 264.7, NIH/3T3) were subjected to SDS-PAGE followed by western blot using this antibody. A single band was observed at the expected molecular weight (MW) (observed MW: 30-37 KDa, calculated MW: 36 KDa) (source: shttps://www.ptglab.com/products/HuR-Antibody-11910-1-AP.htm#product-information).<br><br>anti-Beta-actin: Lysates from various cell lines (HeLa, Jurkat, COS7, NIH/3T3, PC-12, RAT2, CHO, MDBK, MDCK) were subjected to SDS-PAGE followed by western blot using this antibody. A single band was observed at the expected MW (42 KDa) (source: https://www.sigmaaldrich.com/DE/de/product/sigma/a1978).<br><br>anti-Histone H4: Lysates from various cell lines (NIH/3T3, MEF1 and PC12) were subjected to SDS-PAGE followed by western blot using this antibody. One prominent band was observed close to the expected MW (predicted band size: 11 KDa, observed band size: 14 KDa). A band of the same size is detected when using a histone prep, a HeLa histone lysate, a histone prep with human Histone H4 peptide (Abcam, ab13843) or a HeLa histone lysate with human Histone H4 peptide. (source: https://www.abcam.com/histone-h4-antibody-chip-grade-ab10158.html?productWallTab=Abreviews&applications=69&PageSize=10&SortOrder=VoteDesc).

anti-ENO1: Lysates from various cell lines (HeLa, LO2, PC-3, SGC-7901) and tissues (mouse liver, mouse skeletal muscle, human skeletal muscle) were subjected to SDS-PAGE followed by western blot using this antibody. A single band was observed at the expected MW (47 KDa). This antibody was also knockdown validated in HeLa cells using western blotting (source: https://www.ptglab.com/products/ENO1-Antibody-11204-1-AP.htm).

anti-DDX6: Lysates from various cell lines (HeLa, 293T, NIH/3T3) were subjected to SDS-PAGE followed by western blot using this antibody. Bands were observed at the expected MW (54 KDa). This antibody was also knockdown validated using western blotting in nature.com/articles/s41467-019-12238-x. (source: https://www.novusbio.com/products/ddx6-antibody_nb200-192#supportresearch).

anti-PKM1: Western blot analysis of extracts from 293 cells shows a band at the expected MW (60 KDa) when cells are transfected with a construct expressing tagged human PKM1 but not in mock transfected cells. Western blot analysis of extracts from various human tissues (skeletal muscle, small intestine, colon, heart) and mouse skeletal muscle shows a prominent band at the expected MW (60 KDa) (source: https://www.cellsignal.com/products/primary-antibodies/pkm1-d30g6-xp-rabbit-mab/7067).

No validation by manufacturers is shown on the web-page of the following antibodies: anti-Nucleolin (https://www.abcam.com/nucleolin-antibody-ab50279.html), anti-biotin (https://www.abcam.com/biotin-antibody-hyb-8-ab201341.html), anti-SLC3A2 (https://www.scbt.com/p/cd98-antibody-h-300).

Animals and other research organisms

Policy information about studies involving animals; ARRIVE guidelines recommended for reporting animal research, and Sex and Gender in Research

Laboratory animals	Male mice (Mus musculus) on a homogenous C57BL6/J genetic background of 11 to 13 weeks of age were employed. Mice were housed under specific pathogen-free and light- (12:12 hour light:dark cycles), temperature- (21°C), and humidity (50-60% relative humidity)-controlled conditions. Food (Teklad, 2018S) and water were available ad libitum.
Wild animals	The study did not involve wild animals.
Reporting on sex	Male mice were employed.
Field-collected samples	The study did not involve samples collected from the field.
Ethics oversight	Animal handling (license 22-008_HD_LAR) was in accordance with guidelines approved by the animal care and use committee of the European Molecular Biology Laboratory (EMBL).

Note that full information on the approval of the study protocol must also be provided in the manuscript.
